# One-Pot Chemoenzymatic Cascade for the Enantioselective C(1)-Allylation of Tetrahydroisoquinolines

**DOI:** 10.1021/jacs.2c09176

**Published:** 2023-02-15

**Authors:** Jack J. Sangster, Rebecca E. Ruscoe, Sebastian C. Cosgrove, Juan Mangas-Sánchez, Nicholas J. Turner

**Affiliations:** Department of Chemistry, Manchester Institute of Biotechnology, University of Manchester, 131 Princess Street, Manchester M1 7DN, U.K.

## Abstract

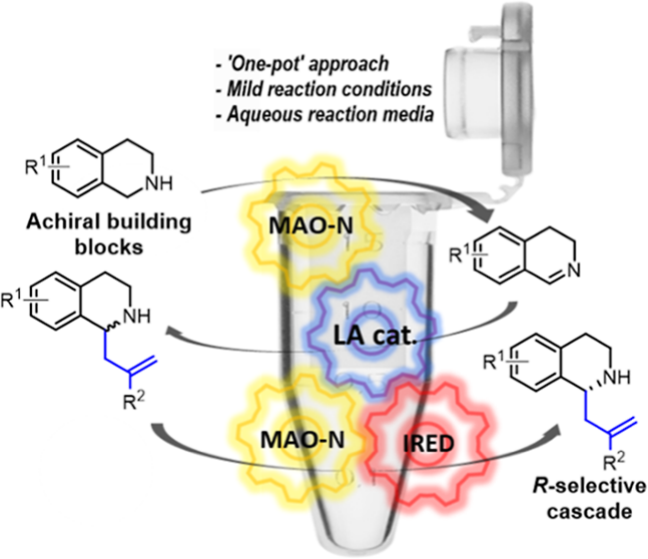

Herein, we report a one-pot, chemoenzymatic process for the synthesis of enantioenriched C(1)-allylated tetrahydroisoquinolines. This transformation couples a monoamine oxidase (MAO-N)-catalyzed oxidation with a metal catalyzed allylboration, followed by a biocatalytic deracemization to afford allylic amine derivatives in both high yields and good to high enantiomeric excess. The cascade is operationally simple, with all components added at the start of the reaction and can be used to generate key building blocks for further elaboration.

## Introduction

Enantiopure amines comprise an important class of organic compounds, in particular heterocyclic amines, which constitute a large number of bioactive molecules.^1^ The prevalence of chiral amine containing compounds in active pharmaceutical ingredients (APIs) is estimated to compromise 40–45% of all drug candidates, which has driven developments in this area.^2^ Traditionally, the main approaches for the stereoselective synthesis of chiral amines involves deracemization,^3^ imine/enamine reduction,^4,5^ transfer hydrogenation,^6^ N–H insertion,^7,8^ reductive amination,^9^ hydroamination,^10^ and nucleophilic addition to imines.^11^ Despite the high atom economy of these catalytic approaches, the requirement for precious metal catalysts, high molecular weight ligands, solvents, and high pressures all adversely impact the overall sustainability.^12^ As more emphasis has been placed on sustainable manufacturing, biocatalysis has emerged as a pre-eminent method for the preparation of chiral amines due to the unique regio-, chemo-, and stereoselectivity conferred by enzymes under benign reaction conditions.^13^ A range of different enzymes have been developed for chiral amine synthesis including amine oxidases (AOs),^14^ imine reductases (IREDs),^14−18^ reductive aminases (RedAms),^19−21^ transaminases (TAs),^14^ amine dehydrogenases (AmDHs),^20,22^ and cytochrome P450 variants (P411s).^23,24^ However, only a small number of enzymes have been characterized which generate chiral amines *via* nucleophilic addition to an imine intermediate, namely, norcoclaurine synthases (NCSs)^25^ and berberine bridge enzymes (BBEs),^26^ both of which are highly substrate dependent.

**Figure 1:**
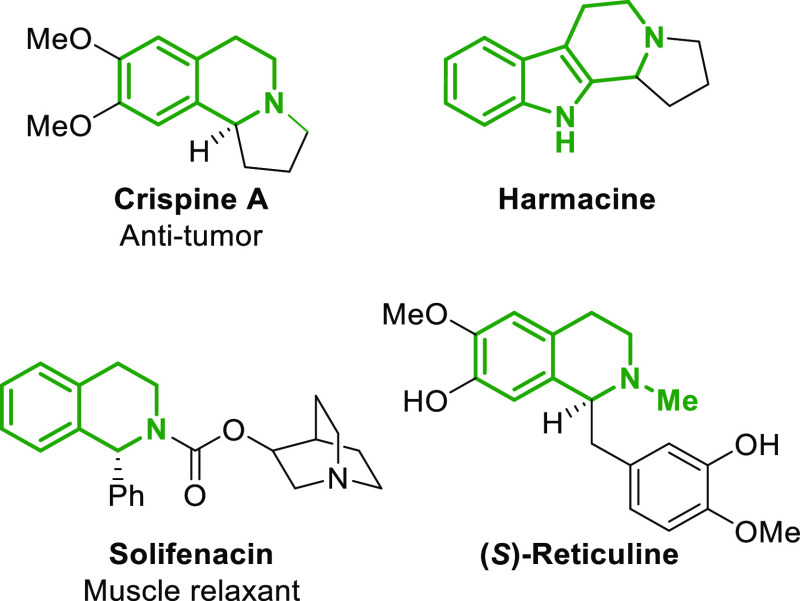
Biologically active compounds containing THIQ core structures.

Although the enantioselective allylation of carbonyls and acyclic imines has been widely reported in the literature,^27,28^ the analogous enantioselective allylation of cyclic imines has received little interest.^29^ Scaffolds such as C(1)-substituted 1,2,3,4-tetrahydroisoquinolines (THIQs) provide compounds with a variety of biological properties, which is highlighted in their use in the pharmaceutical industry (Figure 1).

One such strategy to prepare these compounds involves the oxidative functionalization of N-substituted tetrahydroisoquinolines (THIQs) at the C(1) position. Oss *et al.*, reported a mild method for the C(1)-functionalization of tetrahydroisoquinolines.^30^ The group exploited the tropylium ion to generate an *N*-benzyl/alkyl iminium intermediate, which was quenched by a range of carbon nucleophiles. This approach enabled access to a range of C(1)-substituted products in good to excellent conversion; however, the necessity for *N*-functionalized substrates could limit its application. The work by Yan and co-workers also exploited the oxidative functionalization of N-substituted THIQs for the preparation of 1-allyl THIQs.^31^ The group used triphenylcarbenium tetrafluoroborate to generate the *N*-acyl iminium intermediate, which was intercepted with allyl trimethylsilane. Using this method, several *N*-acyl 1-allyl substituted were prepared in excellent yields. However, the requirement for the stochiometric oxidant negatively impacts the sustainability of this work. Currently, few approaches have been outlined for the stereoselective allylation of tetrahydroisoquinolines. The main approach involves the allylation of cyclic imines through nucleophilic addition of preformed chiral boronates.^32^ A further approach exploits a transition metal catalyst and a chiral ligand to catalyze the allylation stereoselectively.^33^ Although these reports demonstrate enantioselective allylation, they are limited by the requirements of organic solvents, high temperatures, chiral ligands, and preformed imine substrates.

Biocatalytic approaches toward C(1)-functionalized THIQs remain largely unexplored. Norcoclaurine synthases (NCSs) have been exploited by Roddan *et al.*, to access (*S*)-1-aryl tetrahydroisoquinolines.^34^ This simple one-step approach generates the 1-aryl products in good to excellent yield and *ee*, without the need for *N*-functionalization. Erdmann and co-workers also used NCSs in a multi-enzyme cascade toward highly functionalized 1-benzyl/aryl THIQ derivatives.^35^ The three enzyme cascade starting from cheap starting materials furnished either enantiomer of the C(1)-substituted products in excellent yield and *ee*. Currently, only one chemoenzymatic cascade has been reported for the C(1)-functionalization of THIQs. The cascade was developed by Odachowski *et al.*, and involves a MAO-N catalyzed oxidation followed by gold-catalyzed propargylation.^36^ The one-pot approach afforded a range of *N*-methyl-1-propargyl THIQs in good to excellent yields, under mild reaction conditions.

## Results and Discussion

**Scheme 1:**
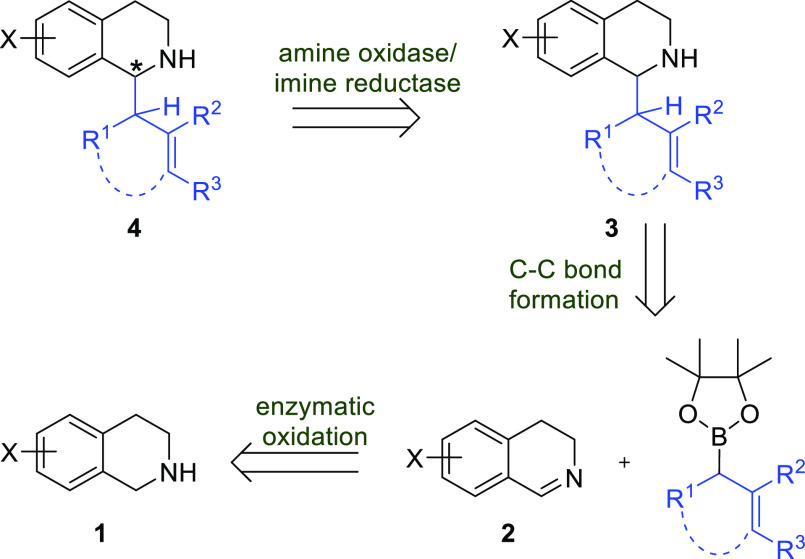
Retrosynthetic Analysis of Proposed Cascade

Following on from our previous work, we envisaged initially employing an enzymatic oxidation reaction to gain access to achiral 3,4-dihydroisoquinolines **2**, which would be utilized in a subsequent chemical C–C bond forming step, to form racemic C(1)-substituted THIQs **3** (Scheme 1). Re-oxidation of this substrate, followed by an enzymatic stereoselective reduction, would allow access to the desired enantio-enriched scaffolds **4**.

To establish the feasibility of the cascade we initially focussed on the model substrate 1,2,3,4-tetrahydroisoquinoline **1a** which has previously been shown to be oxidized to 3,4-dihydroisoquinoline **2a** using variants of monoamine oxidase (MAO-N).^37−46^ For the allylation step involving allylBPin we screened a range of organic solvents and buffers (see Supporting Information for details) and found that the conversion of **2a** to **3a** proceeded well in 100 mM KPi pH 7.8 buffer.

Next, we combined the enzyme catalyzed oxidation and subsequent chemical allylation steps into a one-pot procedure (Table 1). We initially selected four amine oxidases, which included three variants of MAO-N from *Aspergillus niger* along with the 6-hydroxy-d-nicotine oxidase (6-HDNO) from *Arthrobacter nicotinovorans*. The MAO-N variants are all (*S*)-selective enzymes which have been engineered toward the oxidation of pharmaceutically relevant amines. The D5 variant was engineered in our lab to achieve increased activity with both secondary and tertiary amines by increasing the volume of the active site.^47^ Using molecular modeling and saturated mutagenesis, a new variant, MAO-N D9, was developed which showed a 990-fold increase in activity toward Crispine A.^48^ The final MAO-N variant, D11, was engineered toward the oxidation of bulky diaryl chiral amines by increasing the volume of the hydrophobic pocket.^2^ The 6-HDNO variant was developed in our group for the (*R*)-selective oxidation of a broad range of 1, 2, and 3° amines.^49^

**Table 1:**
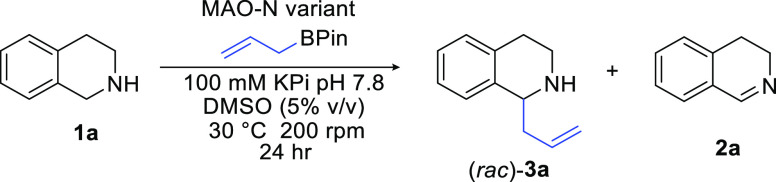
Optimization of Conversion of **1a** to **2a**^a^

entry	enzyme	AllylBPin (equiv)	LA (10 mol %)	**1a** (%)^b^	**2a** (%)^b^	(rac)-**3a** (%)^b^
1	D5^c^	2.5		>99	0	0
2	D9^c^	2.5		21	43	36
3	D9^d^	2.5		27	44	29
4	D9	2.5		0	51	49
5	D9	2.0		0	55	45
6	D9	3.0		0	48	52
7	D9	8.0		0	43	57
8	D9	8.0	InCl_3_	>99	0	0
9	D9	8.0	ZnBr	0	40	60
10	D9	8.0	Cu(OAc)_2_	98	0	2
11	D9	8.0	CuCl_2_	1	0	94
12	D9	8.0	Ag(OTf)_3_	0	50	50
13	D9	8.0	Yb(OTf)_3_	0	<1	>99
14	6-HDNO	8.0	Yb(OTf)_3_	>99	0	0
15	D11	8.0	Yb(OTf)_3_	1	0	99^e^
16		8.0	Yb(OTf)_3_	>99	0	0

a Biotransformations were carried out using 5 mM 1,2,3,4-tetrahydroisoquinoline, 2 mg mL^–1^ purified amine oxidase variant, and 10 mol % of Lewis Acid (LA), and reactions were made up to 500 μL with 5% v/v DMSO and 100 mM KPi buffer corrected to pH 7.8. If not stated enzymes were purified.

b Conversions were based on GCMS data compared to analytic standards.

c 50 mg mL^–1^ MAO-N cell lysate.

d 500 mg mL^–1^ MAO-N whole cell.

e The allylic imine intermediate is not observed in the GCMS. Using chiral HPLC, the conversion of rac-3a is 72 with 26% for the allylic imine intermediate.

Although MAO-N D5 showed no activity, the D9 variant gave 36% conversion to the desired product (*rac*)-**3a** (Table 1, entry 2) and 43% of intermediate imine **2a**. Carrying out the reaction using lyophilized whole cells proved detrimental (Table 1, entry 3), with a decreased conversion to (*rac*)-**3a** of 29%. However, using purified enzyme improved the yield significantly (Table 1, entry 4). Increasing the amount of allylBPin (Table 1, entries 5–7, see Supporting Information for further optimization), improved the conversion to **3a** to 57%.

It has been shown that nucleophilic addition to imines (including allylation reactions) can benefit from the addition of a Lewis acid (LA) leading to higher conversions.^50^ Therefore, we screened a series of water stable LAs (Table 1, entries 8–13) and found that both CuCl_2_ and Yb(OTf)_3_ gave >90% conversion (entries 11 and 13) with no remaining starting material or imine intermediate observed with Yb(OTf)_3_. No product was observed with the (*R*)-selective 6-HDNO (Table 1, entry 14). However, purified MAO-N D11 gave excellent conversions (entry 15). A control experiment in the absence of enzyme (Table 1, entry 16 see Supporting Information for further details) resulted in no conversion to **3a**.

We next turned our attention toward the deracemization of (*rac*)-**3a**.^26,41,51^ which we envisaged could be achieved *via* stereoselective oxidation coupled with either selective or non-selective reduction to enantioenriched-**3a**.

Unfortunately, both the MAO-N D5 and D9 variants were unable to oxidize the racemic product **3a** (Table 2, entries 1 and 2). The (*R*)-selective oxidase, 6-HDNO was also not able to oxidize (*rac*)-**3a** (Table 2, entry 3) which currently limits the cascade to accessing only (*R*)-products. Enzyme engineering could potentially be used to address this issue. Fortunately, the D11 variant succesfully oxidized (*rac*)-**3a** to the corresponding allylic imine intermediate in 26% conversion, favoring formation of (*S*)-**3a** in 36% *ee via* kinetic resolution (Table 2, entry 4). These results for the oxidation of (*rac*)-**3a** using the three MAO-N variants agree with previous reports, where the large hydrophobic site of D11 compared to D5 and D9 is better able to accommodate the bulky bicyclic amine.^43^ Exploiting the activity of MAO-D11, we then investigated a range of chemical and enzymatic reductants (Table 2, entries 4–15). Non-selective chemical reducing agents (entries 5 and 6) showed poor to moderate enantioselectivity for this deracemization process (NH_3_BH_3_, 30% *ee* and NaBH_3_CN, 72% *ee*) with significant amounts of the side-product **4a** being formed. To effectively develop an enzymatic deracemization of the racemic product, the selectivity of the enzymatic oxidation and the IRED catalyzed reduction must be complementary. Thus, we exploited the (*S*)-selective oxidase, MAO-N D11, coupled with a panel of (*R*)-selective IREDs for the reduction step (Table 2, entries 714). The small panel comprised previously characterized and metagenomic IREDs which had displayed high (*R*)-selectivity for the reduction of 2-phenylpiperidine.^16,52^ Pleasingly with *R*-IRED, we observed full conversion to the desired (*R*)-enantiomer (entry 14), with no formation of the fully reduced side-product **4a**, and therefore moved forward with these as our optimal conditions. The absolute configuration of **3a** was confirmed as (*R*)-, as expected, by comparison of the optical rotation value with the previously reported (*S*)-**3a** (see Supporting Information 7.1). All subsequent products were assigned as (*R*)- by analogy, as predicted from the combined use of (*S*)-MAO-N and (*R*)-IRED.

**Table 2:**
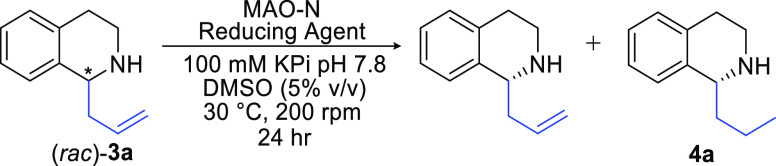
Screening of Reducing Agents From (*rac*)-3a^a^

entry	MAO-N variant	reducing agent	**3a** (%)^d^	**3a** (*R*/*S*)^e^	**4a** (%)^d^
1	D5	none	100	50:50	0
2	D9	none	100	50:50	0
3	6-HDNO	none	100	50:50	0
4	D11	none	74^f^	68:32	0
5	D11	NH_3_·BH_3_^b^	59	65:35	41
6	D11	NaBH_3_CN^b^	71	86:14	29
7	D11	pIRED 229^c^	>99	68:32	<1
8	D11	pIRED 255^c^	81	76:24	19
9	D11	pIRED 282^c^	>99	64:36	<1
10	D11	pIRED 357^c^	98	70:30	2
11	D11	pIRED 170^c^	95	76:24	5
12	D11	pIRED 180^c^	94	75:25	6
13	D11	pIRED 224^c^	99	63:37	1
14	D11	R-IRED^c^	>99	>99:1	<1
15	D11	*Ad*RedAm	86	95:5	14

a Biotransformations were carried out using 5 mM 1,2,3,4-tetrahydroisoquinoline and 2 mg mL^–1^ purified amine oxidase variant, and reactions were made up to 500 μL with 5% v/v DMSO and 100 mM KPi buffer corrected to pH 7.8.

b Biotransformations where chemical reducing agents were used required 2 mg mL^–1^ purified MAO-N D11 and 40 mM of reducing agent.

c Biotransformations where IREDs enzymes were used required 2 mg mL^–1^ purified MAO-N D11 and 6 mg mL^–1^ IRED lysate, 40 mM d-glucose, 0.4 mM NADP^+^, and 1 mg mL^–1^ CDX-GDH.

d Conversions were based on GCMS data compared to analytical standards.

e Measured by chiral HPLC and compared to racemic standards.

f Rest of material observed is oxidized **3a** to the imine intermediate.

**Scheme 2:**
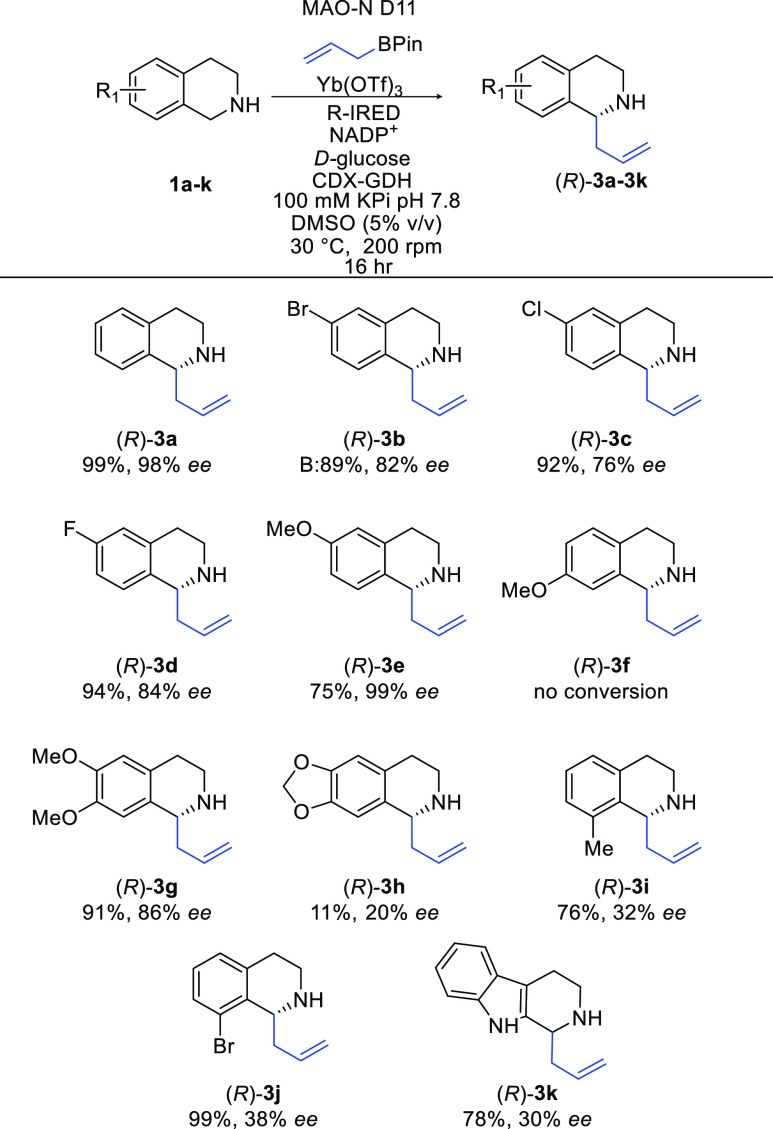
Exploring the Substrate Scope With Respect to the THIQ Starting Material Biotransformations were carried out using 5 mM cyclic amine, 40 mM allylBPin, 10 mol % Yb(OTf)_3_, 2 mg mL^–1^ purified MAO-N D11, 6 mg mL^–1^, R-IRED lysate, 40 mM d-glucose, 0.4 mM NADP^+^, and 1 mg/mL CDX-GDH; reactions were made up to 500 μL with 5% v/v DMSO and 100 mM KPi buffer corrected to pH 7.8. Yields given are determined by GCMS analysis compared to analytical standards. Enantiomeric excess (*ee*) was determined by chiral HPLC analysis and compared to racemic standards.

**Scheme 3:**
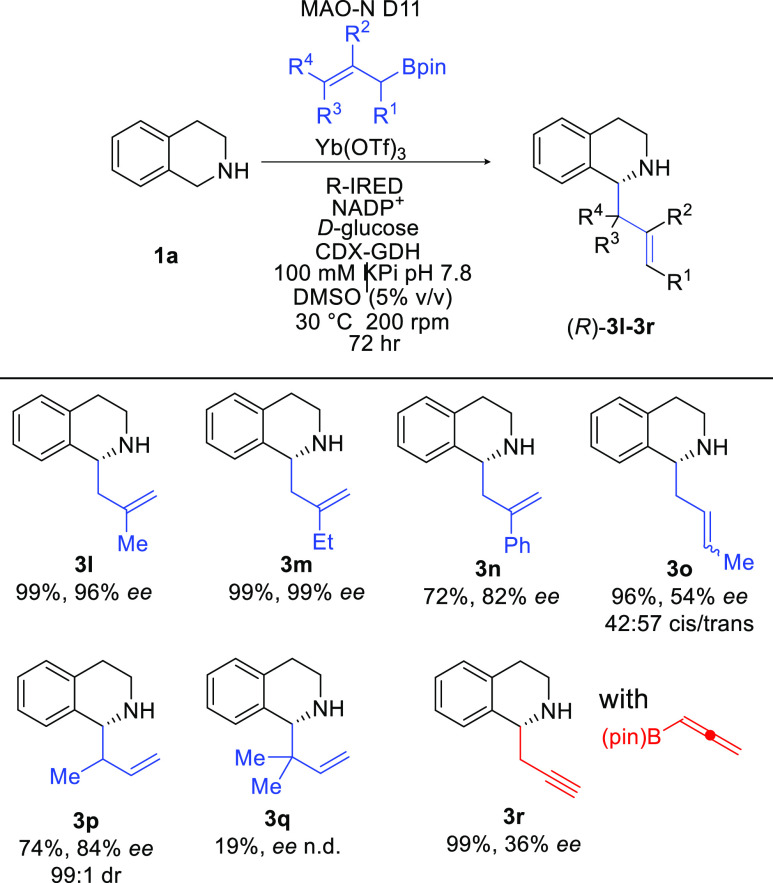
Exploring the Substrate Scope With Respect to the Boryl Reagent Biotransformations were carried out using 5 mM cyclic amine, 40 mM allylBPin derivative, 10 mol % Yb(OTf)_3_, 2 mg mL^–1^ purified MAO-N D11, 6 mg mL^–1^, R-IRED lysate, 40 mM d-glucose, 0.4 mM NADP^+^, and 1 mg mL^–1^ CDX-GDH; reactions were made up to 500 μL with 5% v/v DMSO and 100 mM KPi buffer corrected to pH 7.8. Yields given are determined by GCMS analysis compared to analytical standards. Enantiomeric excess (*ee*) was determined by chiral HPLC analysis and compared to racemic standards.

Next, we explored the substrate scope of the reaction with respect to the tetrahydroisoquinoline (Scheme 2). Subtrates with halogens in the 6-position proceeded well in the reaction, furnishing the desired products in excellent yields and enantioselectivity (**3b–d**), with the fluoro-analogue **3d** providing the best yield and enantioselectivity (94%, 84% *ee*). Electron-donating groups were also compatible under the reaction conditions (**3e**), with the 6-methoxy derivative produced in high yields and enantioselectivity. Unfortunately, the same conversions were not observed when incorporating a methoxy group at the 7-position (Scheme 2, **3f**), with D11 unable to perform the initial oxidation of **1f**. We also attempted the reaction with the 7-bromo-substrate and similarly saw no initial oxidation.

Substitution was tolerated in the 6-7-dimethoxy derivative **3g**, giving rise to the desired product in excellent yields and very good enantioselectivity (91%, 86% *ee*). Compound **3g** is a key intermediate in a synthetic route to (+)-crispine A, a natural alkaloid that has been shown to have anti-tumour activity.^53^ Utilizing this method to synthesize **3g** would avoid the need to use precious metals, which have been published previously.^54−61^ Substitution in the 8-position on the ring were investigated, with a methyl group furnishing the desired product (*R*)-**3i**; however, the enantioselectivity was low (32%). Pleasingly, the 8-bromo product (*R*)-**3j** worked well under the reaction conditions, giving rise to the desired product in excellent yields; however, enantioselectivity remained low (99%, 38% *ee*). The indoline derivative **3k** was formed in good yields (78%) although low enantioselectivity (30% *ee*). This compound is a key intermediate for the synthesis of harmacine.^62^ To address the poor enantioselectivity of **3k**, a broader panel of IREDs was screened in the cascade; however, this had no effect on the *ee*. As the enantioselectivity of the cascade is set through the deracemization, this means the activity and selectivity of both the oxidase and IRED need to be optimal to achieve greater *ee*. As *R*-IRED is a broad and highly selective enzyme, the cascade is currently limited by the D11 variant, further engineering of which will be required to enhance *ee* values.

We then examined the substrate scope with respect to the boryl component (Scheme 3). Pleasingly, when a methyl- or ethyl-group was substituted at R^2^, the reaction proceeded well giving rise to the desired products **3l** and **3m**, respectively. When a phenyl group was substituted at R^2^, the reaction progressed well to give a yield of 72% and very good *ee* (82%) (**3n**). Replacing R^1^ with a methyl group produced the desired product **3o** in excellent yield (96%), moderate enantioselectivity (54%), and gave a mixture of diastereoisomers. When R^4^ is a methyl group (**3p**), the desired product is formed in excellent yield, with excellent diastereo- and enantioselectivity. Introducing two methyl groups at these positions (R^3^ = R^4^ = Me) probed the limit of this reaction with product **3q** being produced in low yields (19%). We were able to incorporate a propargyl group into the products by submitting a simple allene BPin into the reaction, to give compound **3r** in excellent yield (97%); however, the enantioselectivity was moderate. However, the racemic product **3r** has been shown as a key intermediate for the synthesis of reserpine alkaloids that are used to treat hypertension.^63^

**Scheme 4:**
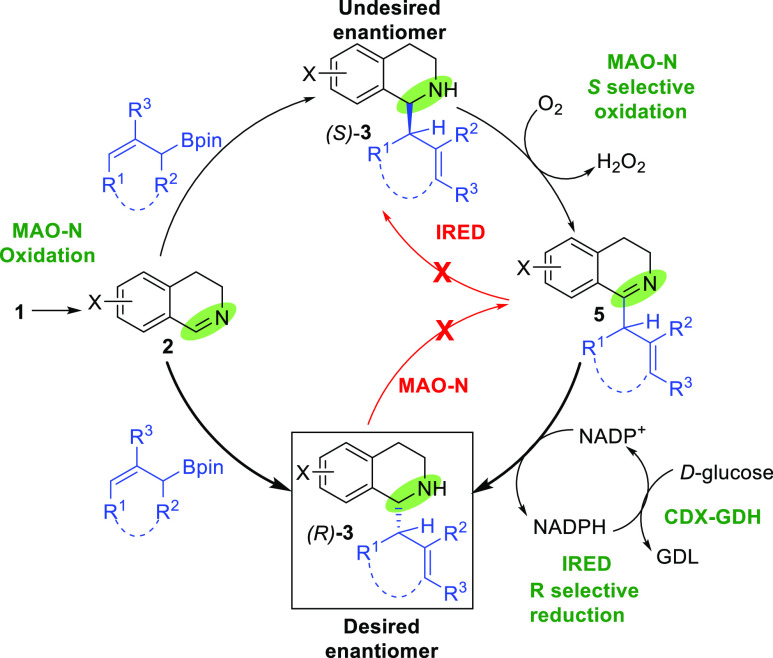
Proposed Catalytic Cycle

To improve our understanding of the reaction, we carried out a series of time course experiments, where we monitored the conversion and enantioselectivity of the formation of 1-allyl-1,2,3,4-tetrahydroisoquinoline **3a** from 1,2,3,4-tetrahydroisoquinoline **1a** over time (see Supporting Information). After only 6 h, the conversion reached 90% with 90% *ee*, and after 12 h, only the desired product and (*R*)-**3a** could be observed (>99% *ee*). The proposed catalytic cycle is shown in Scheme 4.

**Scheme 5:**
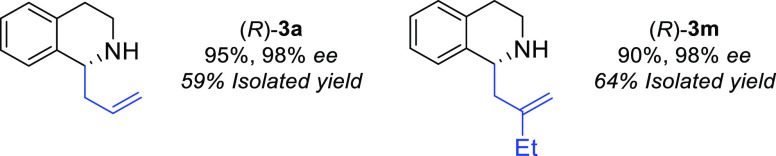
Preparative Scale Reactions Biotransformations were carried out using 15 mM cyclic amine, 120 mM allylBPin derivative, 10 mol % Yb(OTf)3, 2 mg mL^–1^ purified MAO-N D11, 6 mg mL^–1^ R-IRED lysate, 40 mM d-glucose, 0.4 mM NADP^+^, and 1 mg mL^–1^ CDX-GDH; reactions were made up to 50 mL with 5% v/v DMSO and 100 mM KPi buffer corrected to pH 7.8. Conversion given are determined by GCMS analysis compared to analytical standards. Enantiomeric excess (*ee*) was determined by chiral HPLC analysis and compared to racemic standards.

Initial oxidation of the tetrahydroisoquinoline **1** catalyzed by MAO-N is followed by nucleophilic addition of allylboronic acid pinacol ester, in the presence of a Lewis acid, to form a racemic mixture of product **3**. MAO-N then selectively oxidizes the (*S*)-enantiomer of **3** to the corresponding dihydroisoquinoline **5**, which is subsequently reduced selectively to the desired product, (*R*)-**3**. Once formed, either *via* the initial allylation reaction or the oxidation/reduction pathway, the desired *R*-enantiomer remains untouched by MAO-N.

Finally, to demonstrate the practical utility of this chemo-enzymatic allylation process, we carried out preparative scale reactions. Products (*R*)-**3a** and (*R*)-**3m** were successfully isolated from scaled-up reactions in 59 and 64% yield, respectively (98% *ee*) (Scheme 5).

In summary, we have developed a one-pot chemo-enzymatic cascade process that employs two different biocatalysts, namely, a monoamine oxidase (MAO-N) and an imine reductase (IREDs), to enable enantioselective C(1)-allylation of tetrahydroisoquinolines. The cascade has been shown to work with a range of substituted tetrahydroisoquinolines as well as various allylBPin reagents. This report is a major advancement over our previous work on chemoenzymatic cascades toward C(1)-substituted THIQs.^36^ In our earlier approach, *N*-alkylation of the THIQ substrate was required to generate the more reactive iminium intermediate, limiting further downstream modification. Our current approach goes *via* the imine intermediate and thus does not require *N*-alkylation or acylation. However, the key advancement is the ability of this cascade to deracemize the racemic product, obtaining high enantioselectivities. The method offers an alternative approach to access these privileged scaffolds without the requirement of precious metals, organic solvents, and high temperatures.

## Supporting Information Available

Details of experimental procedures, HPLC traces, and NMR spectra (PDF)
