# Supplementary material for: One-Pot Chemoenzymatic Cascade for the Enantioselective C(1)-Allylation of Tetrahydroisoquinolines
Source: J Am Chem Soc. 2023 Feb 15;145(8):4431–7. doi: 10.1021/jacs.2c09176 (PMC9983016; doi:10.1021/jacs.2c09176)
Supplement: Supplementary file 1 — ja2c09176_si_001.pdf [file ja2c09176_si_001.pdf]

One-Pot Chemoenzymatic Cascade for the Enantioselective C(1)-Allylation of Tetrahydroisoquinolines

Jack J. Sangster,[†] Rebecca E. Ruscoe,[†] Sebastian C. Cosgrove,[†‡] Juan Mangas-Sánchez,[†§] Nicholas J. Turner[*†]

[†] Department of Chemistry, University of Manchester, Manchester Institute of Biotechnology, 131 Princess Street, Manchester M1 7DN, UK.

[‡] Current address: Lennard-Jones Laboratory, School of Chemical and Physical Sciences, Keele University, Keele, Staffordshire, ST5 5BG, UK.

[§] Current address: Aragonese Foundation for Research & Development (ARAID). Institute of Chemical Synthesis and Homogeneous Catalysis (ISQCH-CSIC). Pedro Cerbuna 12, 50009 Zaragoza, Spain.

SUPPORTING INFORMATION

Contents

1. General methods ... 5
2. Preparation of biocatalysts ... 6
   2.1. Biocatalyst gene sequences ... 6
   2.2. General procedure for the recombinant expression of monoamine oxidase biocatalysts ... 6
   2.3. General procedure for the purification of monoamine oxidase biocatalysts ... 6
   2.4. General procedure for the recombinant expression of imine reductase biocatalysts ... 7
   2.5. General procedure for the preparation of enzyme lysate ... 7
3. Synthetic methods and characterisation ... 8
   3.1. Chemical oxidation of substituted tetrahydroisoquinolines ... 8
      6-Bromo-3,4-dihydroisoquinoline, 2b ... 8
      6-Chloro-3,4-dihydroisoquinoline, 2c ... 8
      6--Fluoro-3,4-dihydroisoquinoline, 2d ... 8
      6-Methoxy-3,4-dihydroisoquinoline, 2e ... 9
      7,8-Dihydro-[1,3]dioxolo[4,5-g]isoquinoline 2h ... 9
      8-Methyl-3,4-dihydroisoquinoline, 2i ... 9
      8-Bromo-3,4-dihydroisoquinoline, 2j ... 10
   3.2. Chemical oxidation of 1,2,3,4-Tetrahydro-9*H*-pyrido[3,4-*b*]indole ... 11
      4,9-Dihydro-*3H*-pyrido[3,4-b]indole, 2k ... 11
   3.3. Preparation of 1-allyl-tetrahydroisoquinoline derivatives ... 12
      1-Allyl-1,3,4-tetrahydroisoquinoline, (*rac*)-3a ... 12
      1-Allyl-6-bromo-1,3,4-tetrahydroisoquinoline, (*rac*)-3b ... 12
      1-Allyl-6-chloro-1,3,4-tetrahydroisoquinoline, (*rac*)-3c ... 13
      1-Allyl-6-fluoro-1,3,4-tetrahydroisoquinoline, (*rac*)-3d ... 13
      1-Allyl-6-methyl-1,3,4-tetrahydroisoquinoline, (*rac*)-3e ... 14
      5-Allyl-5,6,7,8-[1,3]dioxolo[4,5-g]isoquinoline, (*rac*)-3h ... 14
      1-Allyl-8-methoxy-1,3,4-tetrahydroisoquinoline, (*rac*)-3i ... 14
      1-Allyl-8-bromo-1,3,4-tetrahydroisoquinoline (*rac*)-3j ... 15
      1-Allyl-2,3,4,9-tetrahydro-1H-pyrido[3,4-b]indole, (*rac*)-3k ... 15
   3.4. Alternative method for the preparation of 1-allyl tetrahydroisoquinolines ... 16
      1-Allyl-6,7-dimethoxy-1,3,4-tetrahydroisoquinoline, (*rac*)-3g ... 16
   3.5. Preparation of allylic Grignards ... 17
   3.6. Preparation of 1-substituted tetrahydroisoquinolines ... 17
      1-(2-Methylallyl)-1,2,3,4-tetrahydroisoquinoline, (*rac*)-3l ... 17
      1(2-Phenylallyl)-1,2,3,4-tetrahydroisoquinoline, (*rac*)-3n ... 17
      1-(But-2-en-1-yl)-1,2,3,4-tetrahydroisoquinoline, (*rac*)-3o ... 18
      1-(But-3-en-2-yl)-1,2,3,4-tetrahydroisoquinoline, (*rac*)-3p ... 18
   3.7. Alternative method for the preparation of 1-substituted tetrahydroisoquinolines ... 19
      1-(2-Methylenebutyl)-1,2,3,4-tetrahydroisoquinoline, (*rac*)-3m ... 19
      1-(Prop-2-yn-1-yl)-1,2,3,4-tetrahydroisoquinoline, (*rac*)-3r ... 19
   3.8. Synthesis of substituted allyl boronic acid pinacol esters ... 20
4. Analytical scale optimisation of chemical allylation ... 22
   4.1. Solvent Screening for the chemical allylation of 3,4-dihydroisoquinoline ... 22
   4.2. Optimisation of the chemical allylation of 3,4-dihydroisoquinoline ... 22

5. Biotransformations 23
5.1. Analytical scale procedure for the chemoenzymatic allylation of tetrahydroisoquinoline using whole-cell biocatalyst...23
5.2. Analytical scale procedure for the chemoenzymatic allylation of tetrahydroisoquinoline using freeze-dried enzyme lysate 23
5.3. Analytical scale procedure for the chemoenzymatic allylation of tetrahydroisoquinoline using purified enzyme....23
5.4. Analytical scale procedure for the chemoenzymatic allylboration of tetrahydroisoquinoline using purified enzyme biocatalysts and L.A metal catalyst....23
5.5. Analytical scale procedure for the enzymatic deracemisation of (*rac*)-3a using non-selective chemical reducing agents24
5.6. Analytical scale procedure for the screening of IREDs for the deracemisation of (*rac*)-3a 24
5.7. Analytical scale procedure for the enantioselective allylation of cyclic amines 24
5.8. Analytical scale procedure for the enantioselective addition of allylic BPin derivatives to cyclic amines 24
6. Chiral HPLC and GC.MS data 25
6.1. Chiral HPLC: methods and conditions 25
6.2. Absolute configuration 25
6.3. Chiral HPLC and GC.MS data for the MAO-N D11 catalysed kinetic resolution of (*rac*)-3a 25
6.4. Chiral HPLC and GC.MS data for analytical biotransformations 26
(*R*)-1-Allyl-1,2,3,4-tetrahydroisoquinoline, (*R*)-3a 26
(*R*)-1-Allyl-6-bromo-1,2,3,4-tetrahydroisoquinoline, (*R*)-3b 27
(*R*)-1-Allyl-6-chloro-1,2,3,4-tetrahydroisoquinoline, (*R*)-3c 28
(*R*)-1-Allyl-6-fluoro-1,2,3,4-tetrahydroisoquinoline, (*R*)-3d 29
(*R*)-1-Allyl-6-methoxy-1,2,3,4-tetrahydroisoquinoline, (*R*)-3e 30
(*R*)-1-Allyl-6,7-dimethoxy-1,2,3,4-tetrahydroisoquinoline, (*R*)-3g 31
(*R*)- 5-Allyl-5,6,7,8-tetrahydro-[1,3]dioxolo[4,5-g]isoquinoline, (*R*)-3h 32
(*R*)-1-Allyl-8-methyl-1,2,3,4-tetrahydroisoquinoline, (*R*)-3i 33
(*R*)-1-Allyl-8-bromo-1,2,3,4-tetrahydroisoquinoline, (*R*)-3j 34
(*R*)- 1-Allyl-2,3,4,9-tetrahydro-1H-pyrido[3,4-b]indole, (*R*)-3k 35
(*R*)- 1-(2-Methylallyl)-1,2,3,4-tetrahydroisoquinoline, (*R*)-3l 36
(*R*)-1-(2-Methylenebutyl)-1,2,3,4-tetrahydroisoquinoline, (*R*)-3m 37
(*R*)- 1-(2-Phenylallyl)-1,2,3,4-tetrahydroisoquinoline, (*R*)-3n 38
(*R*)- 1-(But-2-en-1-yl)-1,2,3,4-tetrahydroisoquinoline, (*R*)-3o 39
1-(But-3-en-2-yl)-1,2,3,4-tetrahydroisoquinoline, (*R*,*S*)-3p 40
(*R*)-1-(2-Methylbut-3-en-2-yl)-1,2,3,4-tetrahydroisoquinoline, 3q 42
(*R*)-1-Prop-2-yn-1-yl-1,2,3,4-tetrahydroisoquinoline, (*R*,*S*)-3r 43
7. Preparative scale biotransformations 44
7.1. Chemoenzymatic synthesis of (*R*)-1-allyl-1,2,3,4-tetrahydroisoquinoline, (*R*)-3a 44
7.2. Chemoenzymatic synthesis of 1-(2-methylenebutyl)-1,2,3,4-tetrahydroisoquinoline, (*R*)-3l 44
8. Time course Experiments 45
9. NMR analysis 46
9.1. NMR spectra of novel chemical standards 46
1-Allyl-6-bromo-1,2,3,4-tetrahydroisoquinoline, (*rac*)-3b 46
1-Allyl-6-chloro-1,2,3,4-tetrahydroisoquinoline, (*rac*)-3c 47
1-Allyl-6-methoxy 1,2,3,4-tetrahydroisoquinoline, (*rac*)-3e 48
1-Allyl-8-methyl-1,2,3,4-tetrahydroisoquinoline, (*rac*)-3i 49
1-Allyl-8-bromo-1,2,3,4-tetrahydroisoquinoline, (*rac*)-3j 50
1-Allyl-8-bromo-1,2,3,4-tetrahydroisoquinoline, (*rac*)-3j 51

1-(2-Methylallyl)-1,2,3,4-tetrahydroisoquinoline, (*rac*)-3l 52
1-(2-Methylenebutyl)-1,2,3,4-tetrahydroisoquinoline, (*rac*)-3m 53
1-(2-Phenylallyl)-1,2,3,4-tetrahydroisoquinoline, (*rac*)-3n 54
1-(2-Methylenebutyl)-1,2,3,4-tetrahydroisoquinoline, (*rac*)-3o 55
1-(But-3-en-2-yl)-1,2,3,4-tetrahydroisoquinoline, (*rac*)-3p...56
10. References 57

1. General methods

Commercially available chemicals and reagents were purchased from Sigma-Aldrich (Poole, Dorset, UK), Prozomix (Haltwhistle, Northumberland, UK), Alfa Aesar (Karlsruhe, Germany), Acros Organics (Geel, Belgium) or Fluorochem Limited (Hadfield, Derbyshire, UK) and used without further purification. HPLC solvents were obtained from Sigma-Aldrich (Poole, Dorset, UK) or ROMIL (Waterbeach, Cambridge, UK). Competent *E. coli* BL21(DE3) cells were purchased from New England Biolabs (NEB) and transformations were carried out using the supplied NEB protocol. Column chromatography was performed on silica gel (Fluka (Buchs, Switzerland), 220-440 mesh). Spectra from ^{1}H and ^{13}C NMR runs were recorded on a Bruker Advance 400 instrument (400 MHz ^{1}H and 101 MHz for ^{13}C) in $CDCl_3$ using residual protic solvent as an internal standard. Reported chemical shifts (δ) (in parts per million (ppm)) are relative to the residual protic solvent signal ($CHCl_3$ in $CDCl_3$, ^{1}H = 7.26; ^{13}C = 77.0). Chiral HPLC was performed on an Agilent system (Santa Clara, CA, USA) equipped with a G1379A degasser, G1312A binary pump, a G1367A well plate autosampler unit, a G1316A temperature controlled column compartment and a G1315C diode array detector. CHIRALPAK®IA, CHIRALPAK®IC and CHIRALPAK®IE Analytical (all Daicel (Osaka, Japan), 250 mm length, 4.6 mm diameter, 5 µm particle size) as well as CHIRALCEL®OD-H Analytical (Daicel (Osaka, Japan), 250 mm length, 4.6 mm diameter, 5 µm particle size) columns were used. The typical injection volume was 10 µl and chromatograms were monitored at 265 nm, unless stated otherwise. All solvent mixtures are given in (v/v) ratios. GC analysis was performed on an Agilent 6850 GC (Agilent, Santa Clara, CA, USA) with a flame ionization detector (FID) and autosampler. Columns used include a 25 m CP-Chirasil-DEX CB column with 0.25 mm inner diameter and 0.25 µm film thickness (Agilent, Santa Clara, CA, USA); a 30 m β- DEX-325 with 0.25 mm inner diameter and 0.25 µm film thickness (Supeclo, Bellefont, PA, USA); a 30 m HP-1MS column with 0.32 mm inner diameter and 0.25 µm film thickness (Agilent, Santa Clara, CA, USA) and an HP-1 column with 0.32 mm inner diameter and 0.25 µm film thickness (Agilent, 6 Santa Clara, CA, USA). GCMS analysis was performed on a HP-6890 Series GC coupled to a HP5973 MS detector, EI positive mode. Commercially available biocatalysts were provided as CFEs from Prozomix (PRO-IREDXX), UK and Johnson Matthey (JMENEXXX), UK. MAO-N variants and *R*-IRED were expressed in house.

2. Preparation of biocatalysts

2.1. Biocatalyst gene sequences

Enzyme	Plasmid	Source	Reference
MAO-N D5	pET16a-MAO-ND5	*Aspergillus niger*	S[1]
MAO-N D9	pET16a-MAO-ND9	*Aspergillus niger*	S[2]
MAO-N D11	pET16a-MAO-ND11	*Aspergillus niger*	S[2]
R-IRED	pET28a-*R*IRED	*Streptomyces sp. GF3587*	S[3]
*Asp*RedAm	pET28a-*Asp*RedAm	*Aspergillus oryzae*	S[4]
6-HDNO	pET16a-6HDNO	*Arthrobacter nicotinovorans*	S[5]

2.2. General procedure for the recombinant expression of monoamine oxidase biocatalysts

MAO-N variants (MAO-N D5, MAO-N D9 and MAO-N D11) were transformed into BL21 (DE3) competent *E. coli* (NEB) following manufacturer's instructions. A single colony was used to inoculate a pre-culture (6 mL) (containing LB medium and ampicillin 100 mg/mL) which was grown at 37 °C and 200 rpm for 12 hours (OD_{600} between 0.6-1.0). 2 L Erlenmeyer flasks containing 600 mL auto-induction media and ampicillin (100 mg/mL) were inoculated with 6 mL of pre-culture and incubated following **Table 1**. The cells were harvested by centrifugation at 4000 rpm at 4 °C for 30 minutes. The pelleted cells were stored at -20 °C before using.

MAO-N Variant	Temperature (°C)	Time (hours)	Shaking (rpm)
MAO-N D5	20	72	200
MAO-N D9	25	48	200
MAO-N D11	20	72	200

2.3. General procedure for the purification of monoamine oxidase biocatalysts

5 g of frozen cell pellet was defrosted on ice and resuspended in 25 mL of buffer A (100 mM KPi, pH 7.8, 300 mM NaCl, 30 mM imidazole) containing lysozyme from chicken egg white (1 mg/mL) and incubated at 30 °C for 30 minutes. The suspension was cooled on ice and the cells lysed by ultra-sonocation (20 s on, 20 s off: 20 cycles). The cells were concentrated by centrifugation (18000 rpm, 45 minutes). Subsequently, the cell free extracts were filtered through a syringe with 0.4 µm followed by 0.22 µm pore sizes. The filtered cell-free extracts were loaded onto a HisTrap Ni-sepharose column (1 mL, GE Healthcare) pre-equilibrated with buffer A (100 mM KPi pH 7.8, 300 mM NaCl, 30 mM imidazole). The loaded column was washed with 5 CV (column volumes) of buffer A before eluting with buffer B (100 mM KPi pH 7.8, 300 mM NaCl, 500 mM imidazole) and collecting 1 mL fractions. The enzyme containing fractions (measured using NanoDrop™ spectrophotometer at 280 nm) were pooled before concentrating with Vivaspin® (20 mL, 30 kDa MCO, GE Healthcare) and desalted by eluting with 100 mM KPi pH 7.8. The purified enzymes were snap-frozen in liquid nitrogen and stored at -80 °C before use.

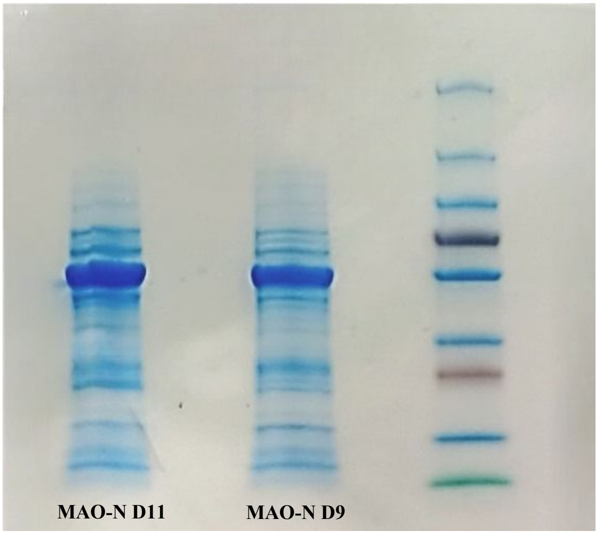

Figure 1. SDS-Page analysis of the expression of MAO-N D9 and MAO-N D11. Lane 1: MAO-N D11 cell free extract. Lane 2: MAO-N D9 cell free extract. Lane 3: Lane marker 10 kDa, 15 kDa, 25 kDa, 35 kDa, 55, kDa, 70 kDa, 100 kDa, 130 kDa, 250 kDa.

2.4. General procedure for the recombinant expression of imine reductase biocatalysts

IRED variants were transformed into BL21 (DE3) competent *E. coli* (NEB) following manufacturer's instructions. A single colony was used to inoculate a pre-culture (20 mL) (containing LB medium and kanamycin 50 mg/mL), which was grown at 37 °C and 200 rpm for 12 hours (OD_{600} between 0.6-1.0). 2 L Erlenmeyer flasks containing 400 mL auto-induction media, kanamycin (50 mg/mL) and 1.6 mL glycerol were inoculated with 20 mL of pre-culture and incubated at 37 °C untill reaching an OD_{600} of 0.6. After this time IPTG (0.1 mmol) was added and the flasks left to grow at 25 °C overnight. After this time, the cells were harvested by centrifugation at 4000 rpm at 4 °C for 30 minutes. The pelleted cells were stored at -20 °C before using.

2.5. General procedure for the preparation of enzyme lysate

5 g of frozen cell pellet was defrosted on ice and resuspended in 25 mL of phosphate buffer (100 mM KPi pH 7.8) containing lysozyme from chicken egg white (1 mg/mL) and incubated at 30 °C for 30 minutes. The suspension was cooled on ice and the cells lysed by ultra-sonocation (20 s on, 20 s off: 20 cycles). The cells were concentrated by centrifugation (18000 rpm, 45 minutes). Subsequently, 20 mL of cell-free extract was transferred to a 50 mL falcon tube. The falcon tube was snap frozen in liquid nitrogen prior to freeze-drying for 1-2 days (until powdery). The freeze-dried lysate was stored at -20 °C prior to use.

3. Synthetic methods and characterisation

3.1. Chemical oxidation of substituted tetrahydroisoquinolines

General procedure: To a solution of 1,2,3,4-tetrahydroisoquinoline (1.0 equiv.) in anhydrous DCM at r.t under nitrogen was added *N*-bromosuccinimide (NBS) (3.0 equiv.). The reaction mixture was stirred at r.t for 1 hour. Afterwards, 30% v/v NaOH aqueous solution was added and left to react for a further 1 hour at r.t. After which time, the aqueous phase was extracted with DCM (3 x 5 mL). The combined organic layers were dried over anhydrous $MgSO_4$, filtered and concentrated *in vacuo*.

6-Bromo-3,4-dihydroisoquinoline, 2b

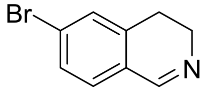

6-Bromo-1,2,3,4-tetrahydroisoquinoline (150 mg, 0.70 mmol) was reacted with *N*-bromosuccinimide (NBS) (373 mg, 2.10 mmol) at r.t under nitrogen. After this time, 1 mL of 30% v/v NaOH aqueous solution was added and left to react for a further 1 hour at r.t. Extraction with DCM afforded the desired product (121 mg, 0.57 mmol, 82%) as a yellow oil without further purification.

^{1}H NMR (400 MHz, $CDCl_3$) δ 8.31 (s, 1H, HC=N), 7.46 (app. t, *J*= 8.0 Hz, 1H, ArCH), 7.34 (s, 1H, ArCH), 7.10 (d, *J*= 8.41 Hz, 1H, ArCH), 3.67 (m, 2H, CH_2), 2.69 (m, 2H, CH_2). Data consistent with the literature.[6]
MS (ESI) (m/z) = 209.9879 ($M+H^+$), calc. for C_9H_9BrN 209.9840.

6-Chloro-3,4-dihydroisoquinoline, 2c

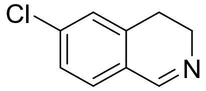

6-Chloro-1,2,3,4-tetrahydroisoquinoline (150 mg, 0.89 mmol) was reacted with *N*-bromosuccinimide (NBS) (475 mg, 2.67 mmol) at r.t under nitrogen. After this time, 1 mL of 30% v/v NaOH aqueous solution was added and left to react for a further 1 hour at r.t. Extraction with DCM afforded the desired product (119 mg, 0.71 mmol, 81%) as a brown/yellow oil without further purification.

^{1}H NMR (400 MHz, $CDCl_3$) δ 8.24 (s, 1H, HC=N), 7.22 (app. dd, *J*= 6.2, 2.1 Hz, 1H, ArCH), 7.11 (m, 2H, ArCH), 3.69 (ddd, *J*=9.9, 6.3, 2.2 Hz, 2H, CH_2), 2.67 (t, *J*= 8.1, 7.5 Hz, 2H, CH_2). Data consistent with the literature.[6]
MS (ESI) (m/z) = 166.0425 ($M+H^+$), calc. for C_9H_9ClN 166.0345.

6-Fluoro-3,4-dihydroisoquinoline, 2d

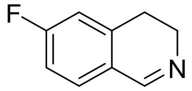

6-Fluoro-1,2,3,4-tetrahydroisoquinoline (150 mg, 0.99 mmol) was dissolved in 4 mL anhydrous DCM and reacted with *N*-bromosuccinimide (NBS) (529 mg, 2.97 mmol) at r.t under nitrogen for 1 hour. After this time, 1 mL of 30% v/v NaOH aqueous solution was added and left to react for a further 1 hour at r.t. Extraction with DCM afforded the desired product (127 mg, 1.15 mmol 87%) as a brown oil without further purification.

^{1}H NMR (400 MHz, $CDCl_3$) δ 8.22 (s, 1H, HC=N), 7.20 (app. dd, *J*= 5.7, 5.2 Hz, 1H, ArCH), 6.90 (app. td, *J*= 8.6, 2.6 Hz, 1H, ArCH), 6.81 (d, *J*= 8.6 Hz, 1H, ArCH), 3.68 (ddd, *J*= 8.3, 6.2, 2.1 Hz, 2H, CH_2), 2.65 (d, *J*= 7.7 Hz, 2H, CH_2). Data consistent with the literature.[7]
MS (ESI) (m/z) = 166.0425 ($M+H^+$), calc. for C_9H_9FN 149.0641.

7,8-Dihydro-[1,3]dioxolo[4,5-*g*]isoquinoline, 2h

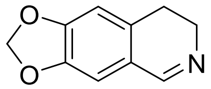

5,6,7,8-Terahydro-[1,3]dioxolo[4,5-*g*]isoquinoline (200 mg, 1.13 mmol) was reacted with *N*-bromosuccinimide (NBS) (603 mg, 3.39 mmol) at r.t under nitrogen. After this time, 1 mL of 30% v/v NaOH aqueous solution was added and left to react for a further 1 hour at r.t. Extraction with DCM afforded the desired product (97 mg, 0.55 mmol, 49%) as a dark yellow oil without further purification.

^{1}H NMR (400 MHz, $CDCl_3$) δ 8.25 (s, 1H, HC=N), 6.86 (s, 1H, ArCH), 6.71 (s, 1H, ArCH), 6.02 (s, 2H, CH_2), 3.59-3.62 (m, 2H, CH_2), 2.52-2.58 (m, 1H, CH_2). Data consistent with the literature.[8]
MS (ESI) (m/z) = 176.0715 ($M+H^+$), calc. 176.0633.

6-Methoxy-3,4-dihydroisoquinoline, 2e

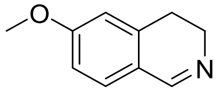

6-Methoxy-1,2,3,4-tetrahydroisoquinoline (150 mg, 0.92 mmol) was reacted with *N*-bromosuccinimide (NBS) (491 mg, 2.76 mmol) at r.t under nitrogen. After this time, 1 mL of 30% v/v NaOH aqueous solution was added and left to react for a further 1 hour at r.t. Extraction with DCM afforded the desired product (131 mg, 0.81 mmol, 88%) as a yellow oil without further purification.

^{1}H NMR (400 MHz, $CDCl_3$) δ 8.30 (br. s, 1H, HC=N), 7.44 (d, *J*= 5.8 Hz, 1H, ArCH), 7.32 (s, 1H, ArCH), 7.14 (d, *J*= 8.0 Hz, 1H, ArCH), 5.29 (s, 3H, CH_3), 3.76 (ddd, *J*= 10.0, 6.3, 2.2 Hz, 2H, CH_2), 2.73 (m, 2H. CH_2). Data consistent with the literature.[7]
MS (ESI) (m/z) = 162.0924 ($M+H^+$), calc. 162.0841.

8-Methyl-3,4-dihydroisoquinoline, 2i

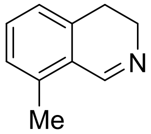

8-Methyl-1,2,3,4-tetrahydrohydroisoquinoline (150 mg, 1.02 mmol) was reacted with *N*-bromosuccinimide (NBS) (545 mg, 3.06 mmol) at r.t under nitrogen. After this time, 1 mL of 30% v/v NaOH aqueous solution was added and left to react for a further 1 hour at r.t. Extraction with DCM afforded the desired product (139 mg, 0.96 mmol 79%) as a brown oil without further purification.

^{1}H NMR (400 MHz, $CDCl_3$) δ 8.65 (s, 1H, HC=N), 7.22 (t, *J*=7.5 Hz, 1H, ArCH), 7.02 (dd, *J*= 11.6, 7.5 Hz, 2H, ArCH), (ddd, *J*= 9.8, 6.2, 2.0 Hz, 2H, CH_2), 2.69 (dd, *J*= 9.8, 6.5 Hz, 2H, CH_2), 2.46 (s, 3H, CH_3). Data consistent with the literature.[7]
MS (ESI) (m/z) = 146.0973 ($M+H^+$), calc. 146.0891.

8-Bromo-3,4-dihydroisoquinoline, 2j

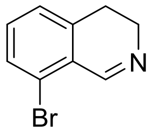

8-Bromo-1,2,3,4-tetrahydroisoquinoline (200 mg, 0.94 mmol) was reacted with *N*-bromosuccinimide (NBS) (502 mg, 2.82 mmol) at r.t under nitrogen. After this time, 1 mL of 30% v/v NaOH aqueous solution was added and left to react for a further 1 hour at r.t. Extraction with DCM afforded the desired product (168 mg, 0.80 mmol, 85%) as a yellow oil without further purification.

^{1}H NMR (400 MHz, $CDCl_3$) δ 8.60 (s, 1H, HC=N), 7.39 (d, *J*= 9.0 Hz, 1H, ArCH), 7.02-7.15 (m, 2H, ArCH), 3.68 (ddd, *J*= 9.8, 6.0, 2.3 Hz, 2H, CH_2), 2.64 (m, 2H, CH_2). Data consistent with the literature.[6]
MS (ESI) (m/z) = 209.9879 ($M+H^+$), calc. 209.9840

3.2. Chemical oxidation of 1,2,3,4-Tetrahydro-9*H*-pyrido[3,4-*b*]indole

2-Iodoxybenzoic acid (IBX) (1.3 equiv.) was dissolved in DMSO and stirred for 30 min at r.t. Then 2,3,4,9-tetrahydro-1H-pyrido[3,4-b]indole (1.0 equiv.) was added and the reaction stirred at r.t for 1 hr. The reaction mixture was quenched with sat. $Na_2S_2O_3$ and basified with sat. $NaHCO_3$. The mixture was then extracted with EtOAc (3 x 5 mL) and the organic layer subsequently washed with water and brine. The combined organic layers were dried over anhydrous $MgSO_4$, filtered and concentrated *in vacuo*.

4,9-Dihydro-*3H*-pyrido[3,4-b]indole, 2k

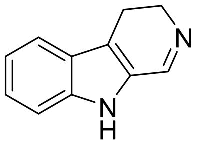

2,3,4,9-Tetrahydro-1H-pyrido[3,4-b]indole (200 mg, 1.16 mmol) was reacted with 2-Iodoxybenzoic acid (IBX) (423 mg, 1.51 mmol) at r.t. Extraction with DCM afforded the desired product (158 mg, 0.93 mmol, 80%) as a brown oil without further purification.

^{1}H NMR (400 MHz, $CDCl_3$) δ 8.38 (s, 1H, N-H), 8.35 (s, 1H, HC=N), 7.74 (d, *J*= 8.2 Hz, 1H, ArCH), 7.40-7.54 (m, 1H, ArCH), 7.36 (ddd, *J*= 8.2, 4.3, 1.0 Hz, 1H, ArCH), 7.08 (ddd, J= 8.6, 6.9, 1.2 Hz, 1H, ArCH), 4.12 – 3.96 (m, 2H, CH_2), 2.89 – 2.74 (m, 2H, CH_2). Data consistent with the literature.[9]
MS (ESI) (m/z) = 171.0924 ($M+H^+$), calc. 171.0844.

3.3. Preparation of 1-allyl-tetrahydroisoquinoline derivatives

To a solution of 3,4-dihydroisoquinoline (1.0 equiv.) in anhydrous Et_2O at r.t under nitrogen was added boron trifluoride dietherate ($BF_3.OEt_2$) (2.0 equiv.) followed by allyl magnesium bromide (0.5M in Et_2O) (1.o equiv.). The reaction was left to stir at r.t under N_2 overnight. After this time, the reaction was quenched by addition of water and left to stir for a further 1 hr. Subsequently, the mixture was extracted with EtOAc (3 x 5 mL), dried over anhydrous $MgSO_4$, filtered and concentrated *in vacuo*.

1-Allyl-1,3,4-tetrahydroisoquinoline, (*rac*)-3a

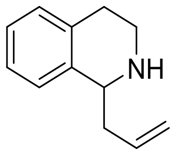

3,4-Dihydroisoquinoline (100 mg, 0.76 mmol) was reacted with boron trifluoride dietherate (1.52 mmol, 0.19 mL) and allyl magnesium bromide (0.5M in Et_2O, 1.52 mL, 0.76 mmol) at r.t under nitrogen. Purification over silica gel (20% v/v methanol:DCM) afforded the desired product (71 mg, 0.41 mmol, 55%) as a brown oil.

^{1}H NMR (400 MHz, $CDCl_3$) δ 7.19-7.07 (m, 4H, ArCH), 5.84 (dddd, J= 16.8, 10.2, 7.8, 6.4 Hz, 1H, CH=), 5.17 (m, 2H, =CH_2), 4.06 (dd, J= 9.0, 3.7 Hz, 1H, CH), 3.24 (ddd, J= 12.5, 7.8, 5.0 Hz, 1H, CH_2), 2.97 (ddd, J= 12.5, 7.8, 5.0 Hz, 1H, CH_2), 2.81 (m, 2H, CH_2), 2.68 (dddd, J= 13.1, 6.5, 3.6, 1.7 Hz, 1H, CH_2), 2.53 (m, 1H, CH_2), 2.28 (br. s, 1H, NH). Data consistent with the literature.[9]
MS (ESI) (m/z) = 174.1279 (M+H$^+$), calc. 174.1204.

1-Allyl-6-bromo-1,3,4-tetrahydroisoquinoline, (*rac*)-3b

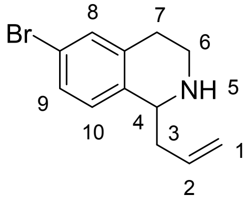

6-Bromo-3,4-dihydroisoquinoline (121 mg, 0.57 mmol) was reacted with boron trifluoride dietherate (1.14 mmol, 0.14 mL) and allyl magnesium bromide (0.5M in Et_2O, 1.14 mL, 0.57 mmol) at r.t under nitrogen. Purification over silica gel (20% v/v methanol:DCM) afforded the desired product (110 mg, 0.44 mmol, 74%) as a dark yellow oil.

^{1}H NMR (400 MHz, $CDCl_3$) δ 7.36 (d, J= 8.4 Hz, 1H, H-9), 7.31 (s, 1H, H-8), 7.06 (d, J= 8.4 Hz, 1H, H-10), 5.73 (m, 2H, H-5,2), 5.29 (m, 2H, H-1), 4.43 (dd, J= 7.6, 4.9 Hz, 1H, H-4), 3.51 (dt, J= 12.8, 5.7 Hz, 1H, H-6), 3.32 (dt, J= 12.8, 7.0, 5.5 Hz, 1H, H-6), 3.00 (dddd, J= 11.5, 10.0, 8.2, 6.6 Hz, 2H, H-7), 2.73 (m, 2H, H-3). **^{13}C NMR** (101 MHz, $CDCl_3$) δ 134.6, 132.1, 131.6, 131.2, 130.2, 128.0, 121.7, 121.6, 54.8, 40.2, 38.8, 26.3.
MS (ESI) (m/z) = 252.0295 (M+H$^+$), calc. 252.0310.

1-Allyl-6-chloro-1,3,4-tetrahydroisoquinoline, (*rac*)-3c

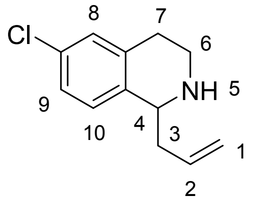

6-Chloro-3,4-dihydroisoquinoline (119 mg, 0.71 mmol) was reacted with boron trifluoride dietherate (1.42 mmol, 0.17 mL) and allyl magnesium bromide (0.5M in Et_2O, 1.42 mL, 0.71 mmol) at r.t under nitrogen. Purification over silica gel (20% v/v methanol:DCM) afforded the desired product (94 mg, 0.45 mmol, 66%) as a yellow oil.

^{1}H NMR (400 MHz, $CDCl_3$) 7.21 (d, *J*= 8.4 Hz, 1H, H-9), 7.15 (s, 1H, H-8), 7.12 (d, *J*= 8.4, 1H, H-10), 5.72 (m, 1H, H-2), 5.60 (br. s, 1H, H-5), 5.28 (m, 2H, H-1), 4.55 (dd, *J*= 7.5, 5.1 Hz, 1H, H-4), 3.55 (dt, *J*= 12.5, 6.7 Hz , 1H, H-6), 3.39 (dt, *J*= 13.3, 6.7 Hz, 1H, H-6), 3.02 (m, 2H, H-7), 2.74 (m, 2H, H-3). **^{13}C NMR** (101 MHz, $CDCl_3$) 133.9, 133.6, 130.6, 129.8, 129.0, 127.9, 127.5, 122.1, 54.7, 40.1, 38.4, 25.5.
MS (ESI) (m/z) = 208.0899 (M+H+), calc. 208.0815

1-Allyl-6-fluoro-1,3,4-tetrahydroisoquinoline, (*rac*)-3d

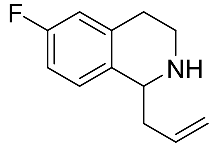

6-Fluoro-3,4-dihydroisoquinoline (127 mg, 0.85 mmol) was reacted with boron trifluoride dietherate (1.70 mmol, 0.21 mL) and allyl magnesium bromide (0.5 M in Et_2O, 1.70 mL, 0.85 mmol) at r.t under nitrogen. Purification over silica gel (20% v/v methanol:DCM) afforded the desired product (48 mg, 0.25 mmol, 30%) as a dark orange oil.

^{1}H NMR (400 MHz, $CDCl_3$) δ7.16 (d, *J*= 8.6 Hz, 1H, ArCH), 7.06 (s, 1H, ArCH), 6.96 (d, *J*= 8.6 Hz, 1H, ArCH), 5.78 (dddd, *J*= 15.9, 10.0, 7.5, 6.4 Hz, 1H, CH=), 5.18 (m, 2H, =CH_2), 4.56 (dd, *J*= 7.4, 4.6 Hz, 1H, CH), 3.28 (m, 1H, CH_2), 3.19 (m, 1H, CH_2), 2.71 (m, 2H, CH_2), 2.62 (m, 2H, CH_2), NH not observed. Data consistent with the literature.[11]
MS (ESI) (m/z) = 192.1187 (M+H^+), calc. 192.1110.

1-Allyl-6-methoxy-1,3,4-tetrahydroisoquinoline, (*rac*)-3e

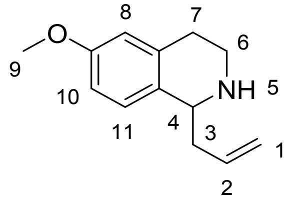

6-Methoxy-3,4-dihydroisoquinoline (131 mg, 0.81 mmol) was reacted with boron trifluoride dietherate (1.62 mmol, 0.20 mL) and allyl magnesium bromide (0.5M in Et_2O, 1.62 mL, 0.81 mmol) at r.t under nitrogen. Purification over silica gel (20% v/v methanol:DCM) afforded the desired product (108 mg, 0.53 mmol, 66%) as a brown oil.

^{1}H NMR (400 MHz, $CDCl_3$) δ 7.10 (d, *J*= 8.7 Hz, 1H, H-11), 6.80 (app. dd, *J*= 8.7, 2.6 Hz, 1H, H-10), 6.66 (app. d, J= 2.6 Hz, 1H, H-8), 6.02 (br. s, 1H, H-5), 5.74 (m, 1H, H-2), 5.32 (m, 2H, H-1), 4.57 (dd, *J*= 7.4, 4.8 Hz, 1H, H-4), 3.79 (s, 3H, H-9), 3.63 (m, 1H, H-6), 3.43 (m, 1H, H-6), 3.11 (m, 1H, H-7), 3.00 (m, 1H, H-7), 2.80 (m, 2H, H-3) **^{13}C NMR** (101 MHz, $CDCl_3$) δ 159.2, 132.9, 130.5, 127.5, 122.8, 122.3, 114.0, 113.5, 55.3, 55.1, 40.8, 38.4, 26.0.
MS (ESI) (m/z) = 204.1393 ($M+H^+$), calc. 204.1310.

5-Allyl-5,6,7,8-[1,3]dioxolo[4,5-g]isoquinoline, (*rac*)-3h

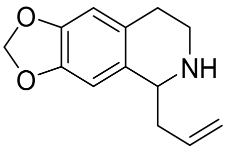

7,8-Dihydro-[1,3]dioxolo[4,5-g]isoquinoline (97 mg, 0.55 mmol) was reacted with boron trifluoride dietherate (1.10 mmol, 0.14 mL) and allyl magnesium bromide (0.5M in Et_2O, 1.10 mL, 0.55 mmol) at r.t under nitrogen. Purification over silica gel (20% v/v methanol:DCM) afforded the desired product (28 mg, 0.13 mmol, 24%) as an orange oil.

^{1}H NMR (400 MHz, $CDCl_3$) δ 6.63 (s, 1H, ArCH), 6.54 (s, 1H, ArCH), 5.88 (s, 2H, CH_2), 5.80 (dddd, *J*= 16.9, 10.1, 9.2, 7.4 Hz, 1H, =CH), 5.16 (m, 2H, =CH_2), 3.94 (dd, *J*= 8.8, 3.7 Hz, 1H, CH), 3.18-3,41 (m, 2H, CH_2), 2.91 (ddd, *J*= 12.6, 7.8, 5.1 Hz, 1H, CH_2), 2.70 (m, 2H, CH_2), 2.47 (m, 1H, CH_2). Data consistent with the literature.[10]
MS (ESI) (m/z) = 218.1185 ($M+H^+$), calc. 218.1103.

1-Allyl-8-methyl-1,3,4-tetrahydroisoquinoline, (*rac*)-3i

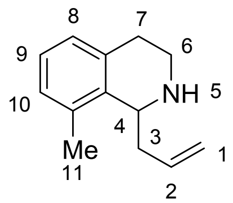

8-Methyl-3,4-dihydroisoquinoline (150 mg, 1.03 mmol) was reacted with boron trifluoride dietherate (2.06 mmol, 0.25 mL) and allyl magnesium bromide (0.5M in Et_2O, 2.06 mL, 1.03 mmol) at r.t under nitrogen. Purification over silica gel (20% v/v methanol:DCM) afforded the desired product (71 mg, 0.38 mmol, 38%) as an orange oil.

^{1}H NMR (400 MHz, $CDCl_3$) δ 7.17 (app. t, *J*= 7.6 Hz, 1H, H-9), 7.03 (app. dd, *J*= 10.6, 7.6 Hz, 2H, H-8,10), 6.35 (br. s, 1H, H-5), 5.78 (dddd, *J*= 16.6, 10.1, 6.0, 4.1 Hz, 1H, H-2), 5.33 (m, 2H, H-1), 4.79 (dd, *J*= 9.6, 4.1 Hz, 1H, H-4), 3.54 (m, 2H, H-6), 3.17 (m, 1H, H-7), 2.99 (m, 1H, H-7), 2.69 (m, 1H, H-3), 2.57 (m, 1H, H-3), 2.29 (s, 3H, H-11). **^{13}C NMR** (101 MHz, $CDCl_3$) δ 134.9, 130.8, 130.7, 129.7, 129.6, 128.2, 127.0, 122.0, 52.7, 37.8, 36.9, 25.2, 18.8.
MS (ESI) (m/z) = 187.1445($M+H^+$), calc. 187.1361.

1-Allyl-8-bromo-1,3,4-tetrahydroisoquinoline, (*rac*)-3j

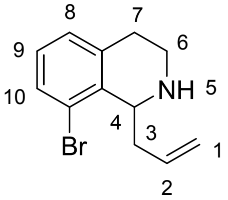

8-Bromo-3,4-dihydroisoquinoline (168 mg, 0.80 mmol) was reacted with boron trifluoride dietherate (1.6 mmol, 0.25 mL) and allyl magnesium bromide (0.5M in Et_2O, 1.60 mL, 0.80 mmol) at r.t under nitrogen. Purification over silica gel (20% v/v methanol:DCM) afforded the desired product (143 mg, 0.57 mmol, 71%) as an dark orange oil.

^{1}H NMR (400 MHz, $CDCl_3$) δ 7.39 (d, *J*= 8.5 Hz, 1H, H-10), 7.05 (m, 2H, H-8,9), 5.93 (dddd, *J*= 17.9, 11.8, 9.0, 2.9 Hz, 1H, H-2), 5.21 (m, 2H, H-1), 4.26 (dd, *J*= 11.8, 2.9 Hz, 1H, H-4), 3.25 (m, 1H, H-6), 3.10 (m, 1H, H-6), 2.98 (m, 1H, H-7), 2.74 (m, 2H, H-3), 2.46 (m, 1H, H-7), 1.25 (br. s, 1H, H-5). **^{13}C NMR** (101 MHz, $CDCl_3$) δ 136.9, 136.8, 135.2, 130.9, 128.6, 127.9, 123.0, 118.3, 54.8, 37.0, 36.8, 28.5
MS (ESI) (m/z) = 152.0388 ($M+H^+$), calc. 252.0310.

1-Allyl-2,3,4,9-tetrahydro-1H-pyrido[3,4-b]indole, (*rac*)-3k

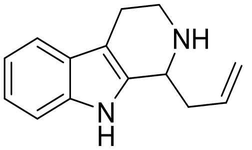

4,9-Dihydro-3H-pyrido[3,4-b]indole (158 mg, 0.90 mmol) was reacted with boron trifluoride dietherate (1.80 mmol, 0.22 mL) and allyl magnesium bromide (0.5M in Et_2O, 1.80 mL, 0.90 mmol) at r.t under nitrogen. Purification over silica gel (20% v/v methanol:DCM) afforded the desired product (120 mg, 0.57 mmol, 62%) as yellow oil.

^{1}H NMR (400 MHz, $CDCl_3$) δ 7.92 (s, 1H, NH), 7.49 (d, *J*= 7.8 Hz, 1H, ArCH), 7.32 (d, *J*= 7.8 Hz, 1H, ArCH), 7.13 (m, 2H, ArCH), 5.95 (dddd, *J*= 17.4, 10.3, 9.2, 7.0 Hz, 1H, CH=), 2.58 (m, 2H, $=CH_2$), 4.18 (dd, *J*= 10.3, 2.9 Hz, 1H, CH), 4.18 (m, 1H, CH_2), 3.05 (m, 1H, CH_2), 2.75 (m, 2H, CH_2), 2.58 (m, 2H, CH_2), 1.85 (bs, 1H, NH). Data consistent with the literature.[10]
MS (ESI) (m/z) = 213.1394 ($M+H^+$), calc. 213.1313.

3.4. Alternative method for the preparation of 1-allyl tetrahydroisoquinolines

1-Allyl-6,7-dimethoxy-1,3,4-tetrahydroisoquinoline, (*rac*)-3g

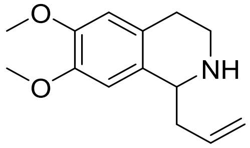

$FeCl_2$ (4.4 mg, 0.03 mmol) and 1,2-bis(diphenylphosphino)benzene (15.2 mg, 0.03 mmol) were stirred in anhydrous THF (4 mL) for 30 minutes at r.t under N_2. After this time methanol (0.4 mL, 6.5 mmol), 6,7-dimethoxy-3,4-dihydroisoquinoline (262 mg, 1.30 mmol) and potassium allyltrifluoroborate (222 mg, 1.50 mmol) were added and the reaction stirred at 80 °C overnight. The reaction was cooled to r.t and diluted with DCM. The crude reaction mixture was filtered through celite, before concentrating *in vacuo*. Purification over silica gel (10% v/v methanol:EtOAc) afforded the desired product (62 mg, 0.27 mmol, 21%) as a yellow oil.

^{1}H NMR (400 MHz, $CDCl_3$) δ 6.63 (s, 1H, ArH), 6.58 (s, 1H, ArH), 5.90 (m, 1H, CH=), 5.32-5.18 (m, 2H, $=CH_2$), 4.36 (t, *J*= 6.1 Hz, 1H, CH), 3.84 (s, 3H, CH_3), 3.83 (s, 3H, CH_3), 3.54-3.48 (m, 1H, CH_2), 3.22 (m, 1H, CH_2), 2.98 (m, 2H, CH_2), 2.82 (t, *J*= 6.6 Hz, 2H, CH_2), NH not observed. Data consistent with the literature.[10]
MS (ESI) (m/z) = 234.1493 ($M+H^+$), calc. 234.1416.

3.5. Preparation of allylic Grignards

Mg turnings (1.5 equiv.) were stirred in a two-neck round-bottom flask fitted with a condenser, under N_2 for 15 minutes before addition of dry Et_2O (1.1 mL $mmol^{-1}$) and a single crystal of I_2. Subsequently, allylic bromides (2.0 equiv.) were added dropwise and the reaction initiated with a heat gun. The reaction mixture was left to stir for 30 minutes before allowing to cool and being taken through to the next step without further purification or characterisation.

3.6. Preparation of 1-substituted tetrahydroisoquinolines

The corresponding allylic Grignard (2.0 equiv.) was added dropwise to a cooled (-25 °C) solution of 3,4-dihydroisoquinoline (1.0 equiv.) and $BF_3.OEt_2$ (1.1 equiv.) in dry THF. The reaction was stirred at -25 °C for 2 hours before being allowed to warm to r.t and left to react overnight. After this time, the reaction was quenched by addition of water and left to stir for a further 1 hour. Subsequently, the mixture was extracted with EtOAc (3 x 5 mL), dried over anhydrous $MgSO_4$, filtered and concentrated *in vacuo*.

1-(2-Methylallyl)-1,2,3,4-tetrahydroisoquinoline, (*rac*)-3l

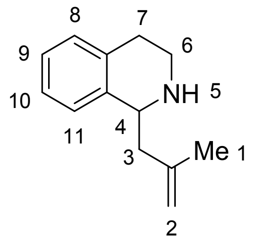

3,4-Dihydroisoquinoline (250 mg, 1.9 mmol) was reacted with $BF_3.OEt_2$ (2.1 mmol, 295 mg) and 2-methylallylmagnesium bromide (1 M in THF, 4.0 mL, 4.0 mmol). Purification over silica gel (10% v/v DCM-methanol) afforded the product (132 mg, 0.70 mmol, 37%) as a dark yellow oil.

^{1}H NMR (400 MHz, $CDCl_3$) δ 7.19 (m, 4H, H-8,9,10,11), 5.05 (br. t, *J*= 1.5 Hz, 1H, H-2), 4.99 (s, 1H, H-2), 4.65 (t, *J*= 7.1 Hz, 1H, H-4), 3.55 (m, 1H, H-7), 3.42 (m, 2H, H-6), 3.12 (m, 1H, H-7), 2.71 (m, 2H, H-3), 1.82 (s, 3H, H-1), NH not observed. **^{13}C NMR** (101 MHz, $CDCl_3$) δ 138.6, 131.3, 131.2, 129.2, 128.2, 127.2, 126.5, 117.5, 53.1, 43.0, 40.0, 25.4, 21.9.
MS (ESI) (m/z) = 188.1437 ($M+H^+$), calc. 188.1361.

1-(2-Phenylallyl)-1,2,3,4-tetrahydroisoquinoline, (*rac*)-3n

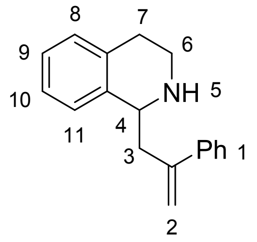

3,4-Dihydroisoquinoline (150 mg, 1.14 mmol) was reacted with $BF_3.OEt_2$ (1.58 mmol, 224 mg) and (2-phenylallyl)magnesium bromide (1 M in THF, 2.0 mL, 2.1 mmol). Purification over silica gel (10% v/v DCM-methanol) afforded the product (79 mg, 0.29 mmol, 28%) as a yellow oil.

^{1}H NMR (400 MHz, $CDCl_3$) δ 7.04-7.53 (m, 9H, H-1,8,9,10,11), 5.52 (s, 1H, H-2), 5.30 (s, 1H, H-2), 4.43 (dd, *J*= 7.9, 5.9 Hz, 1H, H-4), 3.58 (m, 1H, H-7), 3.38 (m, 1H, H-7), 3.21 (m, 2H, H-6), 3.07 (m, 2H, H-3), NH not observed. **^{13}C NMR** (101 MHz, $CDCl_3$) δ 141.7, 138.7, 131.2, 131.0, 129.2, 128.9, 128.4, 128.2, 127.0, 126.7, 126.5, 119.1, 53.7, 40.7, 39.9, 25.4.
MS (ESI) (m/z) = 250.1495 ($M+H^+$), calc. 250.1517.

1-(But-2-en-1-yl)-1,2,3,4-tetrahydroisoquinoline, (*rac*)-3o

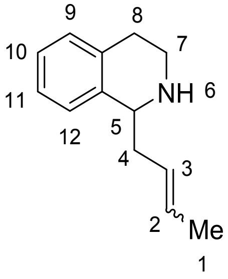

3,4-Dihydroisoquinoline (150 mg, 1.14 mmol) was reacted with $BF_3.OEt_2$ (1.58 mmol, 224 mg) and crotylmagnesium bromide (1 M in THF, 2.0 mL, 2.1 mmol). Purification over silica gel (10% v/v DCM-methanol) afforded the product (42 mg, mmol, 0.22 mmol, 20%) as a light yellow oil.

Major stereoisomer: **^{1}H NMR** (400 MHz, $CDCl_3$) δ 7.14-7.36 (m, 4H, H-9,10,11,12), 5.47 (m, 2H, H-2,3), 4.62 (dt, *J*= 8.3, 4.2, 1H, H-5), 3.46 (m, 2H, H-8), 3.12 (m, 2H, H-4), 2.10 (br. s, 1H, H-6), 1.72 (m, 2H, H-7), 1.10 (d, *J*= 7.1, 3H, H-1). **^{13}C NMR** (101 MHz, $CDCl_3$) δ 136.5, 132.4, 129.1, 129.0, 128.1, 128.0, 127.2, 126.2, 119.2, 61.8, 33.5, 30.9, 29.0.

Minor stere0isomer: **^{1}H NMR** (400 MHz, $CDCl_3$) δ 7.14-7.36 (m, 4H, H-9,10,11,12), 5.47 (m, 2H, H-2,3), 4.70 (dt, *J*= 8.6, 4.5, 1H, H-5), 3.69 (m, 2H, H-8), 3.12 (m, 3H, H-4), 2.10 (br. s, 1H, H-6), 1.96 (m, 2H, H-7), 1.33 (d, *J*= 7.0, 3H, H-1). **^{13}C NMR** (101 MHz, $CDCl_3$) δ 134.1, 129.0, 128.2, 127.1, 126.0, 118.7, 93.7, 77.2, 76.8, 76.5, 61.2, 59.4, 25.3, 18.4, 15.7, 13.0.
MS (ESI) (m/z) = 250.1495 ($M+H^+$), calc. 250.1517

1-(But-3-en-2-yl)-1,2,3,4-tetrahydroisoquinoline, (*rac*)-3p

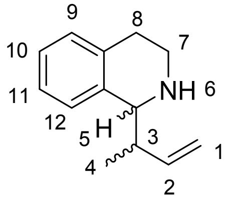

3,4-Dihydroisoquinoline (200 mg, 1.53 mmol) was reacted with $BF_3.OEt_2$ (1.63 mmol, 231 mg) and but-1-ene-3-magnesium chloride (0.5 M in THF, 6 mL, 3.06 mmol). Purification over silica gel (50% v/v EtOAc-pet ether) afforded the product (154 mg, 0.82 mmol, 54%) as an inseparable mixture of diastereomers (1:3) and as a yellow oil.

Peaks for major diastereomer: **^{1}H NMR** (400 MHz, $CDCl_3$) δ 7.04-7.24 (m, 4H, H-9,10,11,12), 6.04 (m, 1H, H-2), 5.18 (m, 2H, H-1), 4.14 (d, *J*= 3.6 Hz, 1H, H-5), 3.27 (m, 1H, H-3), 2.62-2.98 (m, 4H, H-7,8), 1.65 (br. s, 1H, H-6), 0.88 (d, *J*= 6.9 Hz, 3H, H-4). **^{13}C NMR** (101 MHz, $CDCl_3$, diastereomer ratio 1 : 3) δ 142.3, 139.7, 137.0, 129.3, 125.8, 125.8, 125.7, 114.9, 59.8, 42.8, 41.3, 30.5, 11.7.

Peaks for minor diastereomer: **^{1}H NMR** (400 MHz, $CDCl_3$) δ 7.04-7.24 (m, 4H, H-9,10,11,12), 5.69 (m, 1H, H-2), 5.04 (m, 2H, H-1), 4.01 (d, *J*= 4.3 Hz, 1H, H-5), 3.27 (m, 1H, H-3), 2.62-2.98 (m, 4H, H-7,8), 1.65 (br. s, 1H, H-6), 1.25 (d, *J*= 6.9 Hz, 3H, H-4). **^{13}C NMR** (101 MHz, $CDCl_3$, diastereomer ratio 1 : 3) δ 142.3, 139.7, 138.1, 136.3, 129.3, 126.3, 125.9, 115.4, 60.2, 42.0, 41.4, 30.3, 16.5.

MS (ESI) (m/z) = 187.1298 ($M+H^+$), calc. 187.1317.

3.7. Alternative method for the preparation of 1-substituted tetrahydroisoquinolines

1-(2-Methylenebutyl)-1,2,3,4-tetrahydroisoquinoline, (*rac*)-3m

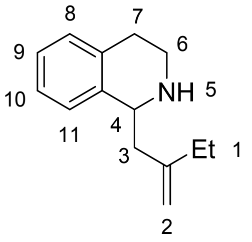

3,4-Dihydroisoquinoline (100 mg, 0.76 mmol, 1.0 equiv.) was added to a 50 mL falcon tube along with 40 mL 100 mM KPi pH 7.8. To the solution was added 10 mol% $Yb(OTf)_3$ (47 mg, 0.076 mmol, 10 mol%) along with 4,4,5,5-tetramethyl-2-(2-methylenebutyl)-1,3,2-dioxaborolane (420 mg, 6.08 mmol, 8.0 equiv.) and the mixture shaken at 30 °C in an incubator overnight. After this time the mixture was basified with 10 M NaOH before extracting with MTBE (3 x 40 mL). The combined organic layers were dried over anhydrous $MgSO_4$, filtered and concentrated *in vacuo*. Purification over silica gel (20% v/v methanol:DCM) afforded the desired product (19 mg, 0.94 mmol, 18%) as an orange oil.

^{1}H NMR (400 MHz, CDCl3) δ 7.17 (m, 4H, H-8,9,10,11), 5.01 (d, *J* = 1.8 Hz, 1H, H-2), 4.96 (s, 1H, H-2), 4.31 (dd, *J*= 9.3, 4.8, 1H, H-4), 3.65 (br. s, 1H, H-5), 3.36 (m, 1H, H-6), 3.14 (m, 1H, H-6), 2.99 (m, 2H, H-1), 2.65 (m, 2H, H-7), 2.15 (m, 2H, H-2), 1.13 (m, 3H, H-1). **^{13}C NMR** (101 MHz, $CDCl_3$) δ 147.7, 137.6, 134.5, 129.2, 126.4, 126.3, 126.0, 111.9, 53.1, 43.0, 40.0, 29.0, 28.4, 12.3
MS (ESI) (m/z) = 202.1597 ($M+H^+$), calc. 202.1517.

1-(Prop-2-yn-1-yl)-1,2,3,4-tetrahydroisoquinoline, (*rac*)-3r

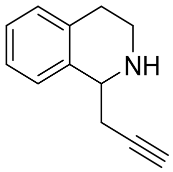

3,4-Dihydroisoquinoline (100 mg, 0.76 mmol, 1.0 equiv.) was reacted with boron trifluoride dietherate (0.12 mL, 0.84 mmol, 1.1 equiv.) and freshly prepared propargyl magnesium bromide (0.5 M in Et_2O, 3 mL, 1.52 mmol, 2.0 equiv.) at r.t under nitrogen. Purification over silica gel (20% v/v methanol:DCM) afforded the desired product (52 mg, 0.30 mmol, 40%) as a light yellow oil.

^{1}H NMR (400 MHz, $CDCl_3$) δ 7.17–7.02 (m, 4H, ArCH), 4.21 (dd, *J*= 8.8, 4.1 Hz, 1H, CH), 3.24–3.20 (m, 1H, CH_2), 3.08–2.92 (m, 1H, CH_2), 2.89–2.76 (m, 2H, CH_2), 2.71 (ddd, *J*= 15.1, 4.2, 2.3 Hz, 1H, CH_2), 2.56 (ddd, *J*= 15.6, 9.4, 3.0 Hz, 1H, CH_2), 2.12 (br s, 1H, NH), 1.98 (t, *J*= 2.6 Hz, 1H, ≡CH). Data consistent with the literature.[12]
MS (ESI) (m/z) = 171.1107 ($M+H^+$), calc. 171.1048.

3.8. Synthesis of substituted allyl boronic acid pinacol esters

4,4,5,5-Tetramethyl-2-(2-methylallyl)-1,3,2-dioxaborolane, 4a

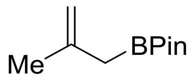

Anhydrous THF (50 mL) was added to a multi-neck flask containing heat activated magnesium turnings (40 mg, 1.65 mmol, 1.2 equiv.), followed by HBPin (0.20 mL, 1.37 mmol, 1.0 equiv.) under an atmosphere of nitrogen at room temperature. 3-Bromo-2-methylprop-1-ene (0.12 mL, 1.37 mmol, 1.0 equiv.) was added dropwise over 5 minutes, followed by bromo-2-methylprop-1-ene (0.12 mL, 1.37 mmol, 1.0 equiv.) after 30 minutes. After 1 hour, the reaction mixture was diluted with hexane and quenched by the slow addition of HCl (5 mL, 0.1 M). The crude compound was extracted with hexanes, dried ($MgSO_4$) and concentrated *in vacuo* to give the titled compound (118 mg, 0.65 mmol, 47%).

^{1}H NMR (400 MHz, $CDCl_3$) δ 4.67 (d, *J*= 6.6 Hz, 2H, =CH_2), 1.77 (s, 3H CH_3), 1.72 (s, 2H CH_2), 1.25 (s, 12H, CH_3). **^{13}C NMR** (101 MHz, $CDCl_3$) δ 143.0, 110.3, 83.4, 25.0, 24.6. Data consistent with the literature.[13]

4,4,5,5-Tetramethyl-2-(2-methylenebutyl)-1,3,2-dioxaborolane, 4b

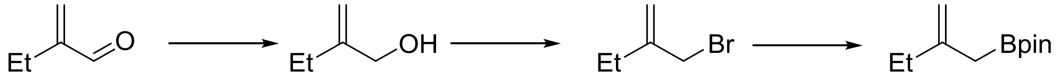

2-Ethylacrolein (2.33 mL, 23.8 mmol, 1.0 equiv.) was dissolved in a 1:8 mixture of MeOH (2 mL) and Et_2O (16 mL) before being cooled to 0 °C. Sodium borohydride (0.88 g, 23.8 mmol, 1.0 equiv.) was added portion-wise. The resulting mixture was stirred at 0 °C for 1 hour before being diluted with Et_2O (20 mL), washed with water (20 mL). The organics were dried ($MgSO_4$) and concentrated *in vacuo* to give the crude product, which was taken to the next step with no further purification.

PBr_3 (3.70 g, 17.8 mmol, 0.75 equiv.) was added dropwise to a solution of the crude alcohol from the above reaction (23.8 mmol, 1.0 equiv.) in dry Et_2O (20 mL) at 0 °C under an atmosphere of nitrogen. Once the addition was complete the reaction mixture was allowed to warm to room temperature and was left to stir for 12 hours. The reaction mixture was cooled to 0 °C before being quenched by the addition of iced water (20 mL). The organic layer was washed successively with water (20 mL), $NaHCO_3$ (20 mL) and brine (20 mL). Due to the volatility of the compound the reaction was concentrated to ¼ of the volume and used in the next step as a solution in Et_2O.

Anhydrous THF (40 mL) was added to a multi-neck flask containing heat activated magnesium turnings (347 mg, 14.3 mmol, 1.2 equiv.), followed by HBPin (1.5 mL, 11.89 mmol, 1.0 equiv.). Half the crude bromide solution prepared as described above was added to the mixture dropwise at room temperature under an atmosphere of nitrogen. After stirring for 30 minutes, the remainder of the bromide solution was added dropwise to the reaction mixture. After stirring at room temperature for 12 hours, the reaction mixture was diluted with hexane and quenched by the slow addition of HCl (40 mL, 0.1 M). The crude compound was extracted into hexanes, dried ($MgSO_4$) and concentrated *in vacuo*. NMR and GCMS data showed the reaction to be pure and therefore no purification required to give the desired compound as a pale yellow oil (822 mg, 4.19 mmol, 18% (over 3 steps)).

^{1}H NMR (400 MHz, $CDCl_3$) δ 4.73 – 4.68 (2H, m, =CH_2), 2.20 – 2.00 (3H, m, CH_2), 1.88 – 1.68 (1H, m, CH_2), 1.24 (12H, s, CH_3), 1.03 (3H, app. td, *J* = 7.4, 2.9 Hz, CH_3). **^{13}C NMR** (101 MHz, $CDCl_3$) δ 148.4, 108.1, 83.4, 34.8, 31.7, 24.9, 22.8. Data consistent with the literature.[14]

4,4,5,5-Tetramethyl-2-(2-phenylallyl)-1,3,2-dioxaborolane, 4c

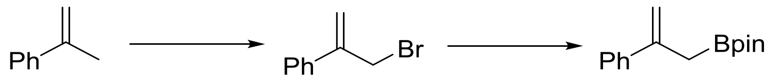

HBPin (0.40 mL, 2.74 mmol, 1.0 equiv.) was added to THF (10 mL) and activated magnesium turnings (82 mg, 3.39 mmol, 1.2 equiv.) in a multi-neck flask under nitrogen atmosphere. 3-Chloro-1-butene (0.28 mL, 2.74 mmol, 1.0 equiv.) was added dropwise, adding half the volume over 5 minutes before waiting 30 minutes before the addition of the remainder of the 3-chloro-1-butene. The reaction mixture was left to stir at room temperature for 12 hours before being diluted with hexanes (10 mL) and quenched by the slow addition of HCl (10 mL, 0.1 M). The organic layer was dried ($MgSO_4$) and concentrated *in vacuo* to give the product (456 mg, 2.50 mmol, 91%) as a colourless oil.

^{1}H NMR (400 MHz, $CDCl_3$) 7.55 – 7.43 (2H, m, ArH), 7.39 – 7.19 (3H, m, ArH), 5.37 (1H, s, =CH_2), 5.10 (1H, app. q, J = 1.3 Hz, =CH_2), 2.16 (2H, s, CH_2), 1.17 (12H, s, CH_3). **^{13}C NMR** (101 MHz, $CDCl_3$) δ 142.7, 141.2, 128.3, 128.15, 126.1, 112.7, 112.4, 83.2, 24.5.Data consistent with literature.[13]

2-(But-3-en-2-yl)-4,4,5,5-tetramethyl-1,3,2-dioxaborolane, 4d

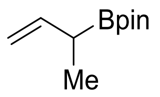

HBPin (0.40 mL, 2.74 mmol, 1.0 equiv.) was added to THF (10mL) and activated magnesium turnings (82 mg, 3.39 mmol, 1.2 equiv.) in a multi-neck flask under nitrogen atmosphere. 3-chloro-1-butene (0.28 mL, 2.74 mmol, 1.0) was added dropwise, adding half the volume over 5 minutes before waiting 30 minutes before the addition of the remainder of the 3-chloro-1-butene. The reaction mixture was left to stir at room temperature for 12 hours before being diluted with hexanes (10 mL) and quenched by the slow addition of HCl (10 mL, 0.1 M). The organic layer was dried ($MgSO_4$) and concentrated *in vacuo* to give the product (456 mg, 2.50 mmol, 91%) as a colourless oil.

^{1}H NMR (400 MHz, $CDCl_3$) δ 5.94 (ddd, J= 17.3, 10.3, 7.1 Hz, 1H, CH), 5.01 – 4.89 (m, 2H, =CH_2), 1.96 – 1.82 (m, 1H, CH), 1.24 (s, 12 H CH_3), 1.10 (d, J= 7.3 Hz, 3H CH_3). **^{13}C NMR** (101 MHz, $CDCl_3$) δ 141.1, 112.1, 83.3, 24.8, 14.2. Data consistent with the literature.[13]

4. Analytical scale optimisation of chemical allylation

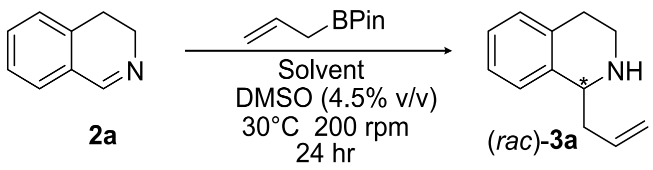

Table S1. Solvent Screen for the Allylboration step

Entry	Solvent	Conversion (%)[a]
1	Toluene	18
2	DCM	57
3	THF	60
4	Methanol	49
5	H_2O	68
6	dH_2O	53
7	100 mM KPi pH 7.8	75
8	100 mM KPi pH 9	62
9	100 mM Tris pH 8	71
10	100 mM Tris pH 9	55

[a] Conversions were based on GCMS data compared to analytic standards.

Table S2. Optimisation of allylBPin equivalence in allylation

Entry	Equivalence of Allyl BPin	Allyl BPin Concentration (mM)	Conversion (%)[a]
1	10	50	75
2	9	45	74
3	8	40	75
4	6	30	70
5	4	20	49
6	2	10	42
7	1.1	5.5	37

4.1. Solvent Screening for the chemical allylation of 3,4-dihydroisoquinoline

To a 2 mL Eppendorf tube was added 5 mM of 3,4-dihydroisoquinoline (0.5 M in DMSO) along with 20 mM Allyl BPin (1 M in DMSO) (See **Table 1**). Solvent was added to give a 500 µL total reaction volume. Biotransformations were incubated at 30 °C for 24 h with 200 rpm shaking. Reactions were quenched by the addition of 40 µL 10M NaOH followed by 500 µL of MTBE before centrifugation at 13,200 rpm for 1.5 minutes. The organic layer was extracted, dried over anhydrous $MgSO_4$ before further centrifugation at 13,200 rpm for 1.5 minutes. The organic product was transferred to a GC vial for analysis by GC/chiral HPLC.

4.2. Optimisation of the chemical allylation of 3,4-dihydroisoquinoline

To a 2 mL Eppendorf tube was added 5 mM of 3,4-dihydroisoquinoline (0.5 M in DMSO) along with Allyl BPin (1 M in DMSO) (See **Table 1**). 100 mM KPi pH 7.8 was added to give a 500 µL total reaction volume. Biotransformations were incubated at 30 °C for 24 h with 200 rpm shaking. Reactions were quenched by the addition of 40 µL 10M NaOH followed by 500 µL of MTBE before centrifugation at 13,200 rpm for 1.5 minutes. The upper organic layer was extracted, dried over anhydrous $MgSO_4$ before further centrifugation at 13,200 rpm for 1.5 minutes. The organic product was transferred to a GC vial for analysis by GC/chiral HPLC.

5. Biotransformations

5.1. Analytical scale procedure for the chemoenzymatic allylation of tetrahydroisoquinoline using whole-cell biocatalyst

To a 2 mL Eppendorf tube was added 5 mM of 1,2,3,4-tetrahydroisoquinoline (1 M in DMSO) along with 40 mM Allyl BPin (1 M in DMSO). 50 mg mL^{-1} of *E. coli.* cells expressing MAO-N (as a 500 mg mL^{-1} stock in 100 mM KPi pH 7.8) was added prior to addition of 100 mM KPi pH 7.8 to give a 500 µL total reaction volume. Biotransformations were incubated at 30 °C for 24 h with 200 rpm shaking. Reactions were quenched by the addition of 40 µL 10M NaOH followed by 500 µL of MTBE before centrifugation at 13,200 rpm for 1.5 minutes. The upper organic layer was extracted, dried over anhydrous $MgSO_4$ before further centrifugation at 13,200 rpm for 1.5 minutes. The organic product was transferred to a GC vial for analysis by GC/chiral HPLC.

5.2. Analytical scale procedure for the chemoenzymatic allylation of tetrahydroisoquinoline using freeze-dried enzyme lysate

To a 2 mL Eppendorf tube was added 5 mM of 1,2,3,4-tetrahydroisoquinoline (1 M in DMSO) along with 40 mM Allyl BPin (1 M in DMSO). 30 mg mL^{-1} of freeze-dried MAO-N lysate (as a 100 mg mL^{-1} stock in 100 mM KPi pH 7.8) was added prior to addition of 100 mM KPi pH 7.8 to give a 500 µL total reaction volume. Biotransformations were incubated at 30 °C for 24 h with 200 rpm shaking. Reactions were quenched by the addition of 40 µL 10M NaOH followed by 500 µL of MTBE before centrifugation at 13,200 rpm for 1.5 minutes. The upper organic layer was extracted, dried over anhydrous $MgSO_4$ before further centrifugation at 13,200 rpm for 1.5 minutes. The organic product was transferred to a GC vial for analysis by GC/chiral HPLC.

5.3. Analytical scale procedure for the chemoenzymatic allylation of tetrahydroisoquinoline using purified enzyme

To a 2 mL Eppendorf tube was added 5 mM of 1,2,3,4-tetrahydroisoquinoline (1 M in DMSO) along with 40 mM Allyl BPin (1 M in DMSO). 2 mg mL^{-1} of purified MAO-N was added prior to addition of 100 mM KPi pH 7.8 to give a 500 µL total reaction volume. Biotransformations were incubated at 30 °C for 24 h with 200 rpm shaking. Reactions were quenched by the addition of 40 µL 10M NaOH followed by 500 µL of MTBE before centrifugation at 13,200 rpm for 1.5 minutes. The upper organic layer was extracted, dried over anhydrous $MgSO_4$ before further centrifugation at 13,200 rpm for 1.5 minutes. The organic product was transferred to a GC vial for analysis by GC/chiral HPLC.

5.4. Analytical scale procedure for the chemoenzymatic allylboration of tetrahydroisoquinoline using purified enzyme biocatalysts and L.A metal catalyst

To a 2 mL Eppendorf tube was added 5 mM of 1,2,3,4-tetrahydroisoquinoline (1 M in DMSO) along with 40 mM Allyl BPin (1 M in DMSO) and 10 mol% Lewis Acid catalysts (100 mM in dH_2O). 2 mg mL^{-1} of purified MAO-N was added prior to addition of 100 mM KPi pH 7.8 to give a 500 µL total reaction volume. Biotransformations were incubated at 30 °C for 24 h with 200 rpm shaking. Reactions were quenched by the addition of 40 µL 10M NaOH followed by 500 µL of MTBE before centrifugation at 13,200 rpm for 1.5 minutes. The upper organic layer was extracted, dried over anhydrous $MgSO_4$ before further centrifugation at 13,200 rpm for 1.5 minutes. The organic product was transferred to a GC vial for analysis by GC/chiral HPLC.

5.5. Analytical scale procedure for the enzymatic deracemisation of (*rac*)-3a using non-selective chemical reducing agents

To a 2 mL Eppendorf tube was added 5 mM of racemic amine substrate, **3a** (1 M in DMSO) along with 50 mM non-selective reducing agent (1 M in DMSO) and 2 mgmL^{-1} purified MAO-N. Finally, 100 mM KPi pH 7.8 was added to give a 500 µL total reaction volume. Biotransformations were incubated at 30 °C for 24 h with 200 rpm shaking. Reactions were quenched by the addition of 40 µL 10 M NaOH followed by 500 µL of MTBE before centrifugation at 13,200 rpm for 1.5 minutes. The upper organic layer was extracted, dried over anhydrous $MgSO_4$ before further centrifugation at 13,200 rpm for 1.5 minutes. The organic product was transferred to a GC vial for analysis by GC/chiral HPLC.

5.6. Analytical scale procedure for the screening of IREDs for the deracemisation of (*rac*)-3a

To a 2 mL Eppendorf tube was added 5 mM of racemic amine substrate, **3a** (1 M in DMSO) along with 40 mM d-glucose (200 mM stock in 100 mM KPi pH 7.8) and 0.4 mM $NADP^+$ (100 mM in 100 mM KPi pH 7.8). 1 mg mL^{-1} of CDX-GDH and 2 mg mL^{-1} of purified MAO-N was added prior to addition of 6 mg mL^{-1} of IRED CFE. Finally, 100 mM KPi pH 7.8 was added to give a 500 µL total reaction volume. Biotransformations were incubated at 30 °C for 24 h with 200 rpm shaking. Reactions were quenched by the addition of 40 µL 10 M NaOH followed by 500 µL of MTBE before centrifugation at 13,200 rpm for 1.5 minutes. The upper organic layer was extracted, dried over anhydrous $MgSO_4$ before further centrifugation at 13,200 rpm for 1.5 minutes. The organic product was transferred to a GC vial for analysis by GC/chiral HPLC.

5.7. Analytical scale procedure for the enantioselective allylation of cyclic amines

To a 2 mL Eppendorf tube was added 5 mM of cyclic amine substrate (1 M in DMSO) along with 40 mM allyl BPin (1 M in DMSO) and 10 mol% Lewis Acid catalyst (100 mM in dH_2O). 40 mM d-glucose (200 mM stock in 100 mM KPi pH 7.8) and 0.4 mM $NADP^+$ (100 mM in 100 mM KPi pH 7.8) were added along with 1 mg mL^{-1} of CDX-GDH and 2 mg mL^{-1} of purified MAO-N D11. Finally, 6 mg mL^{-1} of R-IRED cfe were added and the volume made up to 500 µL with 100 mM KPi pH 7.8. Biotransformations were incubated at 30 °C for 24 h with 200 rpm shaking. Reactions were quenched by the addition of 40 µL 10M NaOH followed by 500 µL of MTBE before centrifugation at 13,200 rpm for 1.5 minutes. The upper organic layer was extracted, dried over anhydrous $MgSO_4$ before further centrifugation at 13,200 rpm for 1.5 minutes. The organic product was transferred to a GC vial for analysis by GC/chiral HPLC.

5.8. Analytical scale procedure for the enantioselective addition of allylic BPin derivatives to cyclic amines

To a 2 mL Eppendorf tube was added 5 mM of 1,2,3,4-tetrahydroisoquinoline (1 M in DMSO) along with 40 mM allylic boryl reagent (1 M in DMSO) and 10 mol% Lewis Acid catalysts (100 mM in dH_2O). 40 mM d-glucose (200 mM stock in 100 mM KPi pH 7.8) and 0.4 mM $NADP^+$ (100 mM in 100 mM KPi pH 7.8) were added along with 1 mg mL^{-1} of CDX-GDH and 2 mg mL^{-1} of purified MAO-N D11. Finally, 6 mg mL^{-1} of R-IRED cfe were added and the volume made up to 500 µL with 100 mM KPi pH 7.8. Biotransformations were incubated at 30 °C for 24 h with 200 rpm shaking. Reactions were quenched by the addition of 40 µL 10M NaOH followed by 500 µL of MTBE before centrifugation at 13,200 rpm for 1.5 minutes. The upper organic layer was extracted, dried over anhydrous $MgSO_4$ before further centrifugation at 13,200 rpm for 1.5 minutes. The organic product was transferred to a GC vial for analysis by GC/chiral HPLC.

6. Chiral HPLC and GC.MS data

6.1. Chiral HPLC: methods and conditions

Standard	Column	*n*-hexane/IPA/diethylamine solvent ratio	Product retention time (min)			
(*rac*)-3a	CHIRALPAK®AD-H	98:2:0.1	8.0 (*S*)		8.6 (*R*)	
(*rac*)-3b	CHIRALPAK®AD-H	95:5:0.1	7.9 (*S*)		8.5 (*R*)	
(*rac*)-3c	CHIRALPAK®AD-H	95:5:0.1	7.4 (*S*)		7.8 (*R*)	
(*rac*)-3d	CHIRALPAK®AD-H	95:5:0.1	7.0 (*S*)		8.5 (*R*)	
(*rac*)-3e	CHIRALPAK®AD-H	98:2:0.1	11.4 (*R*)		12.5 (*S*)	
(*rac*)-3g	CHIRALPAK®OD-H	90:10:0.1	13.6 (*R*)		21.0 (*S*)	
(*rac*)-3h	CHIRALPAK®OD-H	90:10:0.1	7.0 (*R*)		7.8 (*S*)	
(*rac*)-3i	CHIRALPAK®OD-H	95:5:0.1	7.2 (*S*)		8.8 (R)	
(*rac*)-3j	CHIRALPAK®AD-H	90:10:0.1	6.0 (*S*)		7.5 (R)	
(*rac*)-3k	CHIRALPAK®OD-H	90:10:0.1	7.0 (*R*)		7.8 (*S*)	
(*rac*)-3l	CHIRALPAK®OD-H	95:5:0.1	6.3 (*S*)		7.7 (R)	
(*rac*)-3m	CHIRALPAK®OD-H	98:2:0.1	8.0 (*S*)		8.6 (R)	
(*rac*)-3n	CHIRALPAK®AD-H	95:5:0.1	8.1 (*S*)		8.8 (R)	
(*rac*)-3o	CHIRALPAK®AD-H	95:5:0.1	8.3 (R)		10.4 (R)	
(*rac*)-3p	CHIRALPAK®AD-H	95:5:0.1	5.4	5.8	6.3	7.3
(*rac*)-3q	CHIRALPAK®AD-H	98:2:0.1	10.2 (R)		13.4 (S)	

6.2. Absolute configuration

The absolute configurations of optically active products were assigned by comparison with previous reports of the stereoselectivity of the amine oxidase and the imine reductase. All previously reported examples for the oxidation of racemic amines with MAO-N D11 display (*S*)-selective oxidation.[15-17] *R*-IRED is an (*R*)-selective reductase, and many examples have been reported of the *R*-selective reduction of 3,4-dihydroisoquinoline substrates. When coupling these two enzymes in a deracemisation process, a high degree of stereoselectivity will only be observed when the selectivity of both enzymes is complementary, generating (*R*)-configured products. The absolute configuration of (*R*)-**3a** was robustly assigned by optical rotation (and compared to literature), this result was then used to assign the absolute stereochemistry of the other substrates.

6.3. Chiral HPLC and GC.MS data for the MAO-N D11 catalysed kinetic resolution of (*rac*)-3a

The left figures show the GC and corresponding MS trace for the kinetic resolution of (*rac*)-**3a** with MAO-N D11. In the GC.MS data, no imine intermediate is observed. The right figure shows the chiral HPLC trace for the kinetic resolution of (*rac*)-**3a** with MAO-N D11. In the chiral HPLC trace, both enantiomers of (*rac*)-**3a** can be seen, showing slight preference for the *R*-enantiomer through the resolution. The right most peak in the chiral HPLC has been tentatively assigned as the allylic imine intermediate (although we were unsuccessful in chemically synthesising this species). If the area% from the HPLC of the minor (*S*)-enantiomer is summed with the area% of the imine intermediate, this equals the area% of the major (*R*)-enantiomer, which provides good evidence that this is a kinetic resolution process.

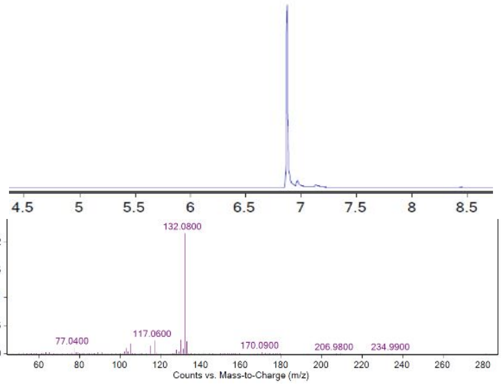

GC-MS (EI) m/z = 173

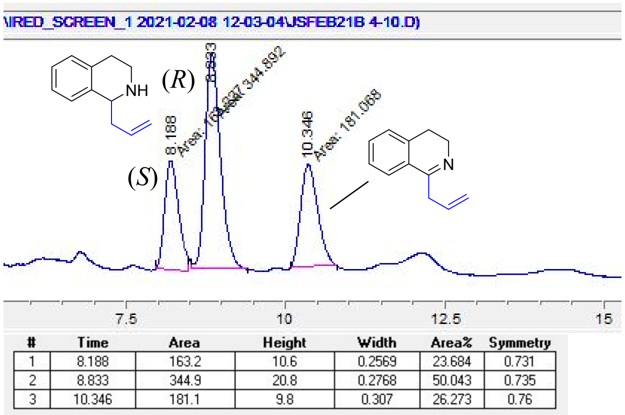

6.4. Chiral HPLC and GC.MS data for analytical biotransformations

(*R*)-1-Allyl-1,2,3,4-tetrahydroisoquinoline, (*R*)-3a

The upper figures show the GC and corresponding MS trace for the imine intermediate **2a** and the racemic product standard (*rac*)-**3a**, along with the chiral HPLC chromatogram for (*rac*)-**3a.** Below is outlined the GCMS and corresponding chiral HPLC chromatogram for the enantioselective chemoenzymatic allylation of **1a** with allyl boronic acid pinacol ester.

GC-MS Data of imine intermediate and racemic product standard

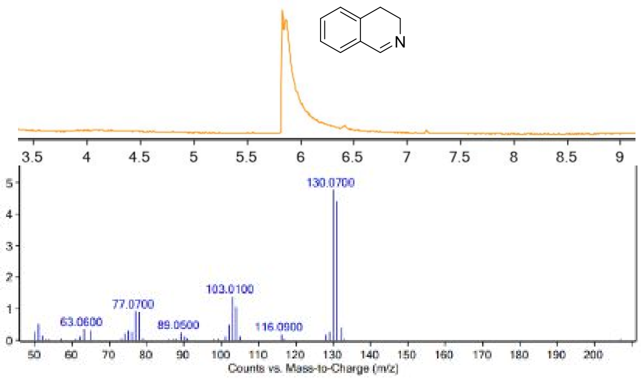

GC-MS (EI) m/z = 131

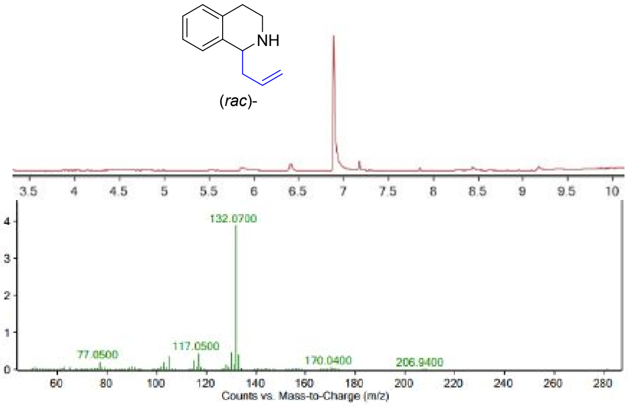

GC-MS (EI) m/z = 173

GC-MS Data from biotransformation

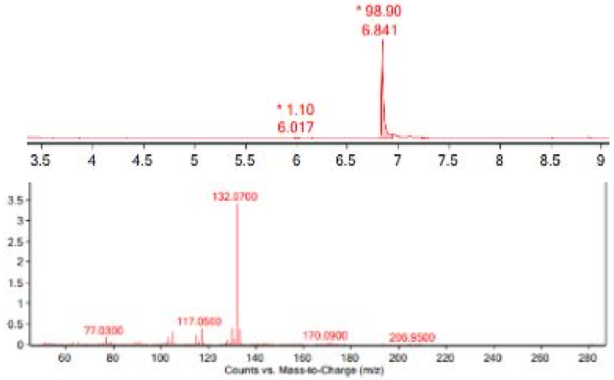

GC-MS (EI) m/z = 173

Chiral HPLC Data

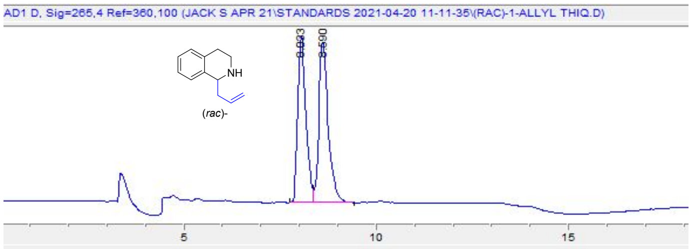

#	Time	Area	Height	Width	Area%	Symmetry
1	8.033	358.6	24.1	0.2255	47.543	0.689
2	8.59	395.7	23.4	0.2515	52.457	0.639

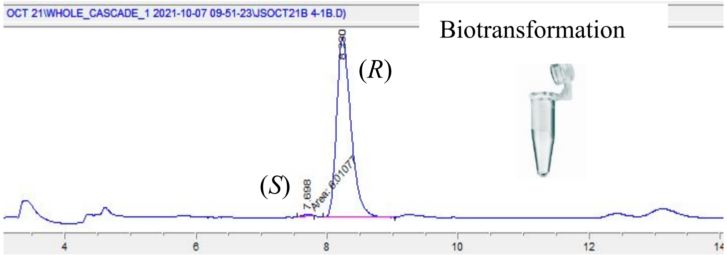

#	Time	Area	Height	Width	Area%	Symmetry
1	7.698	6	6.3E-1	0.1597	0.742	1.067
2	8.23	803.8	52.4	0.235	99.258	0.673

(*R*)-1-Allyl-6-bromo-1,2,3,4-tetrahydroisoquinoline, (*R*)-3b

The upper figures show the GC and corresponding MS trace for the imine intermediate **2b** and the racemic product standard (*rac*)-**3b**, along with the chiral HPLC chromatogram for (*rac*)-**3b.** Below is outlined the GC.MS and corresponding chiral HPLC chromatogram for the enantioselective chemoenzymatic allylation of **1b** with allyl boronic acid pinacol ester.

GC-MS Data of imine intermediate and racemic product standard

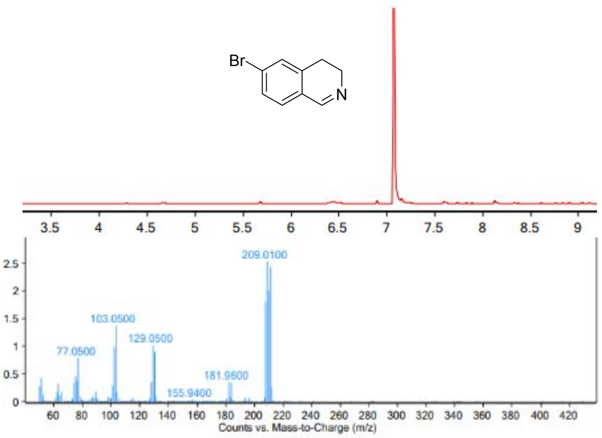

GC-MS (EI) m/z = 209

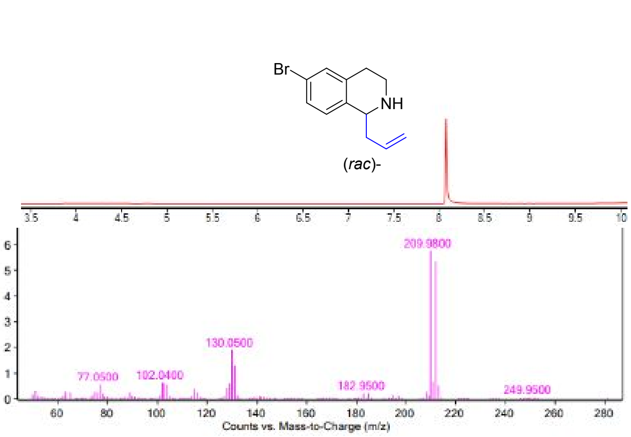

GC-MS (EI) m/z = 251

GC-MS Data from biotransformation

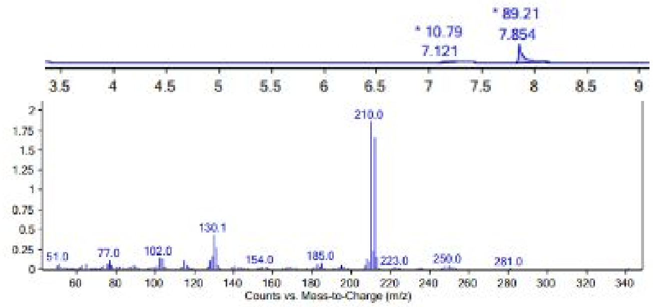

GC-MS (EI) m/z = 251

Chiral HPLC Data

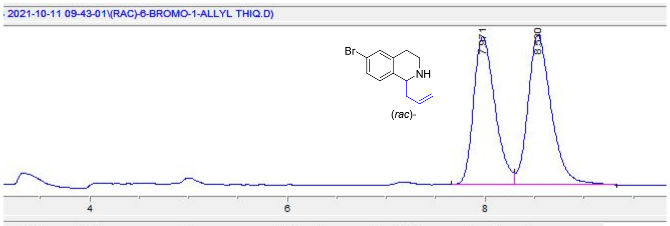

#	Time	Area	Height	Width	Area%	Symmetry
1	7.971	713.9	47.4	0.2317	47.479	0.707
2	8.53	789.7	48	0.252	52.521	0.697

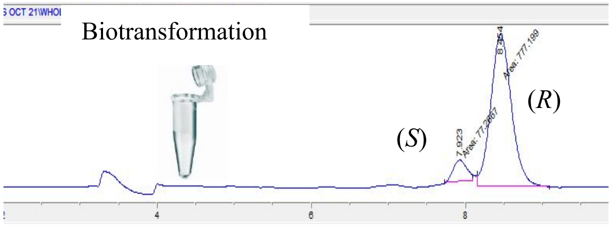

#	Time	Area	Height	Width	Area%	Symmetry
1	7.923	77.3	6.1	0.2106	9.043	0.871
2	8.454	777.2	41.8	0.3096	90.957	0.893

(*R*)-1-Allyl-6-chloro-1,2,3,4-tetrahydroisoquinoline, (*R*)-3c

The upper figures show the GC and corresponding MS trace for the imine intermediate **2c** and the racemic product standard (*rac*)-**3c**, along with the chiral HPLC chromatogram for (*rac*)-**3c.** Below is outlined the GC.MS and corresponding chiral HPLC chromatogram for the enantioselective chemoenzymatic allylation of **1c** with allyl boronic acid pinacol ester.

GC-MS Data of imine intermediate and racemic product standard

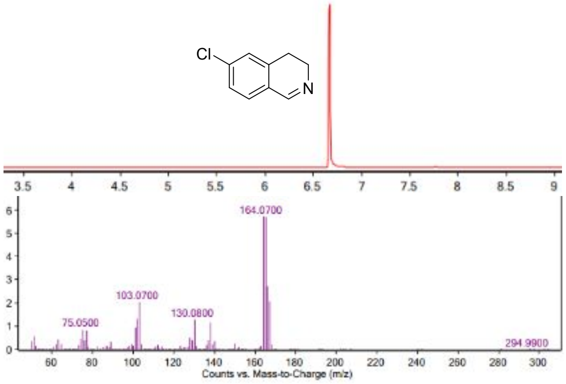

GC-MS (EI) m/z = 165

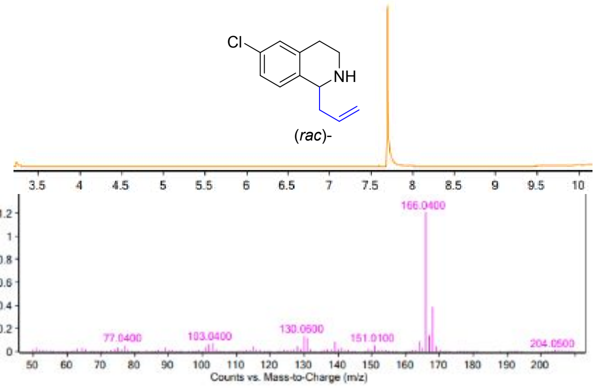

GC-MS (EI) m/z = 207

GC-MS Data from biotransformation

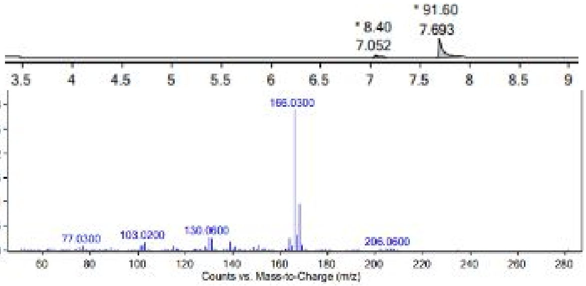

GC-MS (EI) m/z = 207

Chiral HPLC Data

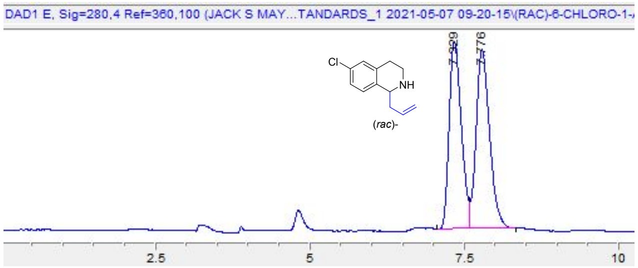

#	Time	Area	Height	Width	Area%	Symmetry
1	7.329	750.6	55.1	0.2089	48.650	0.698
2	7.776	792.3	52.7	0.2295	51.350	0.716

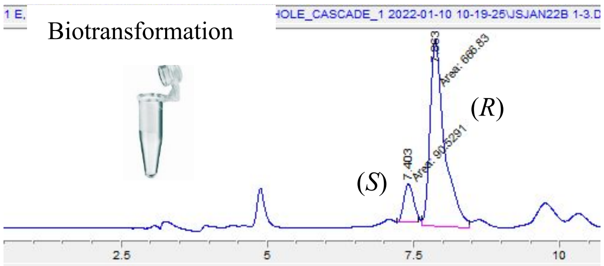

#	Time	Area	Height	Width	Area%	Symmetry
1	7.403	90.5	7.8	0.1934	11.953	0.849
2	7.863	666.8	38.1	0.2914	88.047	0.546

(*R*)-1-Allyl-6-fluoro-1,2,3,4-tetrahydroisoquinoline, (*R*)-3d

The upper figures show the GC and corresponding MS trace for the imine intermediate **2d** and the racemic product standard (*rac*)-**3d**, along with the chiral HPLC chromatogram for (*rac*)-**3d.** Below is outlined the GC.MS and corresponding chiral HPLC chromatogram for the enantioselective chemoenzymatic allylation of **1d** with allyl boronic acid pinacol ester.

GC-MS Data of imine intermediate and racemic product standard

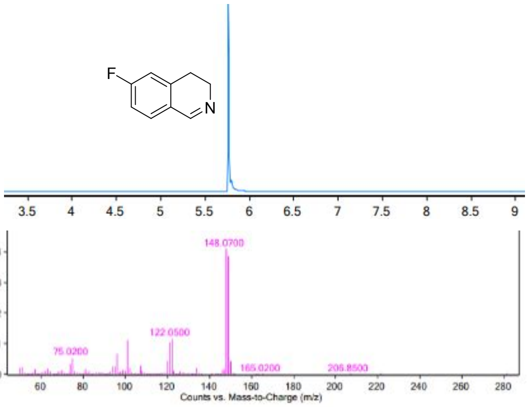

GC-MS (EI) m/z = 149

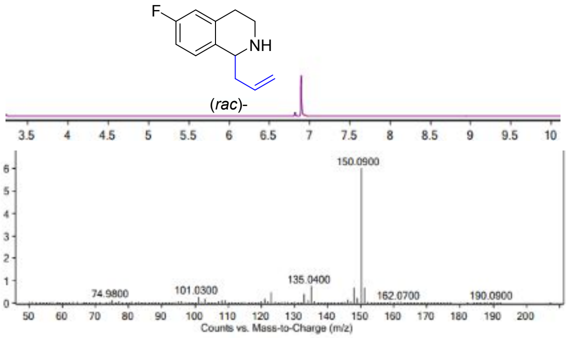

GC-MS (EI) m/z = 191

GC-MS Data from biotransformation

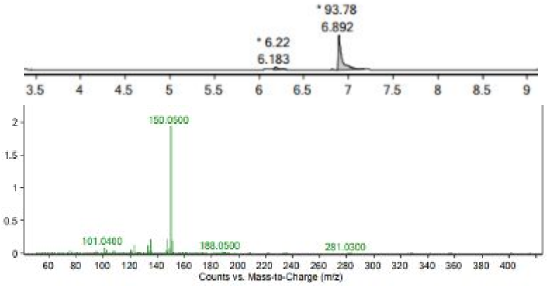

GC-MS (EI) m/z = 191

Chiral HPLC Data

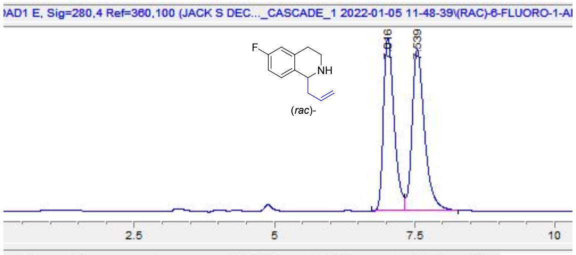

#	Time	Area	Height	Width	Area%	Symmetry
1	7.016	2876.8	216.8	0.2048	48.254	0.681
2	7.539	3084.9	201.6	0.2325	51.746	0.649

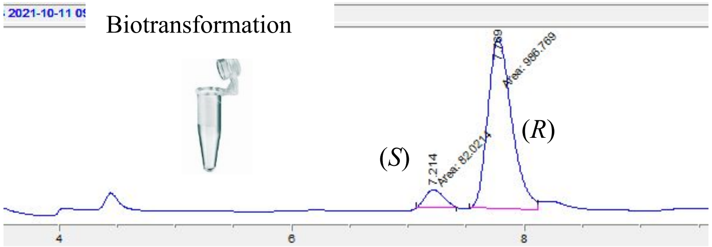

#	Time	Area	Height	Width	Area%	Symmetry
1	7.214	82	7.2	0.1889	7.674	0.766
2	7.769	986.8	68.4	0.2403	92.326	0.721

(*R*)-1-Allyl-6-methoxy-1,2,3,4-tetrahydroisoquinoline, (*R*)-3e

The upper figures show the GC and corresponding MS trace for the imine intermediate **2e** and the racemic product standard (*rac*)-**3e**, along with the chiral HPLC chromatogram for (*rac*)-**3e.** Below is outlined the GC.MS and corresponding chiral HPLC chromatogram for the enantioselective chemoenzymatic allylation of **1e** with allyl boronic acid pinacol ester.

GC-MS Data of imine intermediate and racemic product standard

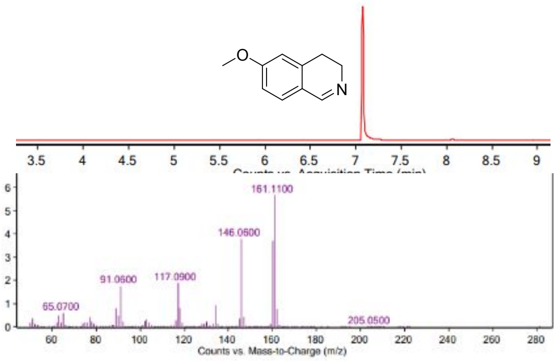

GC-MS (EI) m/z = 161

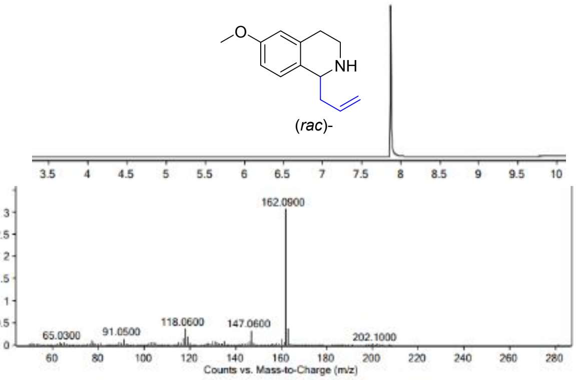

GC-MS (EI) m/z = 203

GC-MS Data from biotransformation

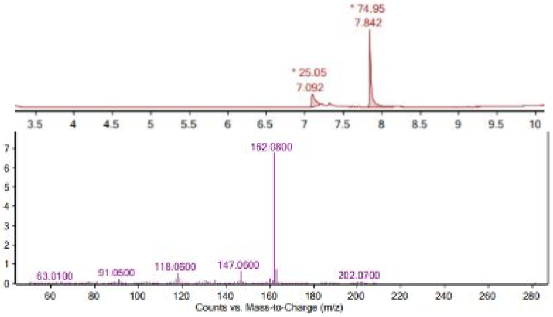

GC-MS (EI) m/z = 203

Chiral HPLC Data

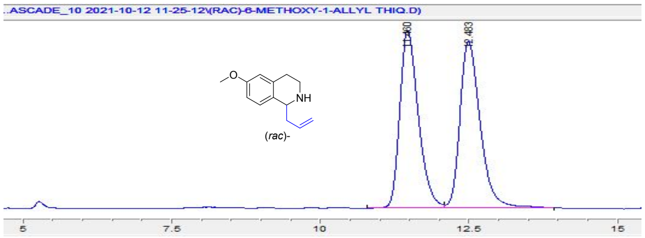

#	Time	Area	Height	Width	Area%	Symmetry
1	11.46	2217.2	101.3	0.3354	49.576	0.708
2	12.483	2255.2	95	0.359	50.424	0.682

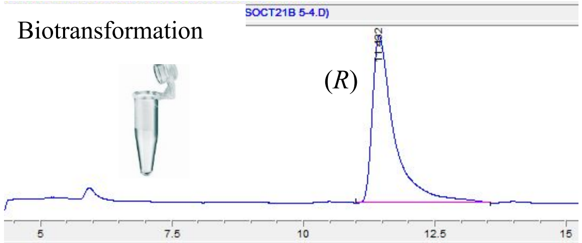

#	Time	Area	Height	Width	Area%	Symmetry
1	11.432	2195.7	74.8	0.4165	100.000	0.396

(*R*)-1-Allyl-6,7-dimethoxy-1,2,3,4-tetrahydroisoquinoline, (*R*)-3g

The upper figures show the GC and corresponding MS trace for the imine intermediate **2g** and the racemic product standard (*rac*)-**3g**, along with the chiral HPLC chromatogram for (*rac*)-**3g.** Below is outlined the GC.MS and corresponding chiral HPLC chromatogram for the enantioselective chemoenzymatic allylation of **1g** with allyl boronic acid pinacol ester.

GC-MS Data of imine intermediate and racemic product standard

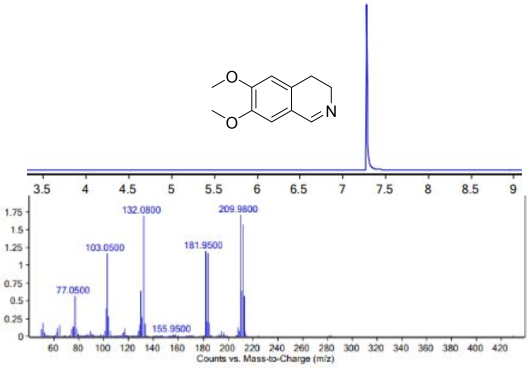

GC-MS (EI) m/z = 191

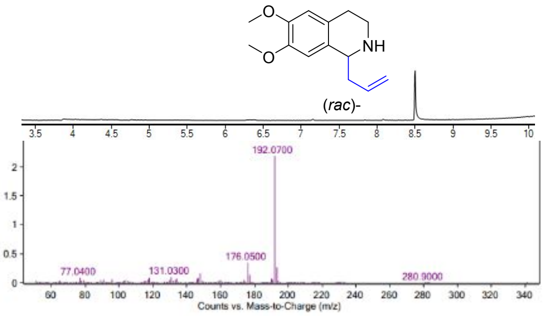

GC-MS (EI) m/z = 233

GC-MS Data from biotransformation

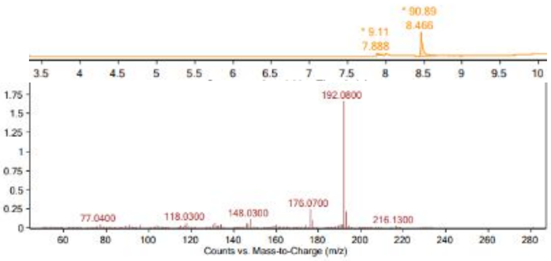

GC-MS (EI) m/z = 233

Chiral HPLC Data

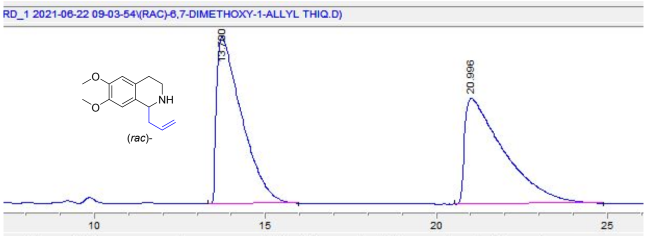

#	Time	Area	Height	Width	Area%	Symmetry
1	13.7	7905.8	154.4	0.6915	50.329	0.237
2	20.996	7802.4	97.8	1.0185	49.671	0.181

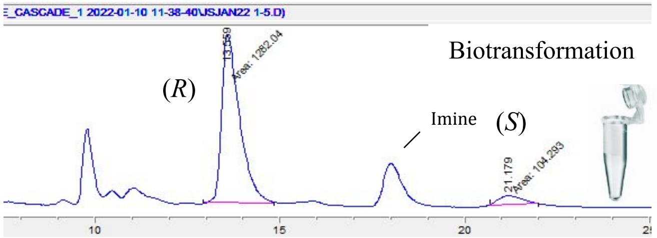

#	Time	Area	Height	Width	Area%	Symmetry
1	13.559	1282	37.8	0.5659	92.477	0.462
2	21.179	104.3	2.2	0.7857	7.523	0.844

(*R*)- 5-Allyl-5,6,7,8-tetrahydro-[1,3]dioxolo[4,5-g]isoquinoline, (*R*)-3h

The upper figures show the GC and corresponding MS trace for the imine intermediate **2h** and the racemic product standard (*rac*)-**3h**, along with the chiral HPLC chromatogram for (*rac*)-**3h.** Below is outlined the GC.MS and corresponding chiral HPLC chromatogram for the enantioselective chemoenzymatic allylation of **1h** with allyl boronic acid pinacol ester.

GC-MS Data of imine intermediate and racemic product standard

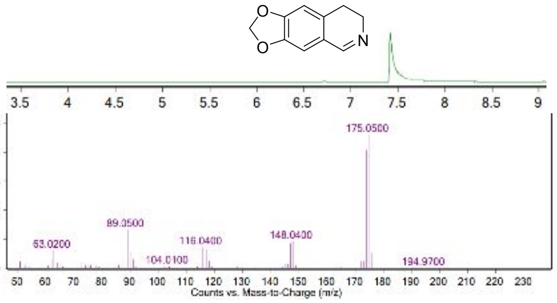

GC-MS (EI) m/z = 175

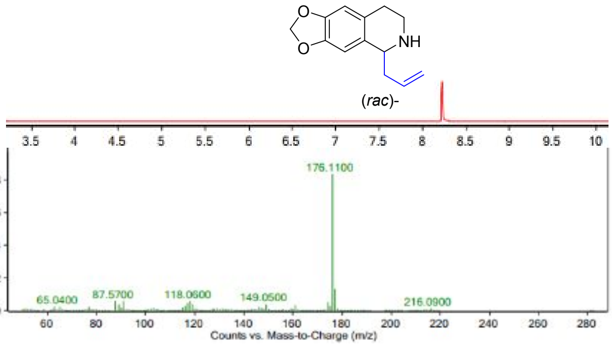

GC-MS (EI) m/z = 217

GC-MS Data from biotransformation

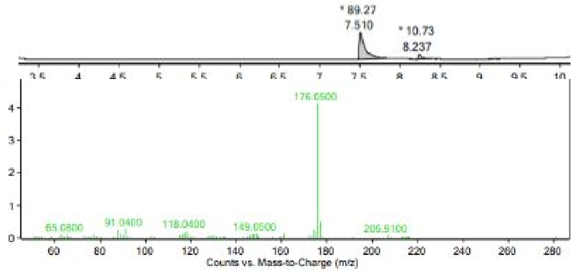

GC-MS (EI) m/z = 217

Chiral HPLC Data

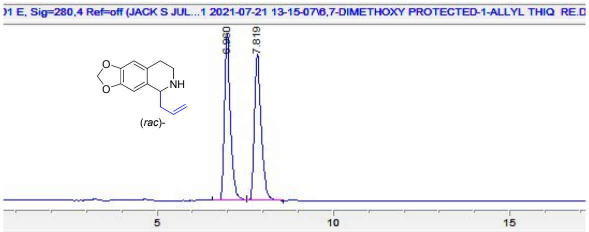

#	Time	Area	Height	Width	Area%	Symmetry
1	6.96	6923	584.2	0.1823	49.890	0.639
2	7.819	6953.6	515.7	0.2053	50.110	0.634

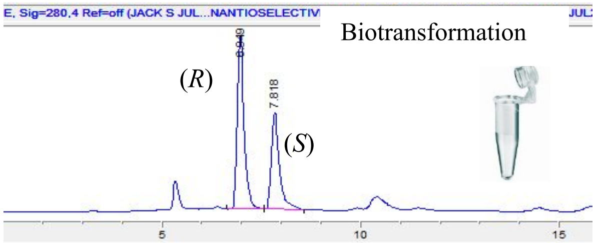

#	Time	Area	Height	Width	Area%	Symmetry
1	6.949	2836.8	236.3	0.182	60.363	0.659
2	7.818	1862.8	132.6	0.2098	39.637	0.612

(*R*)-1-Allyl-8-methyl-1,2,3,4-tetrahydroisoquinoline, (*R*)-3i

The upper figures show the GC and corresponding MS trace for the imine intermediate **2i** and the racemic product standard (*rac*)-**3i**, along with the chiral HPLC chromatogram for (*rac*)-**3i.** Below is outlined the GC.MS and corresponding chiral HPLC chromatogram for the enantioselective chemoenzymatic allylation of **1i** with allyl boronic acid pinacol ester.

GC-MS Data of imine intermediate and racemic product standard

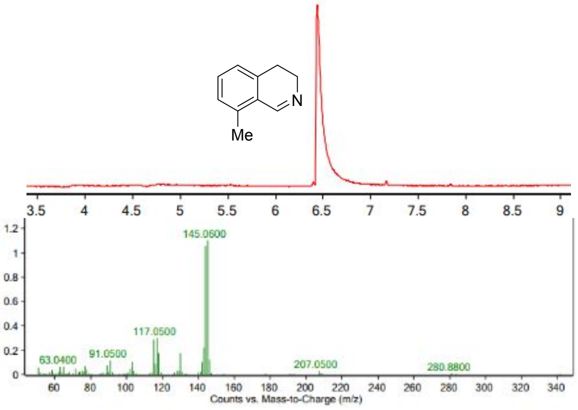

GC-MS (EI) m/z = 145

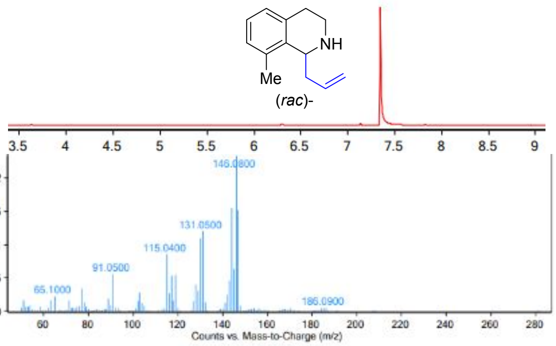

GC-MS (EI) m/z = 187

GC-MS Data from biotransformation

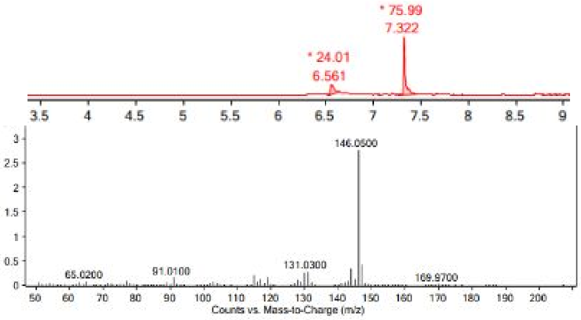

GC-MS (EI) m/z = 187

Chiral HPLC Data

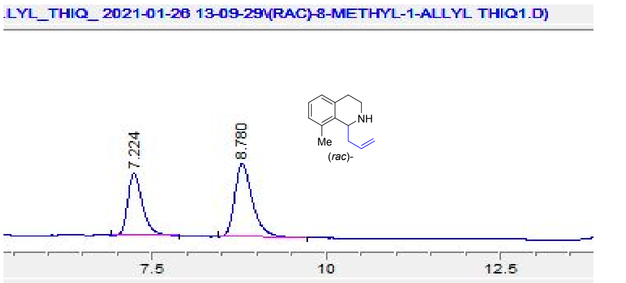

#	Time	Area	Height	Width	Area%	Symmetry
1	7.224	337.5	23	0.2235	41.271	0.674
2	8.78	480.3	27	0.2696	58.729	0.679

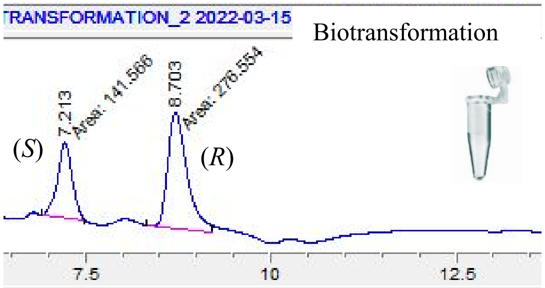

#	Time	Area	Height	Width	Area%	Symmetry
1	7.213	141.6	9.9	0.239	33.858	1.007
2	8.703	276.6	15	0.3082	66.142	0.623

(*R*)-1-Allyl-8-bromo-1,2,3,4-tetrahydroisoquinoline, (*R*)-3j

The upper figures show the GC and corresponding MS trace for the imine intermediate **2k** and the racemic product standard (*rac*)-**3k**, along with the chiral HPLC chromatogram for (*rac*)-**3k.** Below is outlined the GC.MS and corresponding chiral HPLC chromatogram for the enantioselective chemoenzymatic allylation of **1k** with allyl boronic acid pinacol ester.

GC-MS Data of imine intermediate and racemic product standard

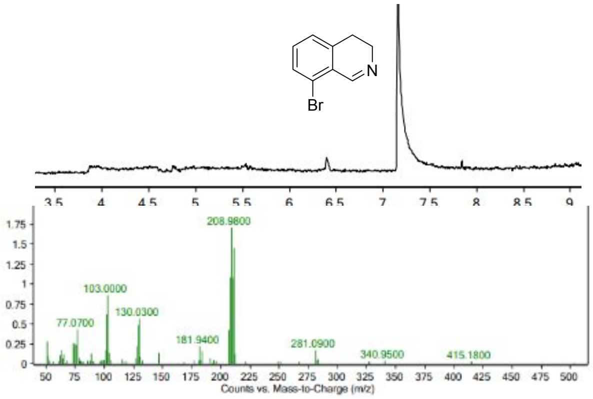

GC-MS (EI) m/z = 208

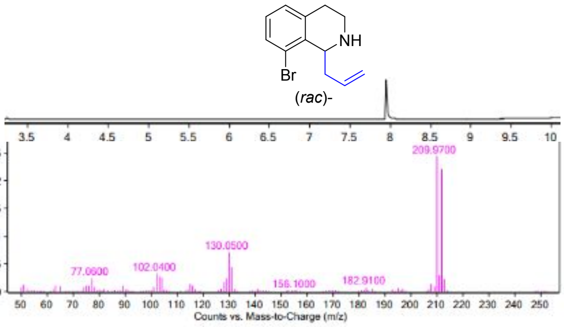

GC-MS (EI) m/z = 251

GC-MS Data from biotransformation

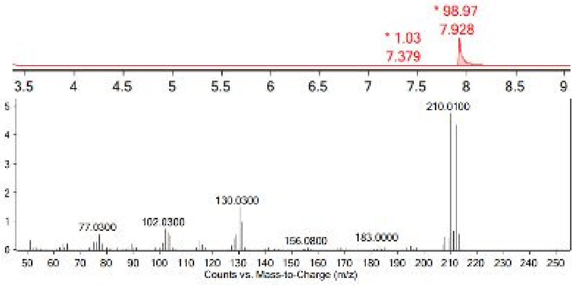

GC-MS (EI) m/z = 251

Chiral HPLC Data

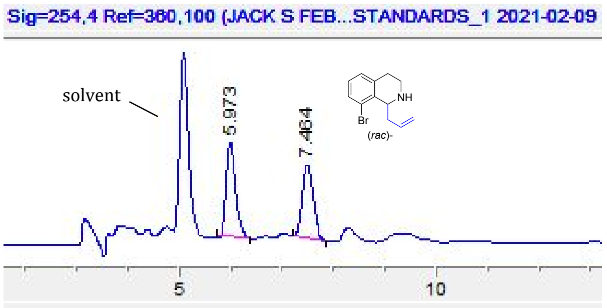

#	Time	Area	Height	Width	Area%	Symmetry
1	5.973	142.6	11.8	0.1891	52.662	0.701
2	7.464	128.2	9.3	0.2191	47.338	0.789

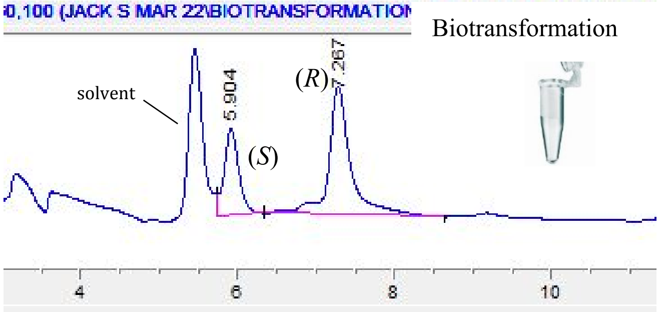

#	Time	Area	Height	Width	Area%	Symmetry
1	5.904	237.5	18.2	0.194	30.827	0.855
2	7.267	533	26.7	0.2833	69.173	0.741

(*R*)- 1-Allyl-2,3,4,9-tetrahydro-1H-pyrido[3,4-b]indole, (*R*)-3k

The upper figures show the GC and corresponding MS trace for the imine intermediate **2l** and the racemic product standard (*rac*)-**3l**, along with the chiral HPLC chromatogram for (*rac*)-**3l.** Below is outlined the GC.MS and corresponding chiral HPLC chromatogram for the enantioselective chemoenzymatic allylation of **1l** with allyl boronic acid pinacol ester.

GC-MS Data of imine intermediate and racemic product standard

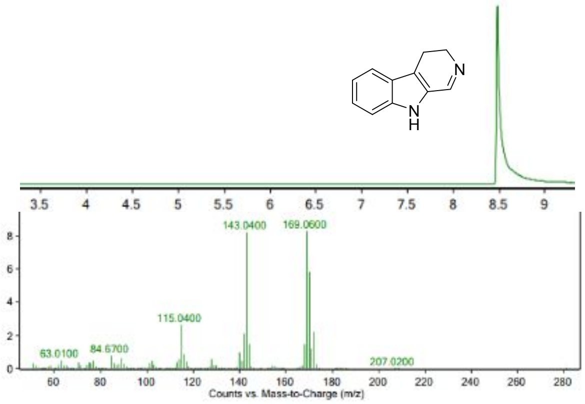

GC-MS (EI) m/z = 170

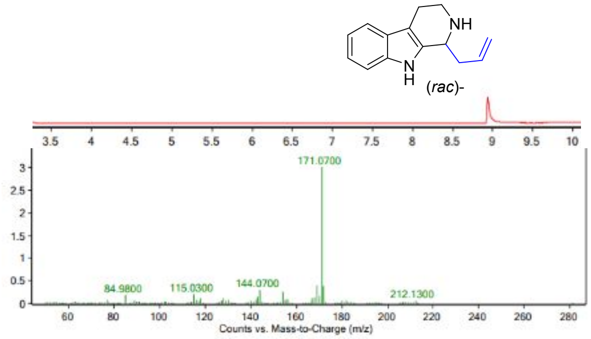

GC-MS (EI) m/z = 212

GC-MS Data from biotransformation

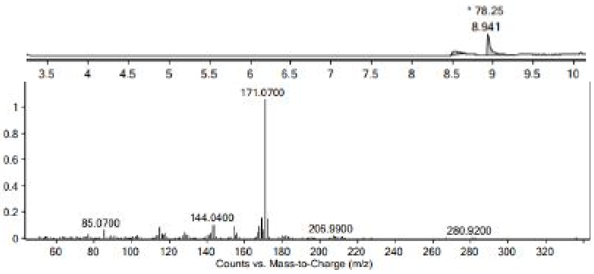

GC-MS (EI) m/z = 212

Chiral HPLC Data

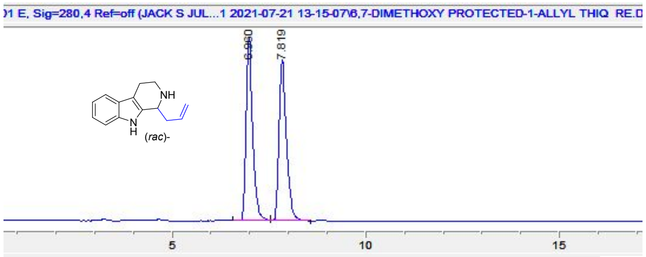

#	Time	Area	Height	Width	Area%	Symmetry
1	8.033	358.6	24.1	0.2255	47.543	0.689
2	8.59	395.7	23.4	0.2515	52.457	0.639

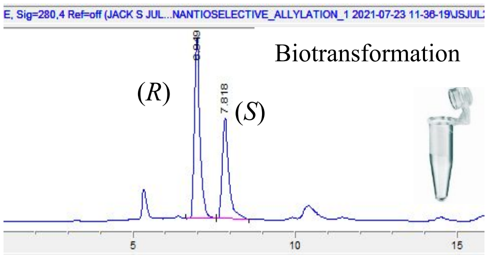

#	Time	Area	Height	Width	Area%	Symmetry
1	6.949	2836.8	236.3	0.182	60.363	0.659
2	7.818	1862.8	132.6	0.2098	39.637	0.612

(*R*)- 1-(2-Methylallyl)-1,2,3,4-tetrahydroisoquinoline, (*R*)-3l

The upper figures show the GC and corresponding MS trace for the imine intermediate **2a** and the racemic product standard (*rac*)-**3o**, along with the chiral HPLC chromatogram for (*rac*)-**3o.** Below is outlined the GC.MS and corresponding chiral HPLC

chromatogram for the enantioselective chemoenzymatic allylation of **1a** with 4,4,5,5-Tetramethyl-2-(2-methylallyl)-1,3,2-dioxaborolane.

GC-MS Data of imine intermediate and racemic product standard

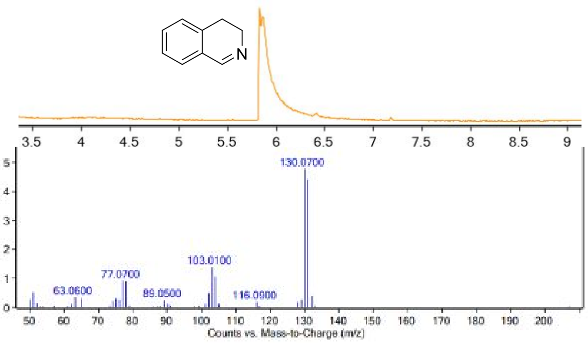

GC-MS (EI) m/z = 131

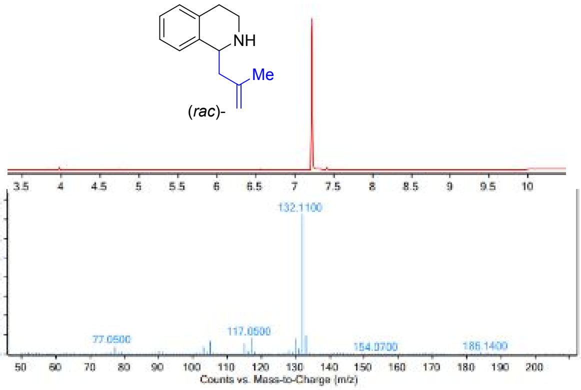

GC-MS (EI) m/z = 187

GC-MS Data from biotransformation

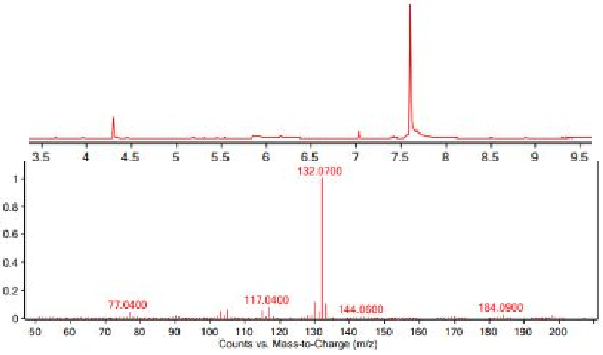

GC-MS (EI) m/z = 187

Chiral HPLC Data

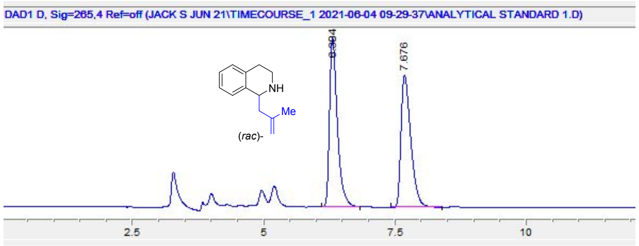

#	Time	Area	Height	Width	Area%	Symmetry
1	6.304	1205.6	117.9	0.1533	50.347	0.571
2	7.676	1189	92.6	0.1938	49.653	0.534

Biotransformation

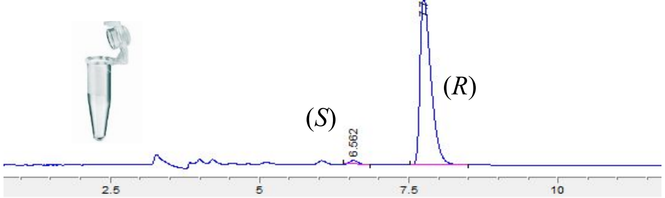

#	Time	Area	Height	Width	Area%	Symmetry
1	6.562	24.8	2.6	0.1419	1.692	0.766
2	7.744	1443.2	113.3	0.1946	98.308	0.504

(*R*)-1-(2-Methylenebutyl)-1,2,3,4-tetrahydroisoquinoline, (*R*)-3m

The upper figures show the GC and corresponding MS trace for the imine intermediate **2a** and the racemic product standard (*rac*)-**3p**, along with the chiral HPLC chromatogram for (*rac*)-**3p.** Below is outlined the GC.MS and corresponding chiral HPLC chromatogram for the enantioselective chemoenzymatic allylation of **1a** with 4,4,5,5-Tetramethyl-2-(2-methylenebutyl)-1,3,2-dioxaborolane.

GC-MS Data of imine intermediate and racemic product standard

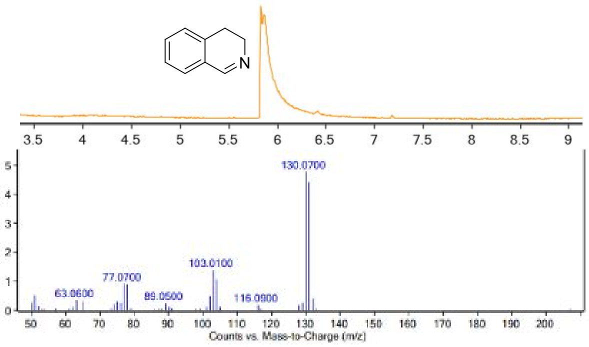

GC-MS (EI) m/z = 131

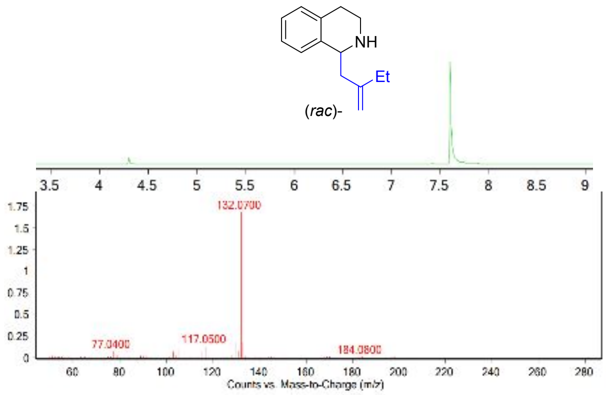

GC-MS (EI) m/z = 201

GC-MS Data from biotransformation

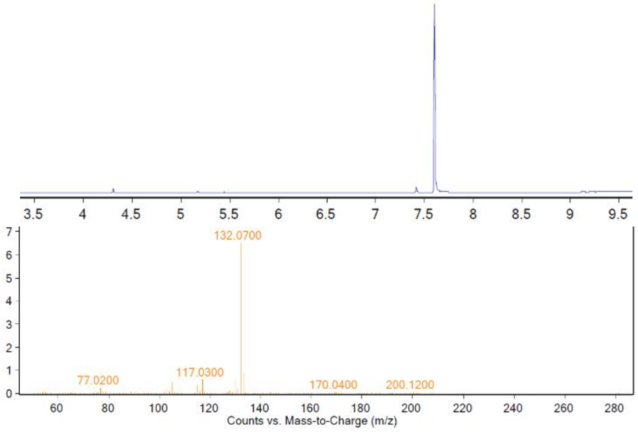

GC-MS (EI) m/z = 201

Chiral HPLC Data

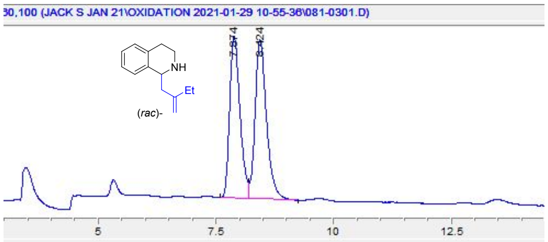

#	Time	Area	Height	Width	Area%	Symmetry
1	7.874	549.7	35.9	0.2366	48.087	0.704
2	8.424	593.5	35.3	0.2562	51.913	0.694

Biotransformation

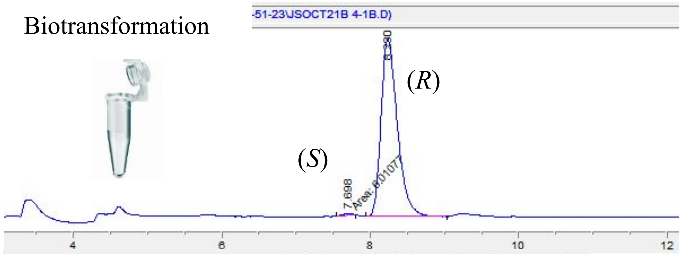

#	Time	Area	Height	Width	Area%	Symmetry
1	7.698	6	6.3E-1	0.1597	0.742	1.067
2	8.23	803.8	52.4	0.235	99.258	0.673

(*R*)- 1-(2-Phenylallyl)-1,2,3,4-tetrahydroisoquinoline, (*R*)-3n

The upper figures show the GC and corresponding MS trace for the imine intermediate **2a** and the racemic product standard (*rac*)-**3q**, along with the chiral HPLC chromatogram for (*rac*)-**3q.** Below is outlined the GC.MS and corresponding chiral HPLC chromatogram for the enantioselective chemoenzymatic allylation of **1a** with 4,4,5,5-Tetramethyl-2-(2-phenylallyl)-1,3,2-dioxaborolane.

GC-MS Data of imine intermediate and racemic product standard

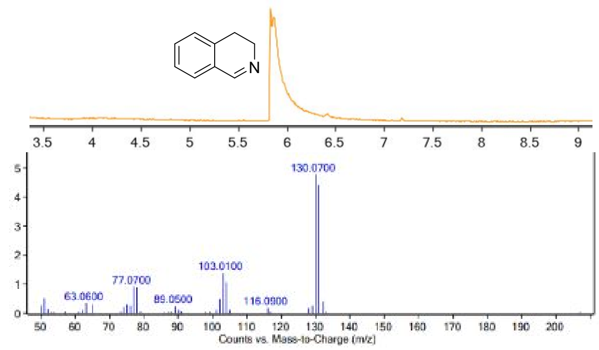

GC-MS (EI) m/z = 131

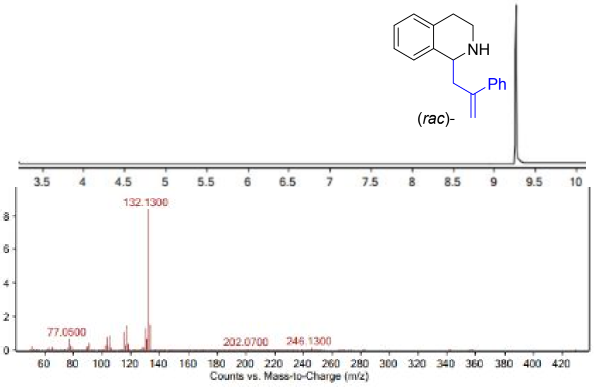

GC-MS (EI) m/z = 249

GC-MS Data from biotransformation

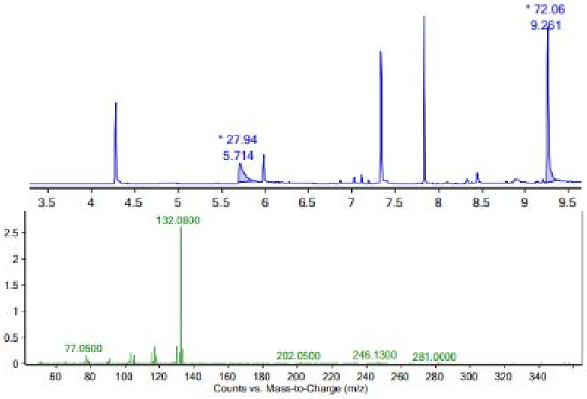

GC-MS (EI) m/z = 249

Chiral HPLC Data

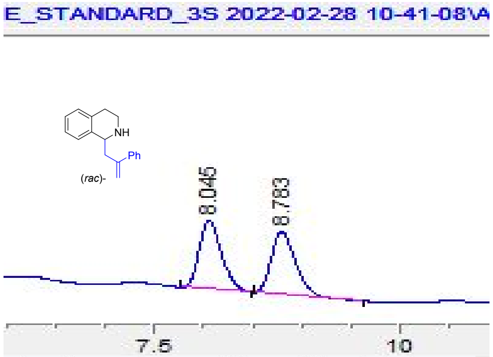

#	Time	Area	Height	Width	Area%	Symmetry
1	8.045	211.4	13.3	0.2452	49.768	0.741
2	8.783	213.3	12.3	0.2664	50.232	0.708

Biotransformation

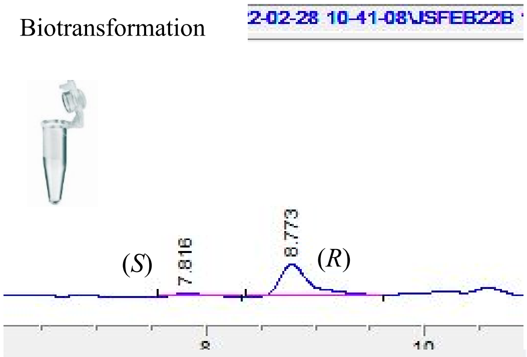

#	Time	Area	Height	Width	Area%	Symmetry
1	7.816	164.2	10.4	0.2358	8.892	0.622
2	8.773	1682.8	85.6	0.2893	91.108	0.535

(*R*)- 1-(But-2-en-1-yl)-1,2,3,4-tetrahydroisoquinoline, (*R*)-3o

The upper figures show the GC and corresponding MS trace for the imine intermediate **2a** and the racemic product standard (*rac*)-**3r**, along with the chiral HPLC chromatogram for (*rac*)-**3r.** Below is outlined the GC.MS and corresponding chiral HPLC chromatogram for the enantioselective chemoenzymatic allylation of **1a** with 2-(But-3-en-2-yl)-4,4,5,5-tetramethyl-1,3,2-dioxaborolane.

GC-MS Data of imine intermediate and racemic product standard

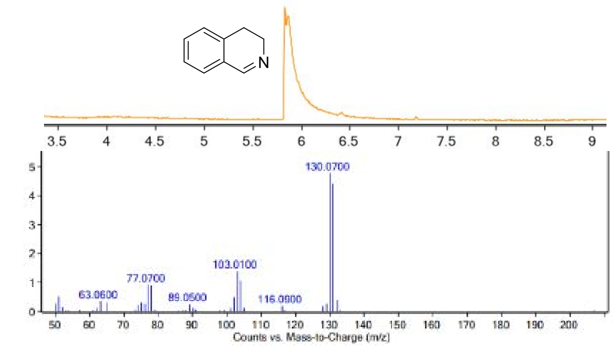

GC-MS (EI) m/z = 131

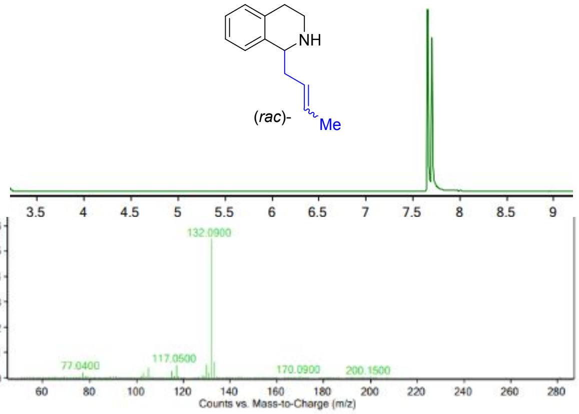

GC-MS (EI) m/z = 201

GC-MS Data from biotransformation

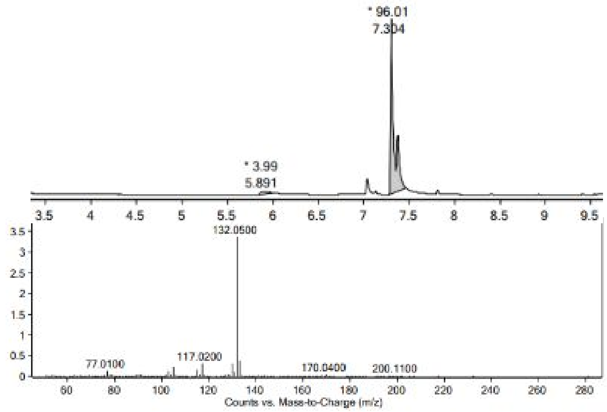

GC-MS (EI) m/z = 201

Chiral HPLC Data

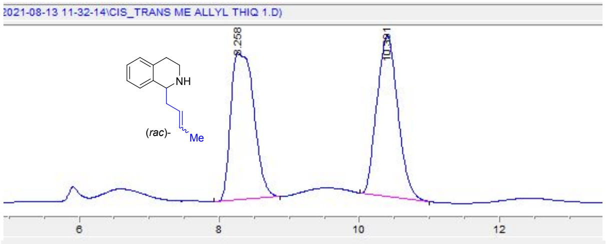

#	Time	Area	Height	Width	Area%	Symmetry
1	8.258	1013	44.2	0.3094	50.289	0.442
2	10.391	1001.3	48.2	0.3204	49.711	0.909

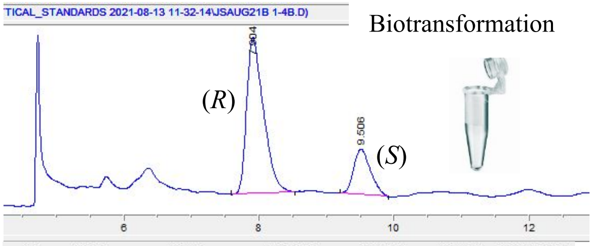

#	Time	Area	Height	Width	Area%	Symmetry
1	7.904	269	15.5	0.2524	77.347	0.555
2	9.506	78.8	4.5	0.2644	22.653	0.736

1-(But-3-en-2-yl)-1,2,3,4-tetrahydroisoquinoline, (*R*,*S*)-3p

The upper figures show the GC and corresponding MS trace for the imine intermediate **2a** and the racemic product standard (*rac*)-**3s**, along with the chiral HPLC chromatogram for (*rac*)-**3s.** Below is outlined the GC.MS and corresponding chiral HPLC chromatogram for the enantioselective chemoenzymatic allylation of **1a** with *cis*-crotyl boronic acid pinacol ester.

GC-MS Data of imine intermediate and racemic product standard

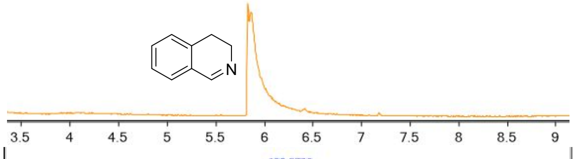

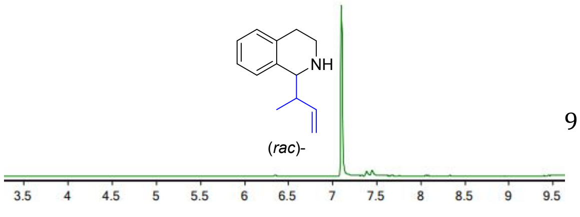

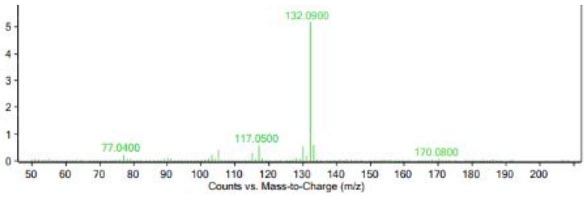

GC-MS (EI) m/z = 131

GC-MS (EI) m/z = 187

GC-MS Data from biotransformation

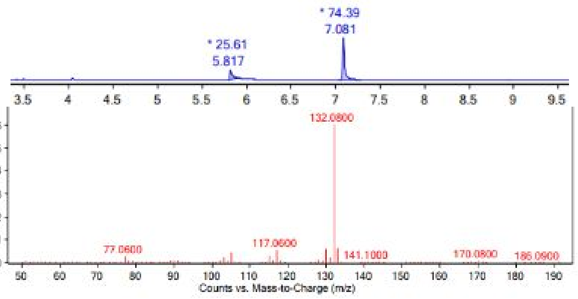

GC-MS (EI) m/z = 187

Chiral HPLC Data

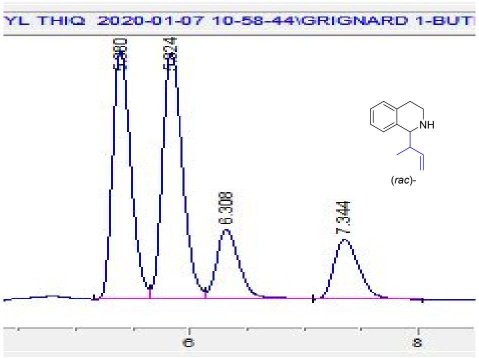

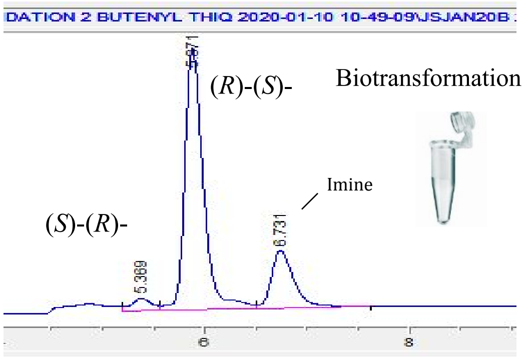

Mechanism outlining how the diastereoselectivity arises in the cascade for (*R*,*S*)-3p

Chemical addition to cyclic imine with *cis*-crotyl BPin

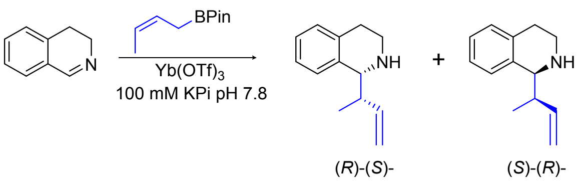

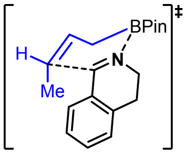

Chiral HPLC Data

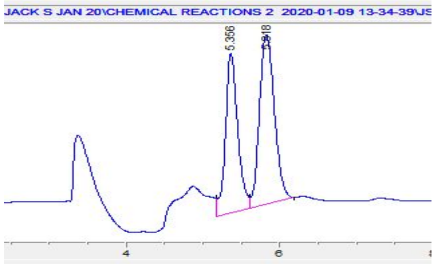

Favoured chair TS

The upper scheme outlines the diastereoselectivity which is observed during the chemical addition of *cis*-crotyl BPin to 3,4-dihydroisoquinoline. This diastereoselectivity has been previously outlined in literature, and results from the orientation of the methyl group of the BPin in the favoured chair transition state during the reaction.[18] The chiral HPLC traces clearly shows the formation of only two diastereomers, which can be compared to the HPLC trace of (*rac*)-**3s** showing all four diastereomers. The scheme below shows the chemoenzymatic cascade for 1,2,3,4-tetrahydroisoquinoline and *cis*-crotyl BPin. As described above, the cascade initially generates the (*R,S*)- and (*S,R*)-diastereomers. The next step involves the selective oxidation of the (*S,R*)-diastereomer to the corresponding imine, catalysed by MAO-N D11. The chiral imine which is generated then undergoes *in situ* epimerization, a process which has been observed previously in our group.[19] The IRED is then able to preferentially reduce a single enantiomer of the chiral imine, leading to the accumulation of the (*R,S*)-product.

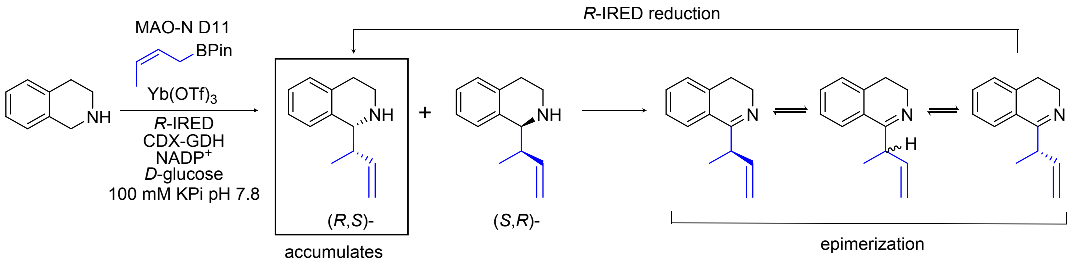

(*R*)-1-(2-Methylbut-3-en-2-yl)-1,2,3,4-tetrahydroisoquinoline, 3q

The upper figures show the GC and corresponding MS trace for the imine intermediate **2a**. Below is outlined the GC.MS for the enantioselective chemoenzymatic allylation of **1a** with gem-dimethyl allyl boronic ester.

GC-MS Data of imine intermediate

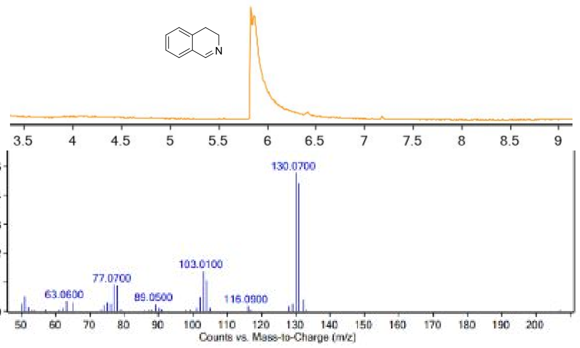

GC-MS (EI) m/z = 131

GC-MS Data from biotransformation

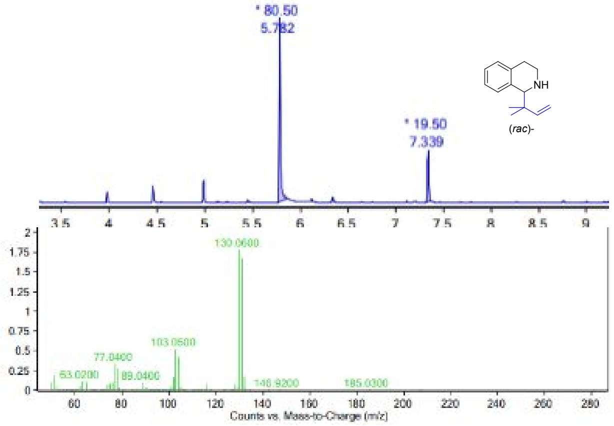

GC-MS (EI) m/z = 201

(*R*)-1-Prop-2-yn-1-yl-1,2,3,4-tetrahydroisoquinoline, (*R*,*S*)-3r

The upper figures show the GC and corresponding MS trace for the imine intermediate **2a** and the racemic product standard (*rac*)-**3u**, along with the chiral HPLC chromatogram for (*rac*)-**3u.** Below is outlined the GC.MS and corresponding chiral HPLC chromatogram for the enantioselective chemoenzymatic allylation of **1a** with 2-(buta-2,3-dien-1-yl)-4,4,5,5-tetramethyl-1,3,2-dioxaborolane.

GC-MS Data

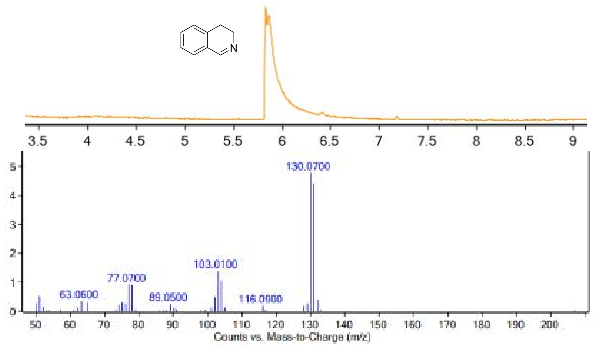

GC-MS (EI) m/z = 131

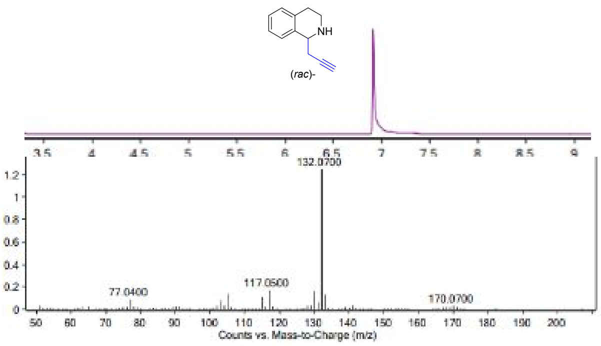

GC-MS (EI) m/z = 171

GC-MS Data from biotransformation

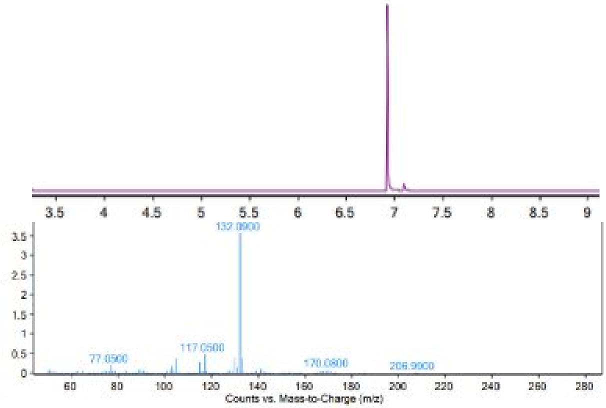

GC-MS (EI) m/z = 171

Chiral HPLC Data

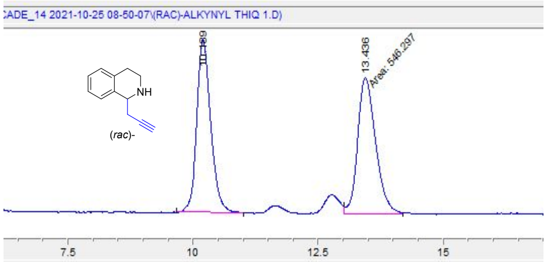

#	Time	Area	Height	Width	Area%	Symmetry
1	10.189	527.6	27.2	0.2925	49.129	0.762
2	13.436	546.3	21.6	0.4219	50.871	0.728

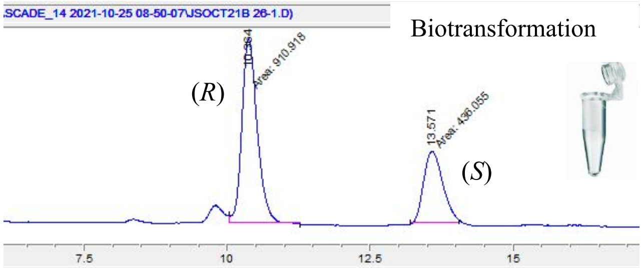

#	Time	Area	Height	Width	Area%	Symmetry
1	10.364	910.9	48.3	0.3144	67.627	0.739
2	13.571	436.1	18.8	0.3866	32.373	0.739

7. Preparative scale biotransformations

7.1. Chemoenzymatic synthesis of (*R*)-1-allyl-1,2,3,4-tetrahydroisoquinoline, (*R*)-3a

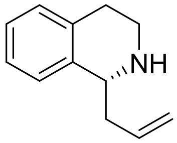

To a 50 mL falcon tube was added 15 mM of 1,2,3,4-tetrahhydroisoquinoline (1 M in DMSO) along with 120 mM allyl BPin (1 M in DMSO) and 10 mol% $Yb(OTf)_3$ (100 mM in dH_2O). 40 mM *D*-glucose (200 mM stock in 100 mM KPi pH 7.8) and 0.4 mM $NADP^+$ (100 mM in 100 mM KPi pH 7.8) were added along with 1 mg mL^{-1} of CDX-GDH and 2 mg mL^{-1} of purified MAO-N D11. Finally, 10 mg mL^{-1} of R-IRED cfe was added and the volume made up to 40 mL with 100 mM KPi pH 7.8. Biotransformations were incubated at 30 °C for 48 h with 200 rpm shaking. The reaction was quenched by the addition of 0.5 mL 10M NaOH followed by centrifugation at 4000 rpm for 5 minutes, this was repeated a further two times. The aqueous components were collected and extracted with MTBE (3 x 40 mL). The combined organic layers were dried over anhydrous magnesium sulphate, filtered, and concentrated *in vacuo* to furnish the desired product (61 mg, 0.60 mmol, 59%) as a dark orange oil. **^{1}H NMR** (400 MHz, $CDCl_3$) δ 7.19-7.07 (m, 4H, ArCH), 5.86 (dddd, *J*=16.9, 10.2, 7.8, 6.5, 1H, CH=), 5.12 (m, 2H, $=CH_2$), 3.98 (m, 1H, CH), 3.50 (m, 1H, CH_2), 2.92 (m, 1H, CH_2), 2.74 (m, 2H, CH_2), 2.60 (m, 1H, CH_2), 2.45 (m, 1H, CH_2). Data consistent with the literature.[9] Enantiomeric excess was determined by chiral HPLC and compared to a genuine racemic chemical standard. $[\alpha]^{25}_{589}$ = -106.61 (98% *ee*, c = 0.045, $CHCl_3$) [lit.[9] (*S* enantiomer): $[\alpha]^{27}_{589}$ = +121.24 (94.8% *ee*, c 1.43, $CHCl_3$).

7.2. Chemoenzymatic synthesis of 1-(2-methylenebutyl)-1,2,3,4-tetrahydroisoquinoline, (*R*)-3l

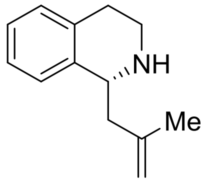

To a 50 mL falcon tube was added 15 mM of 1,2,3,4-tetrahhydroisoquinoline (1 M in DMSO) along with 120 mM 4,4,5,5-tetramethyl-2-(2-methylenebutyl)-1,3,2-dioxaborolane. (1 M in DMSO) and 10 mol% $Yb(OTf)_3$ (100 mM in dH_2O). 40 mM *D*-glucose (200 mM stock in 100 mM KPi pH 7.8) and 0.4 mM $NADP^+$ (100 mM in 100 mM KPi pH 7.8) were added along with 1 mg mL^{-1} of CDX-GDH and 2 mg mL^{-1} of purified MAO-N D11. Finally, 10 mg mL^{-1} of R-IRED cfe was added and the volume made up to 40 mL with 100 mM KPi pH 7.8. Biotransformations were incubated at 30 °C for 48 h with 200 rpm shaking. The reaction was quenched by the addition of 0.5 mL 10M NaOH followed by centrifugation at 4000 rpm for 5 minutes, this was repeated a further two times. The aqueous components were collected and extracted with MTBE (3 x 40 mL). The combined organic layers were dried over anhydrous magnesium sulphate, filtered, and concentrated *in vacuo* to furnish the desired product (71 mg, 0.38 mmol, 64%) as a yellow oil. **^{1}H NMR** (400 MHz, $CDCl_3$) δ 7.21 (m, 4H, ArCH), 5.04 (s, 1H, H_2C=), 4.98 (s, 1H, H_2C=), 4.61 (t, *J*= 7.2 Hz, 1H, CH), 3.56 (m, 1H, CH_2), 3.42 (m, 1H, CH_2), 3.12 (m, 2H, CH_2), 2.69 (m, 2H, CH_2), 1.82 (s, 3H, CH_3). Enantiomeric excess was determined by chiral HPLC and compared to a genuine racemic chemical standard. $[\alpha]^{25}_{589}$ = -85.71 (98% *ee*, c = 0.045, $CHCl_3$).

8. Time course Experiments

To improve our understanding of the reaction, we carried out a series of time course experiments, where we monitored the conversion and enantioselectivity of the formation of 1-allyl-1,2,3,4-tetrahydroisoquinoline **3a** from 1,2,3,4-tetrahydroisoquinoline **1a** over time (Figure 2a).

After only 6h the conversion reached 90% and 90% *ee*, and after 12 hours only the desired product and enantiomer could be observed (>99% *ee*). Interestingly, during these reactions no imine **2a** was observed, therefore we ran our time course monitoring the formation of the imine intermediate 3,4-dihydroisoquinoline **2a** (Figure 2b) for comparison. This suggests that during our MAO-N/IRED cascade conditions, as soon as intermediate **2a** is formed it reacts with the boryl reagent to form the racemic product, alternatively it is reduced back to the starting material by R-IRED to begin the cycle again.

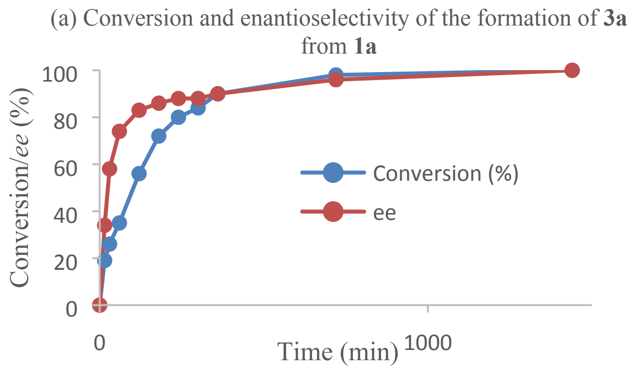

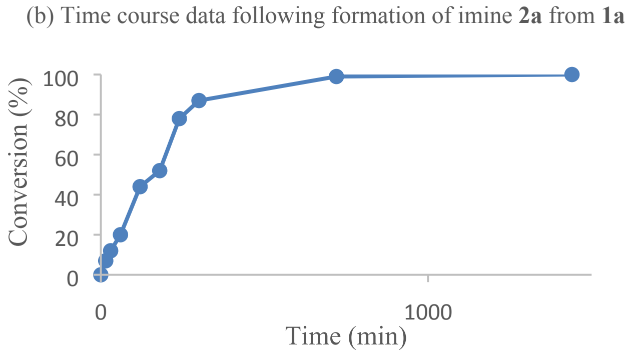

Figure 2 Time course experiments following (a) the formation of the desired product 3a from 1a, and (b) the formation of imine 2a from 1a.

9. NMR analysis

9.1. NMR spectra of novel chemical standards

1-Allyl-6-bromo-1,2,3,4-tetrahydroisoquinoline, (*rac*)-3b

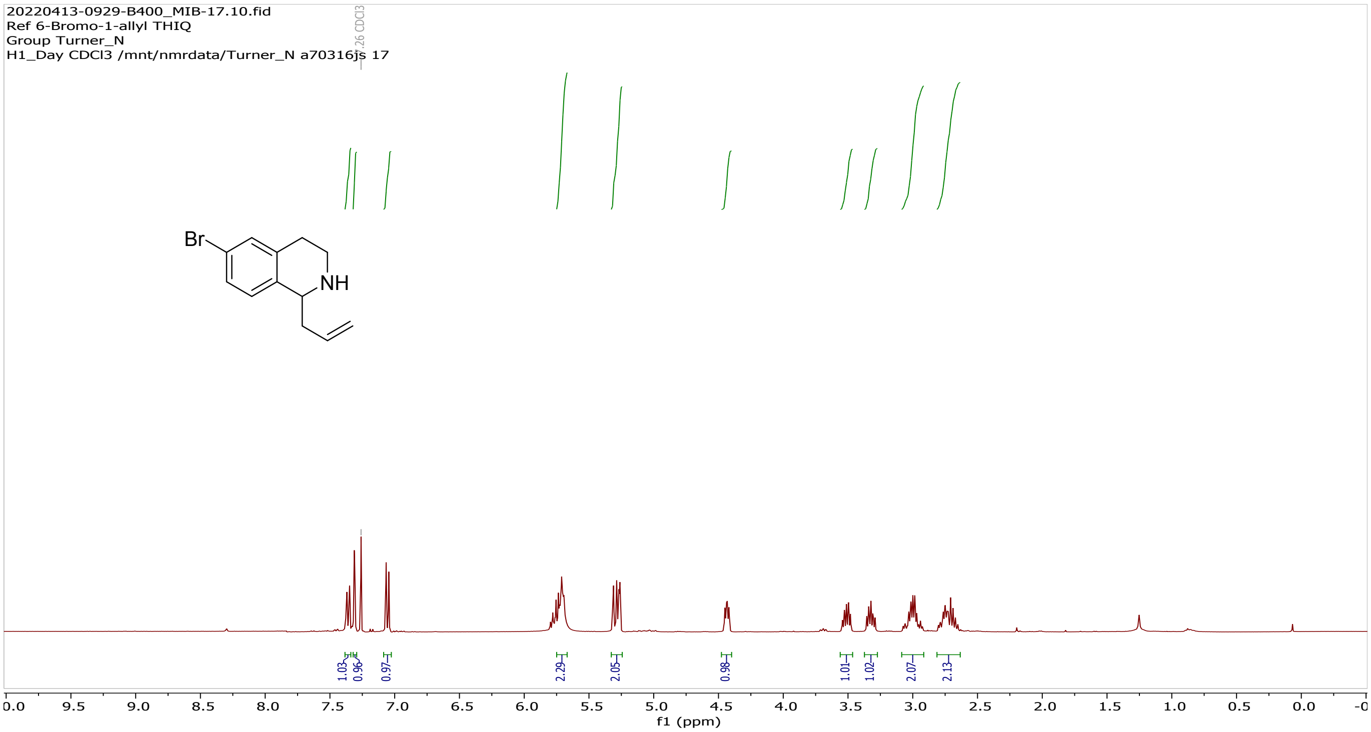

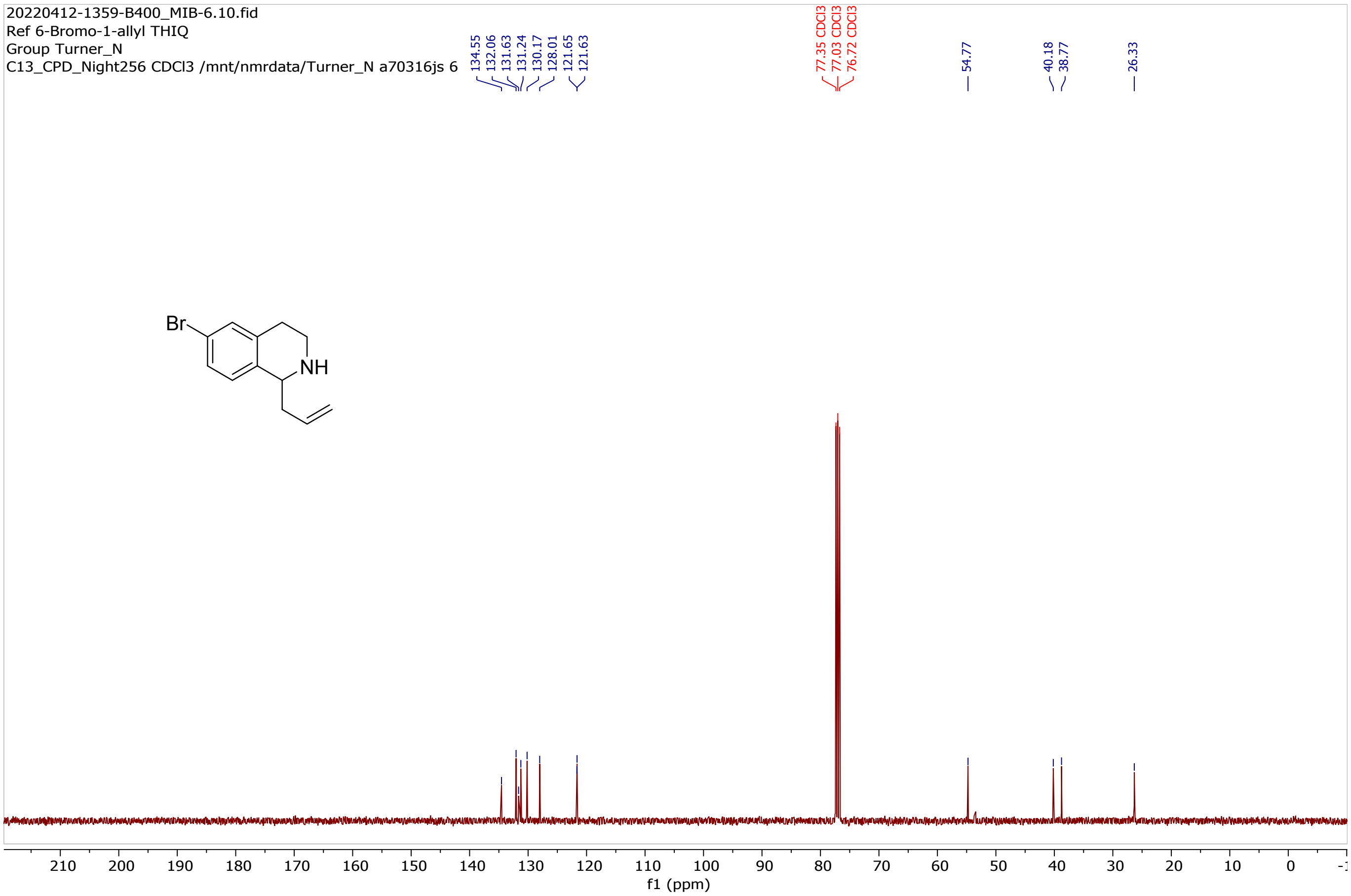

1-Allyl-6-chloro-1,2,3,4-tetrahydroisoquinoline, (*rac*)-3c

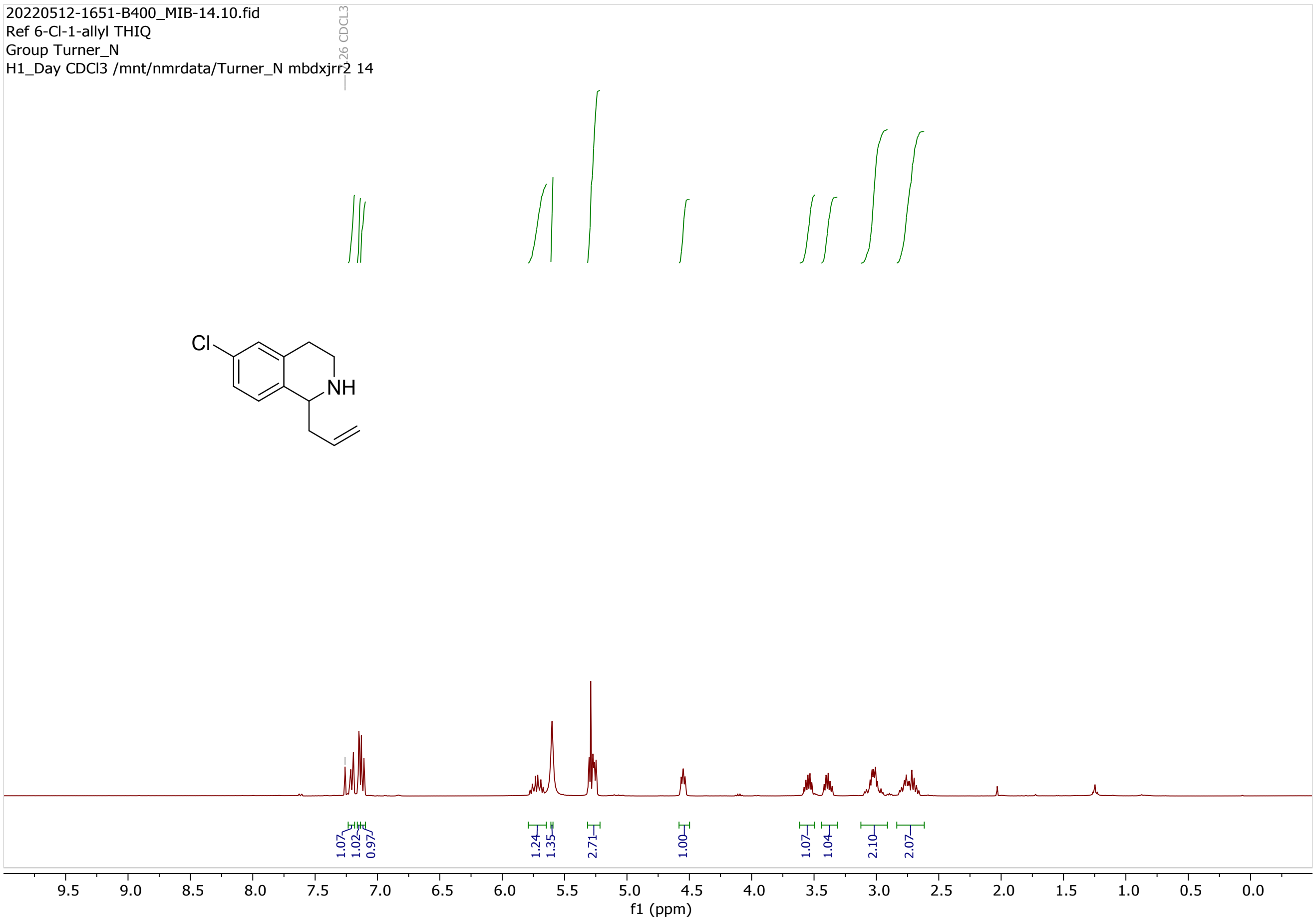

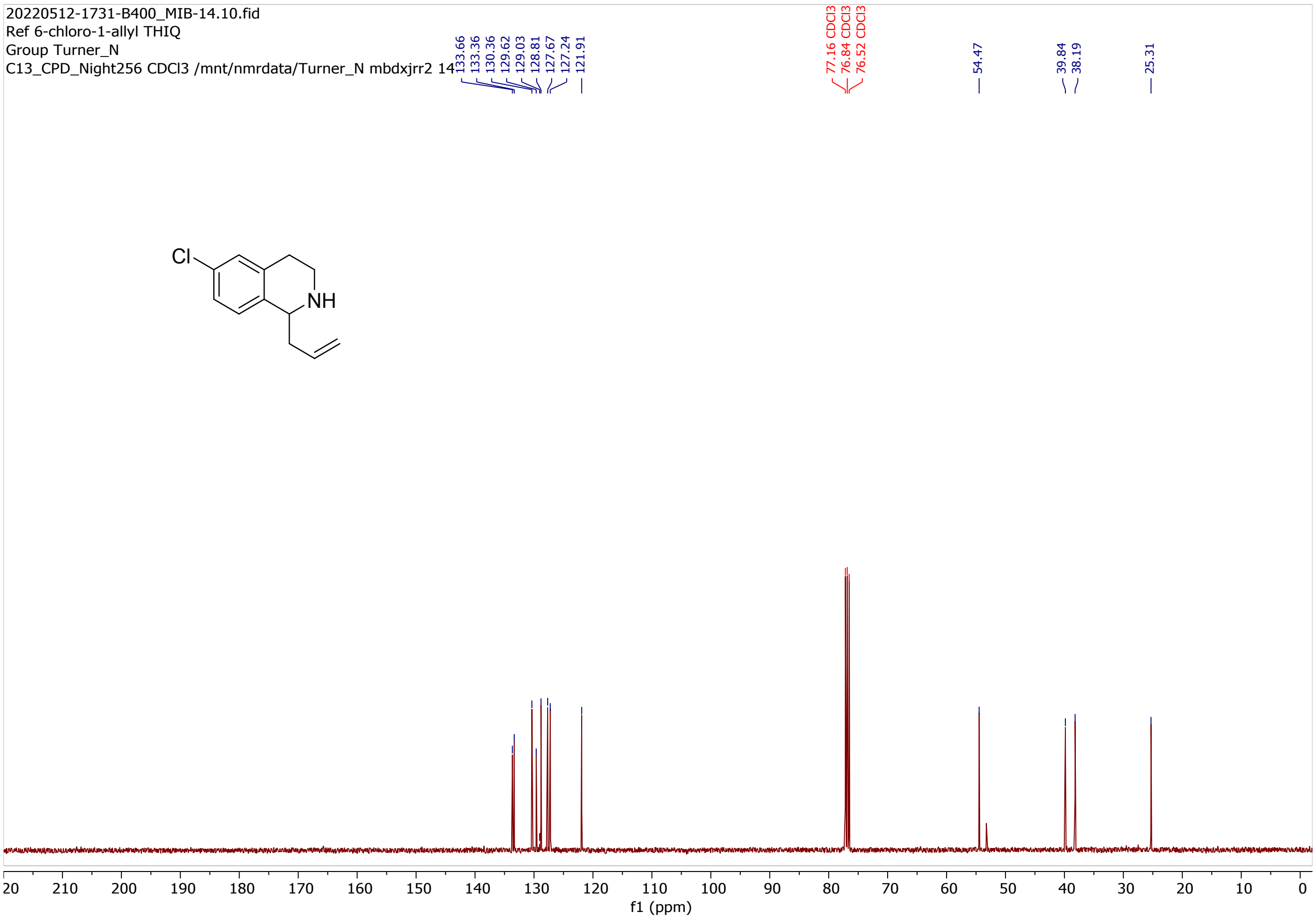

1-Allyl-6-methoxy 1,2,3,4-tetrahydroisoquinoline, (*rac*)-3e

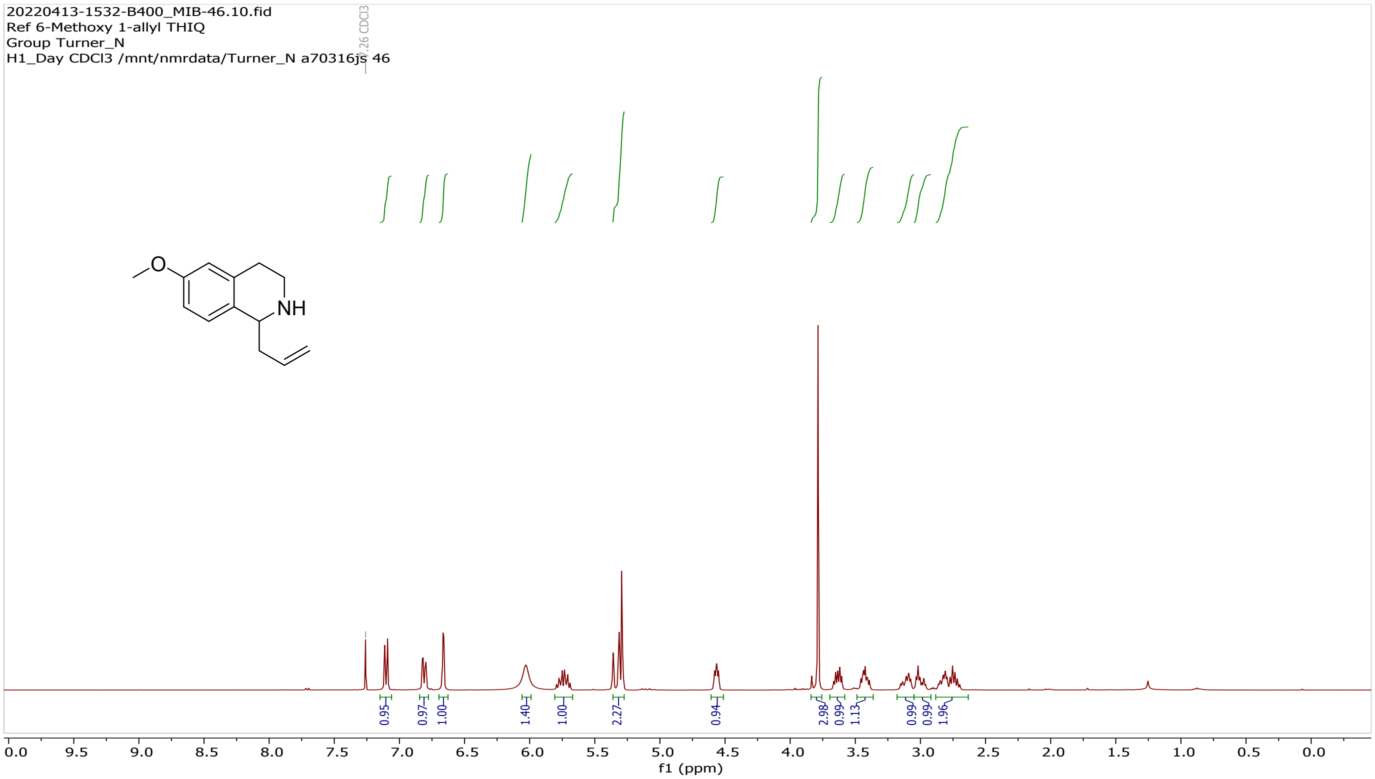

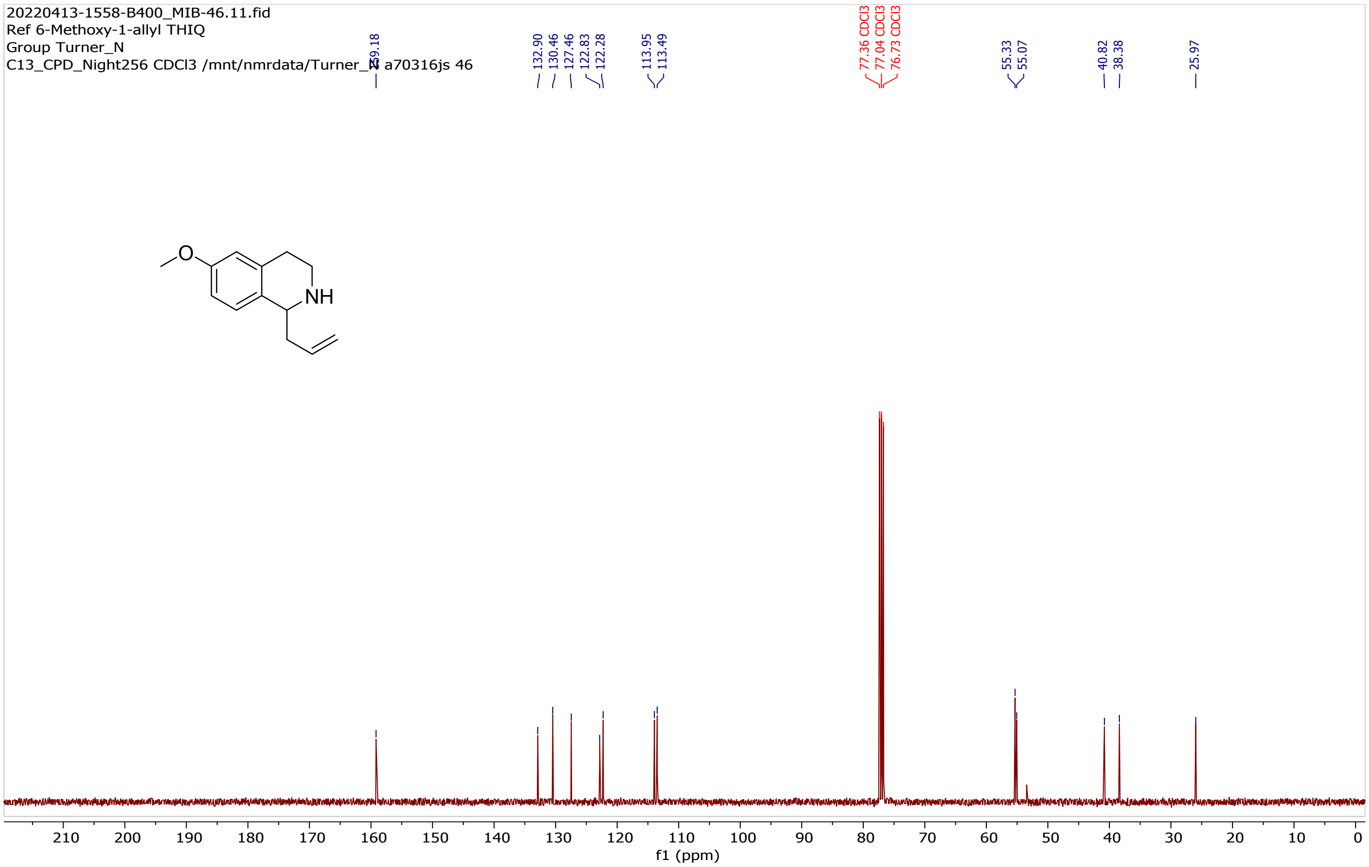

1-Allyl-8-methyl-1,2,3,4-tetrahydroisoquinoline, (*rac*)-3i

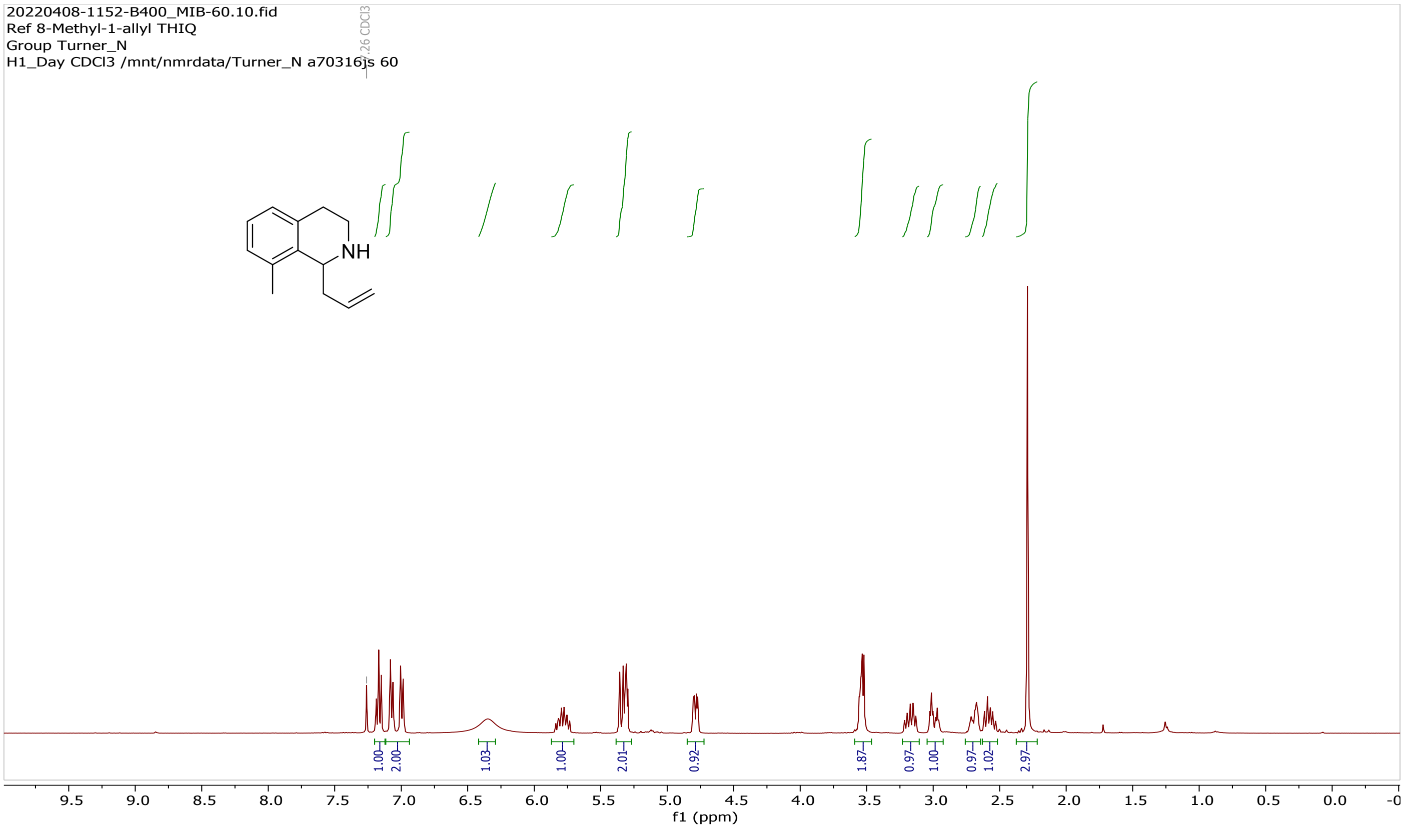

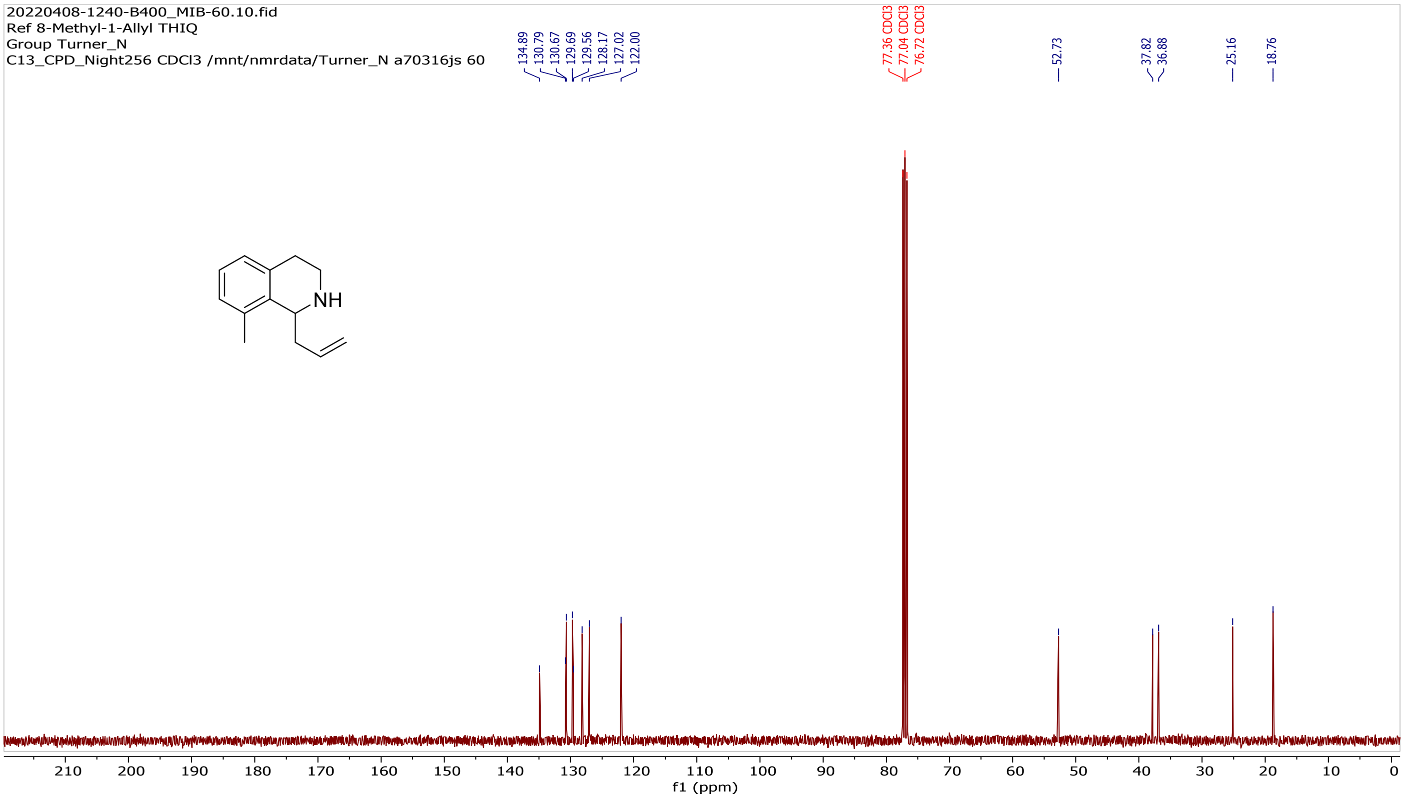

1-Allyl-8-bromo-1,2,3,4-tetrahydroisoquinoline, (*rac*)-3j

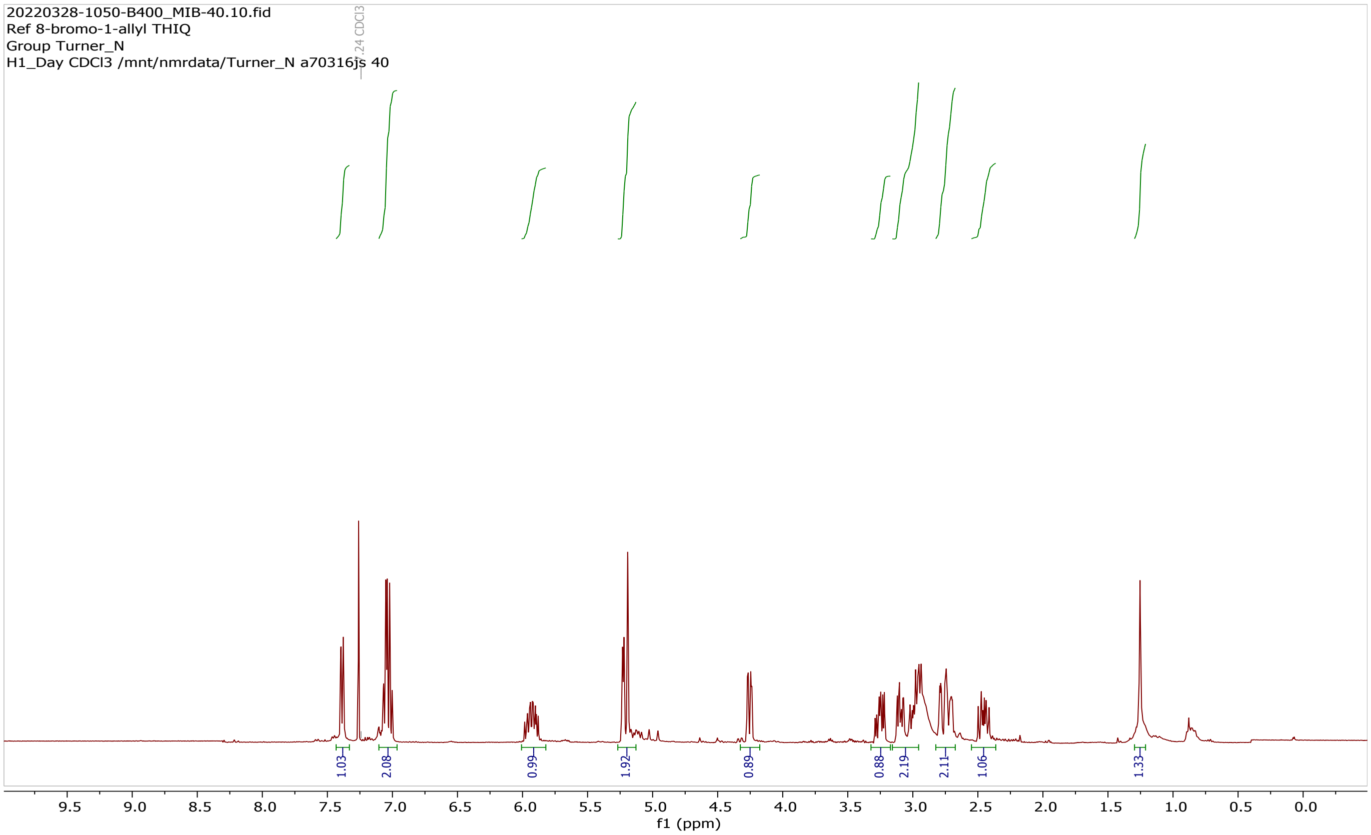

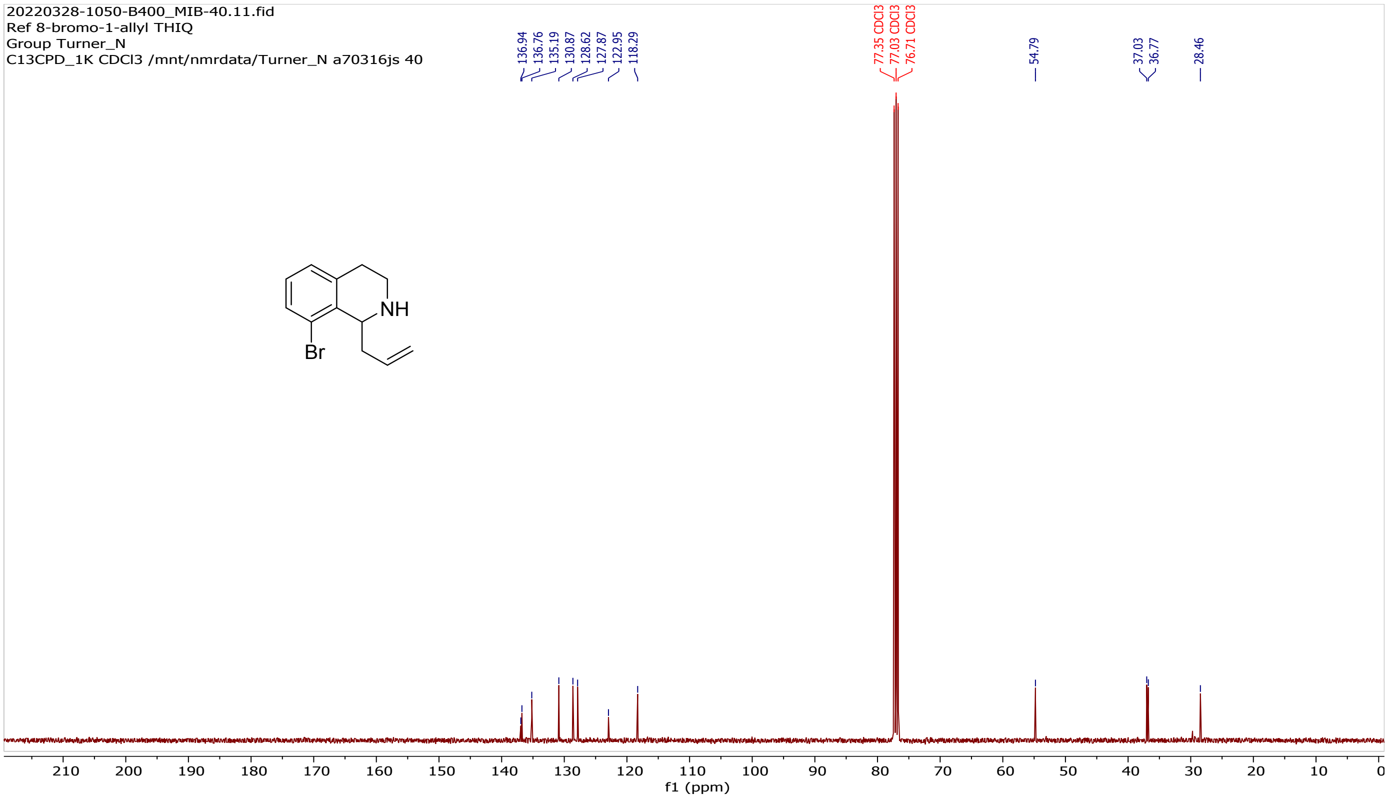

1-Allyl-8-bromo-1,2,3,4-tetrahydroisoquinoline, (*rac*)-3j

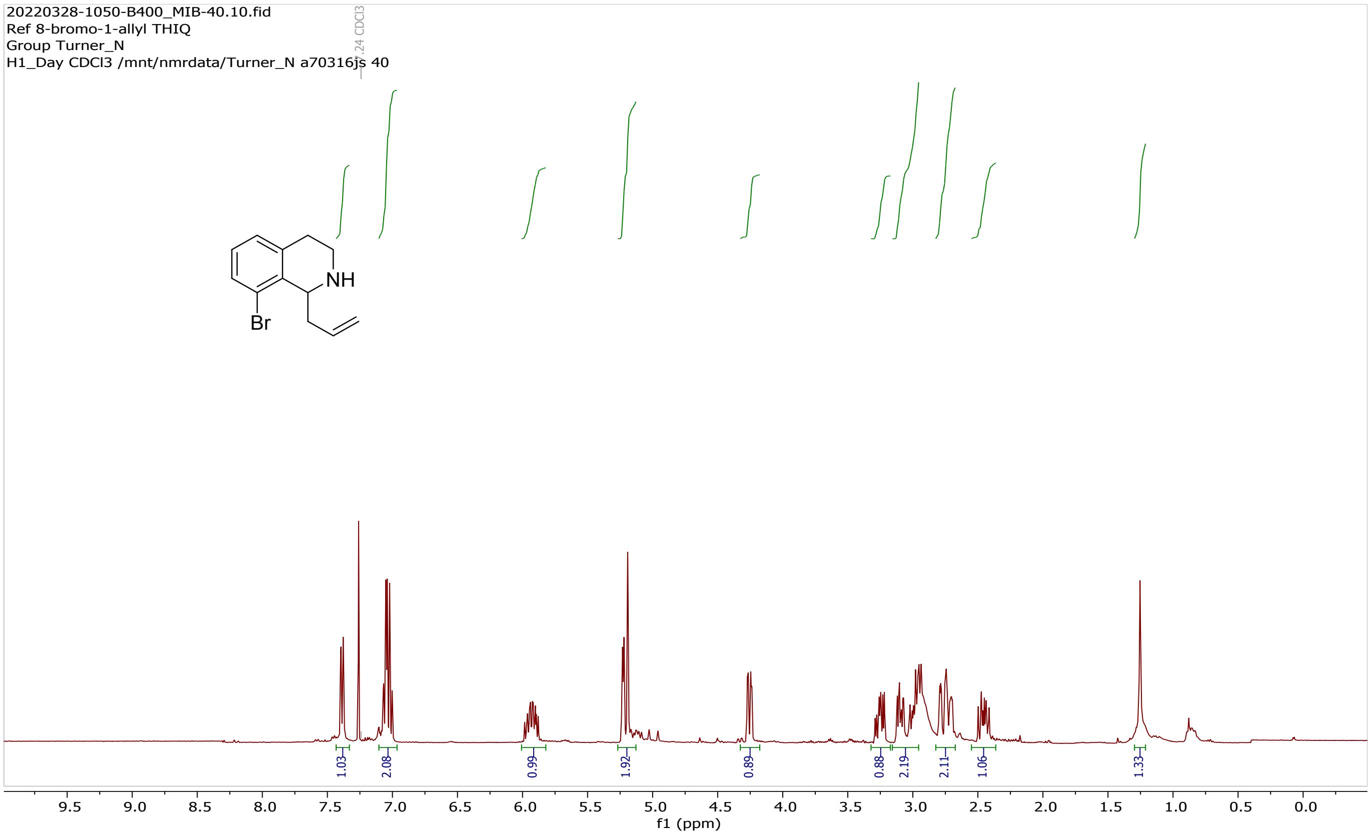

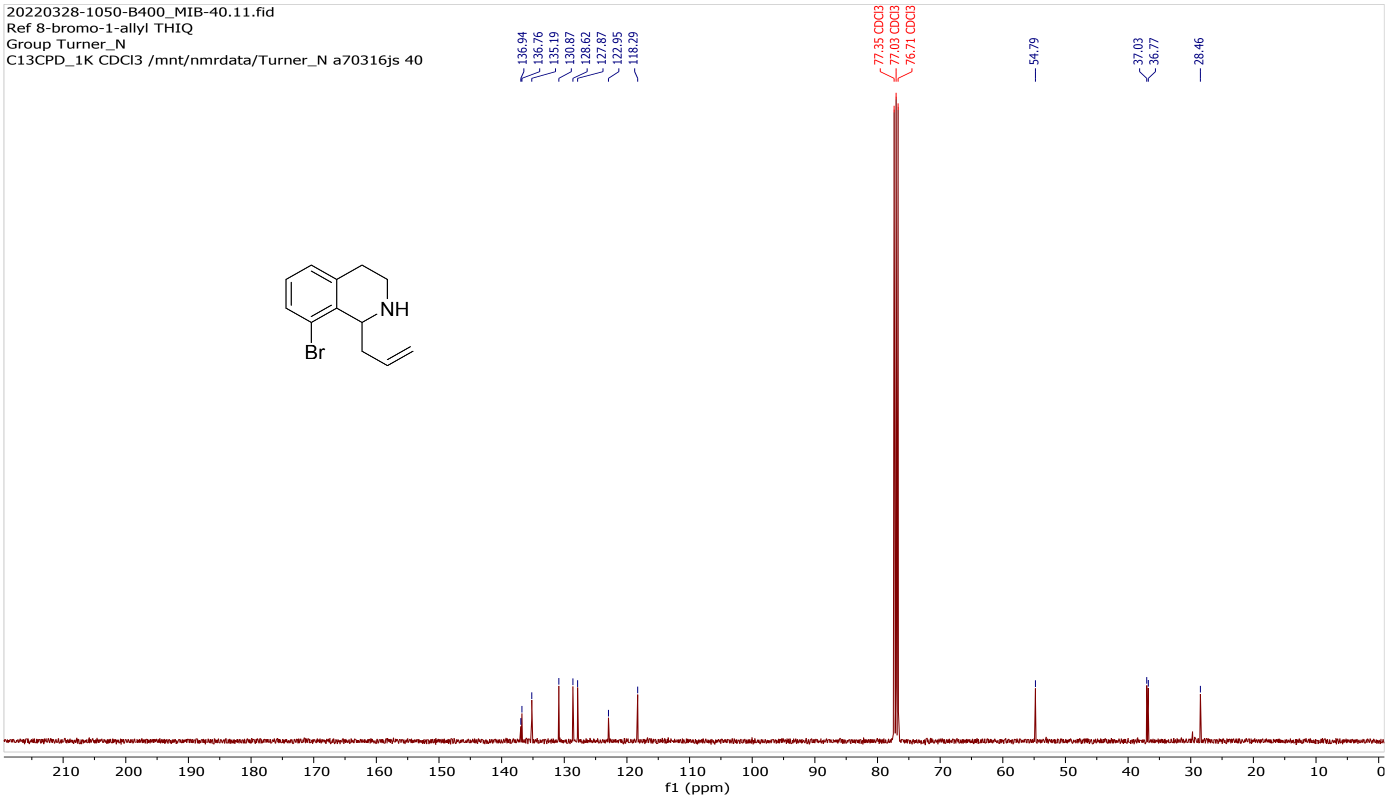

1-(2-Methylallyl)-1,2,3,4-tetrahydroisoquinoline, (*rac*)-3l

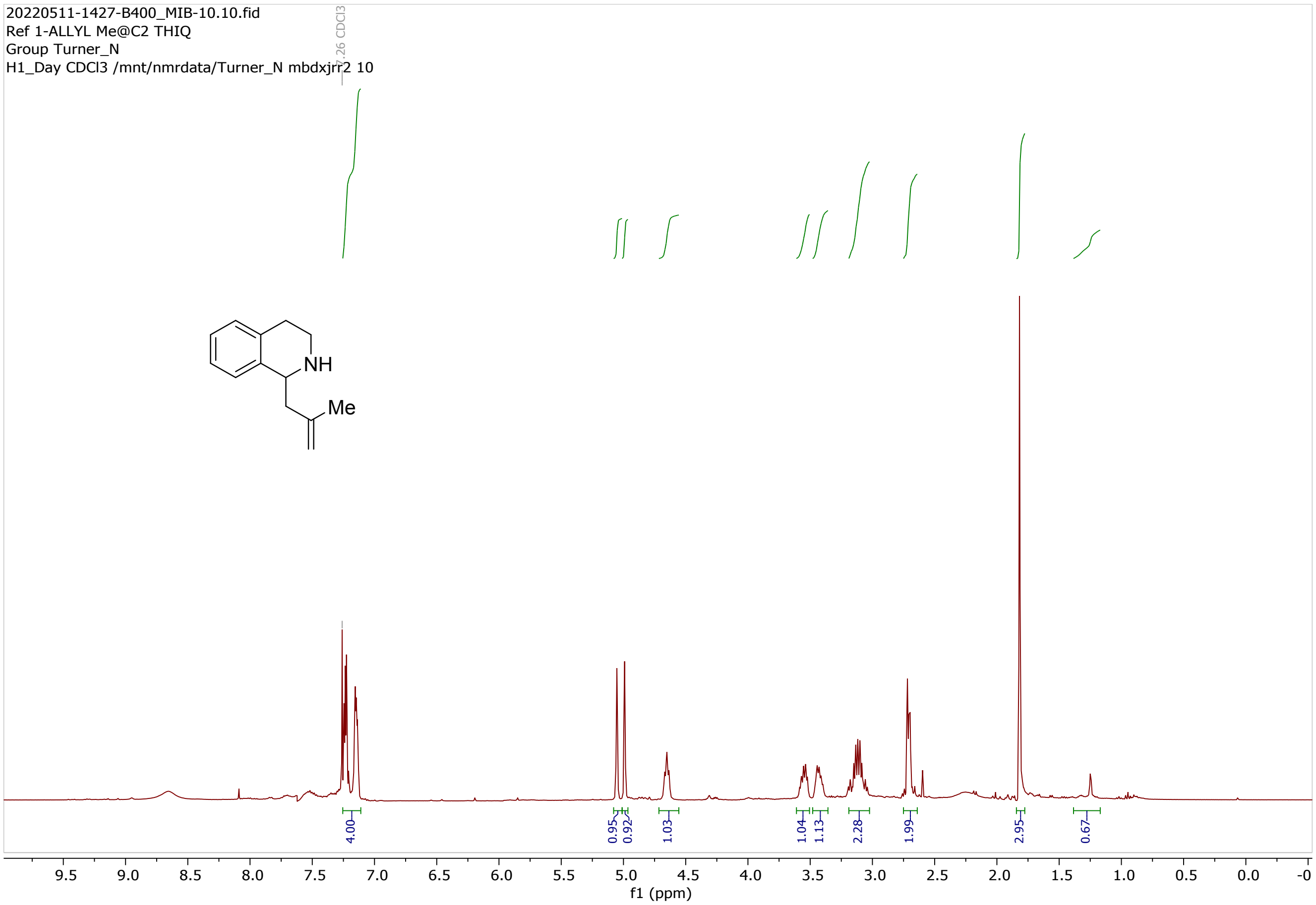

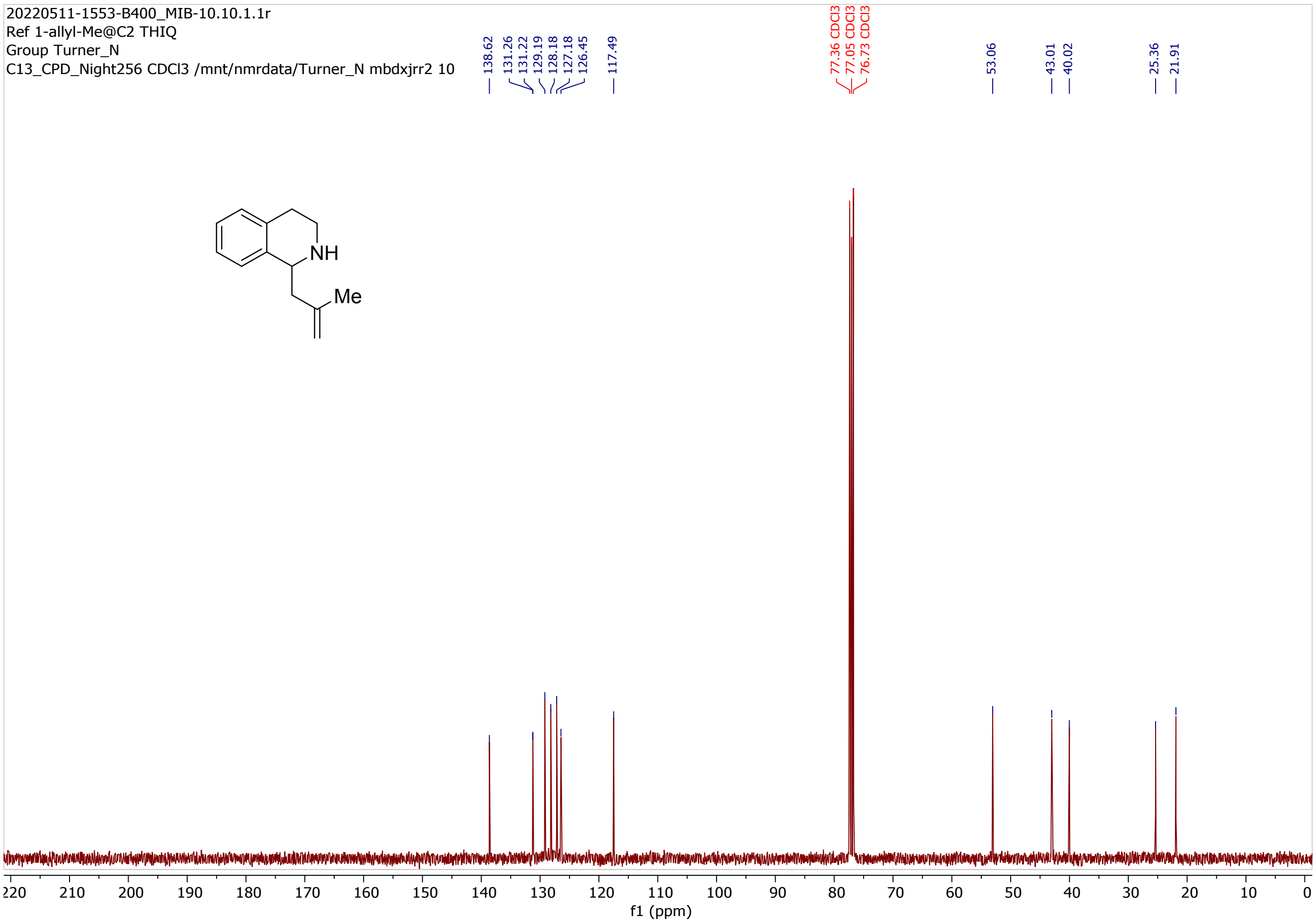

1-(2-Methylenebutyl)-1,2,3,4-tetrahydroisoquinoline, (*rac*)-3m

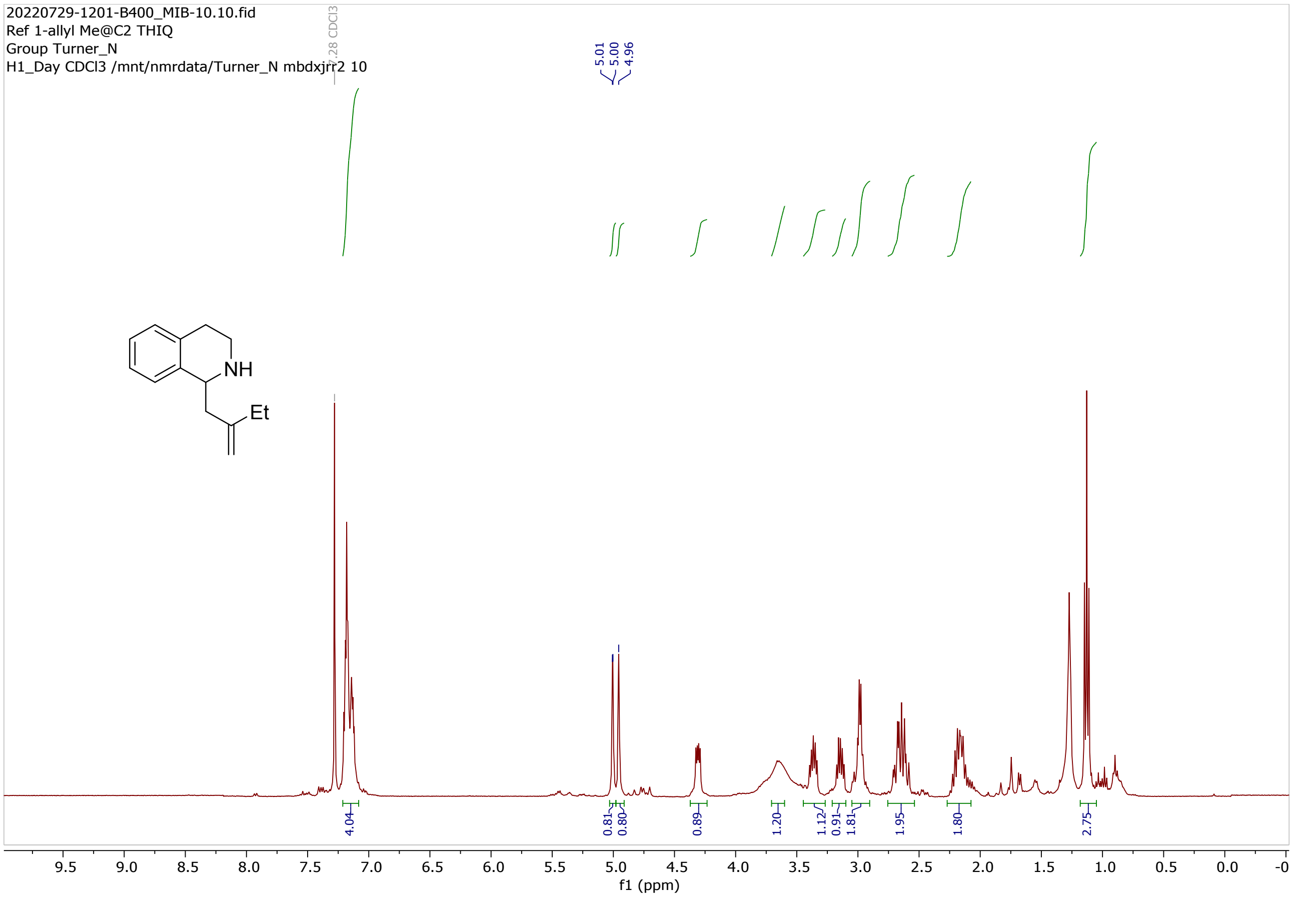

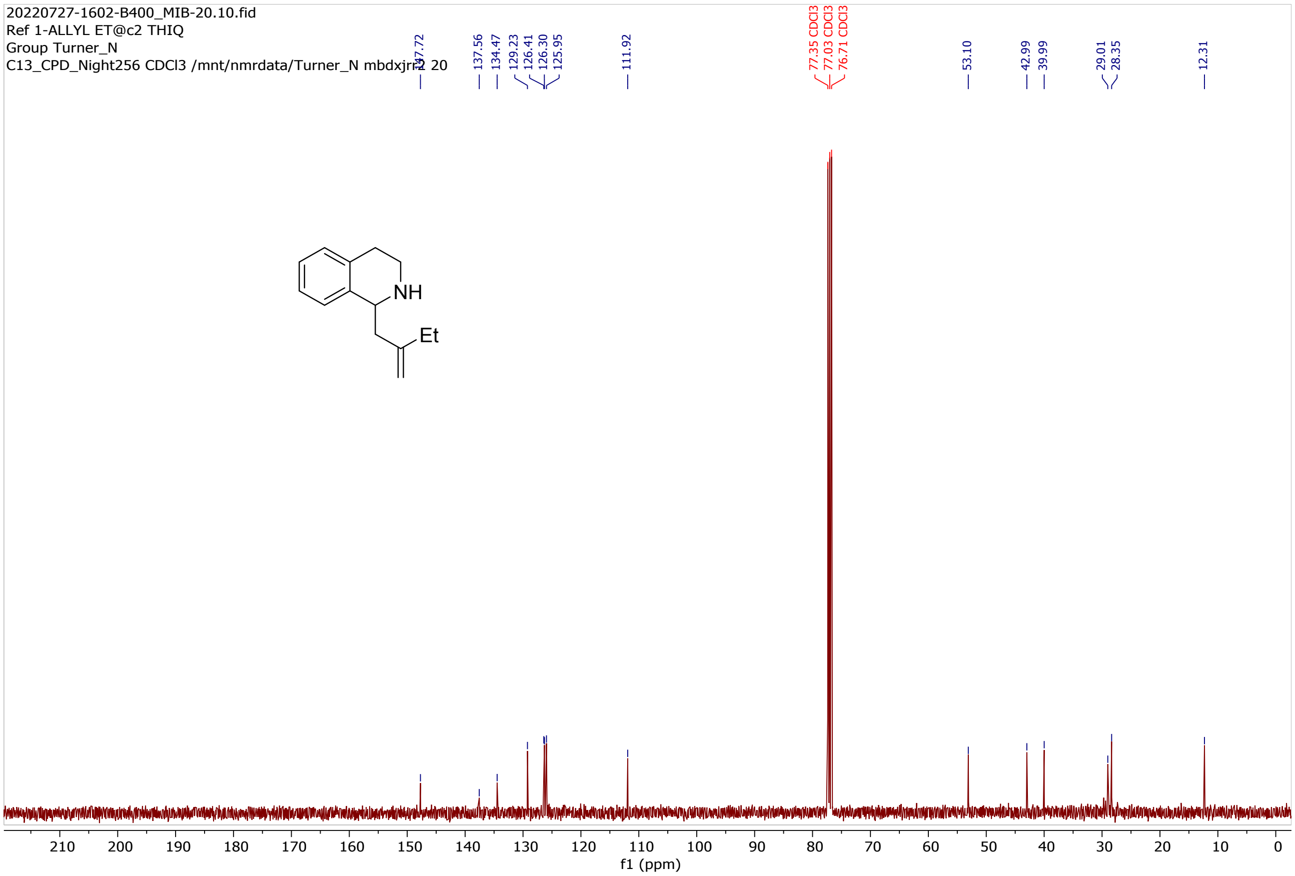

1-(2-Phenylallyl)-1,2,3,4-tetrahydroisoquinoline, (*rac*)-3n

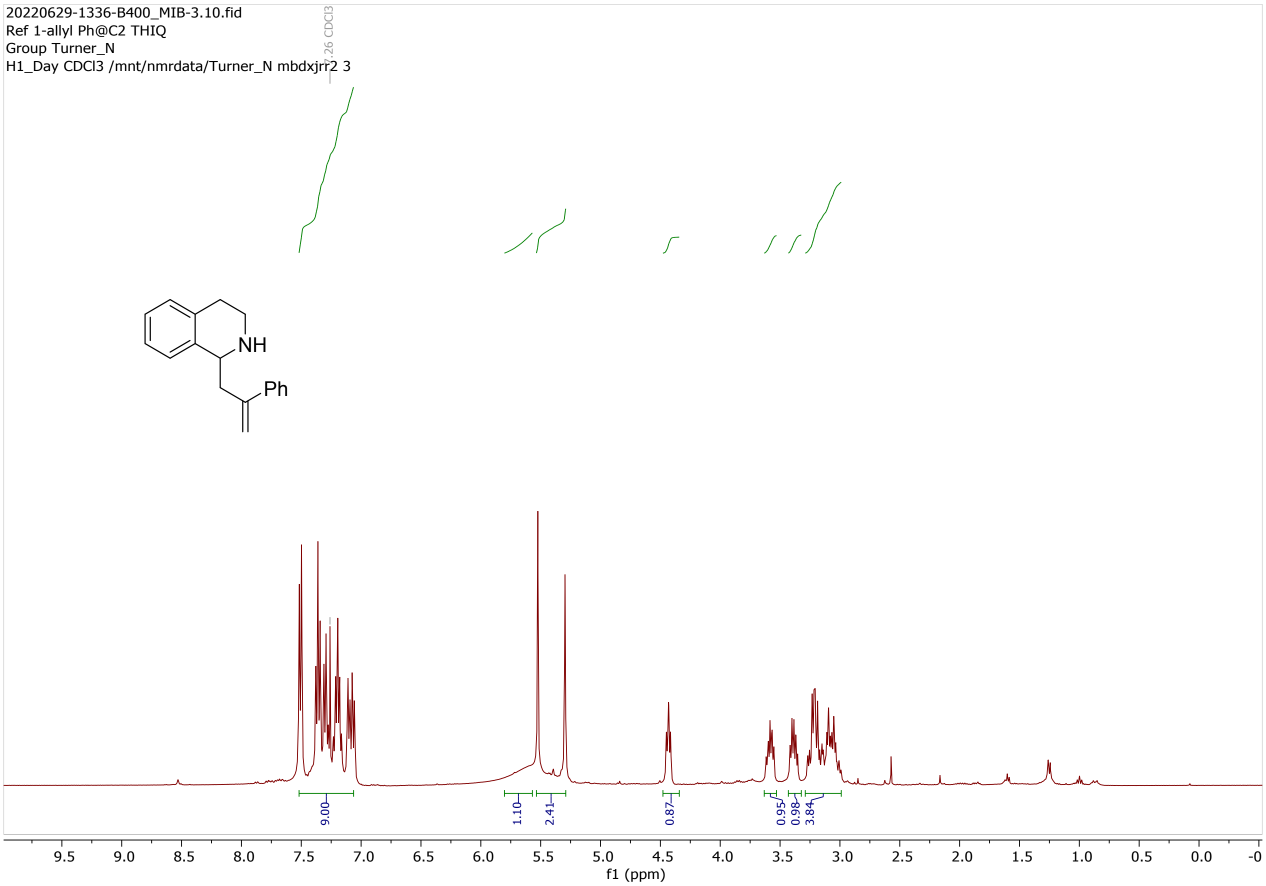

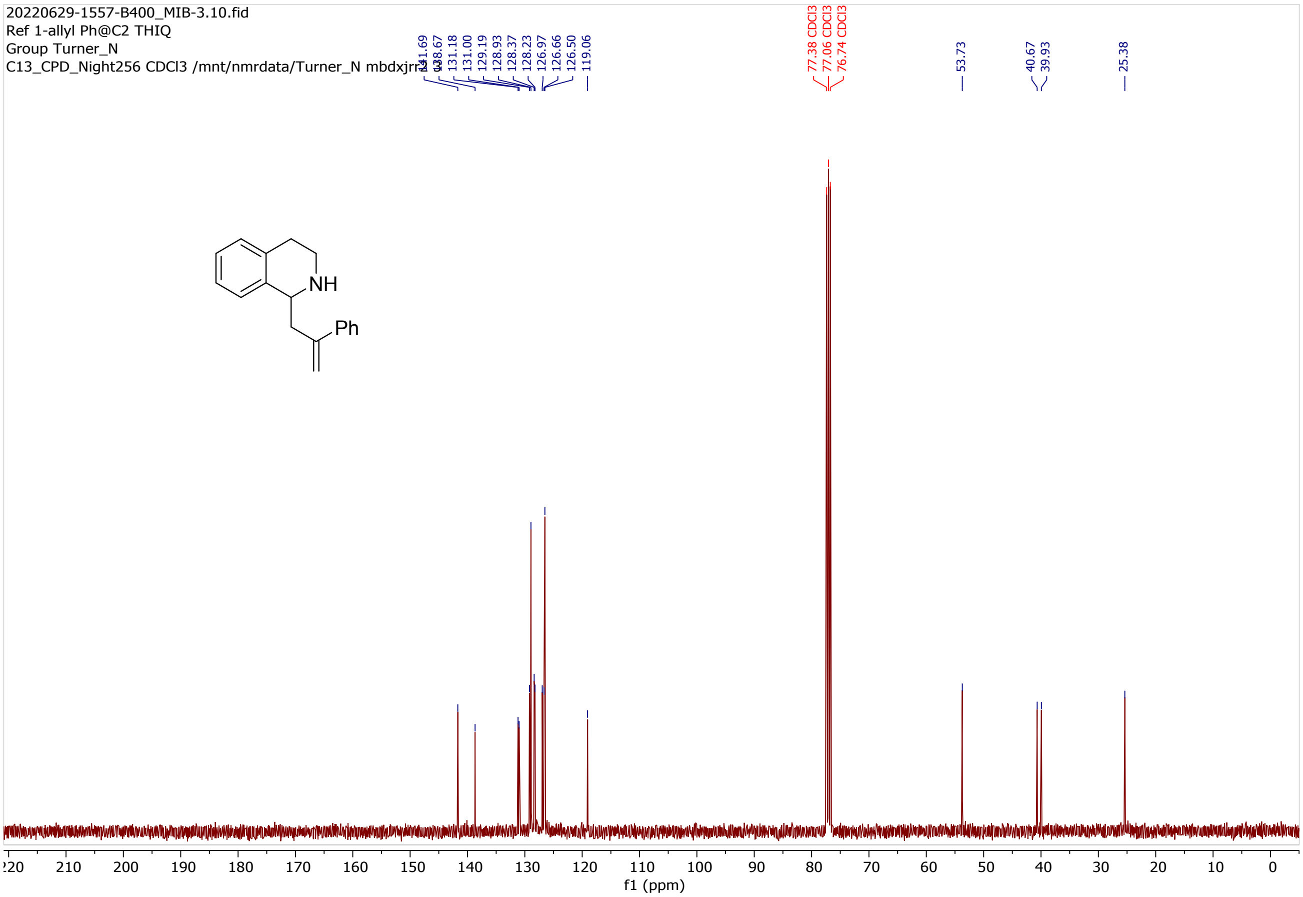

1-(2-Methylenebutyl)-1,2,3,4-tetrahydroisoquinoline, (*rac*)-30

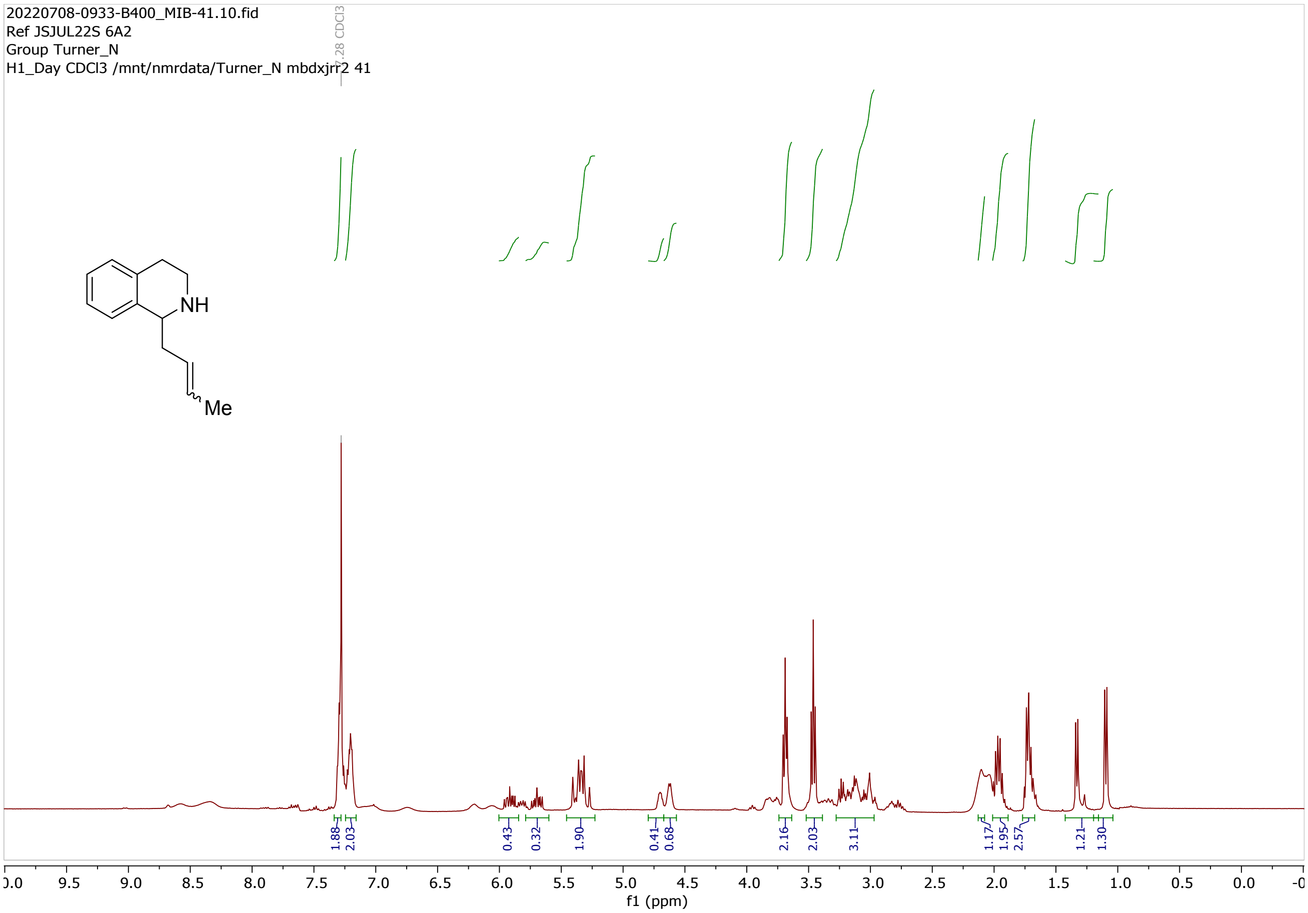

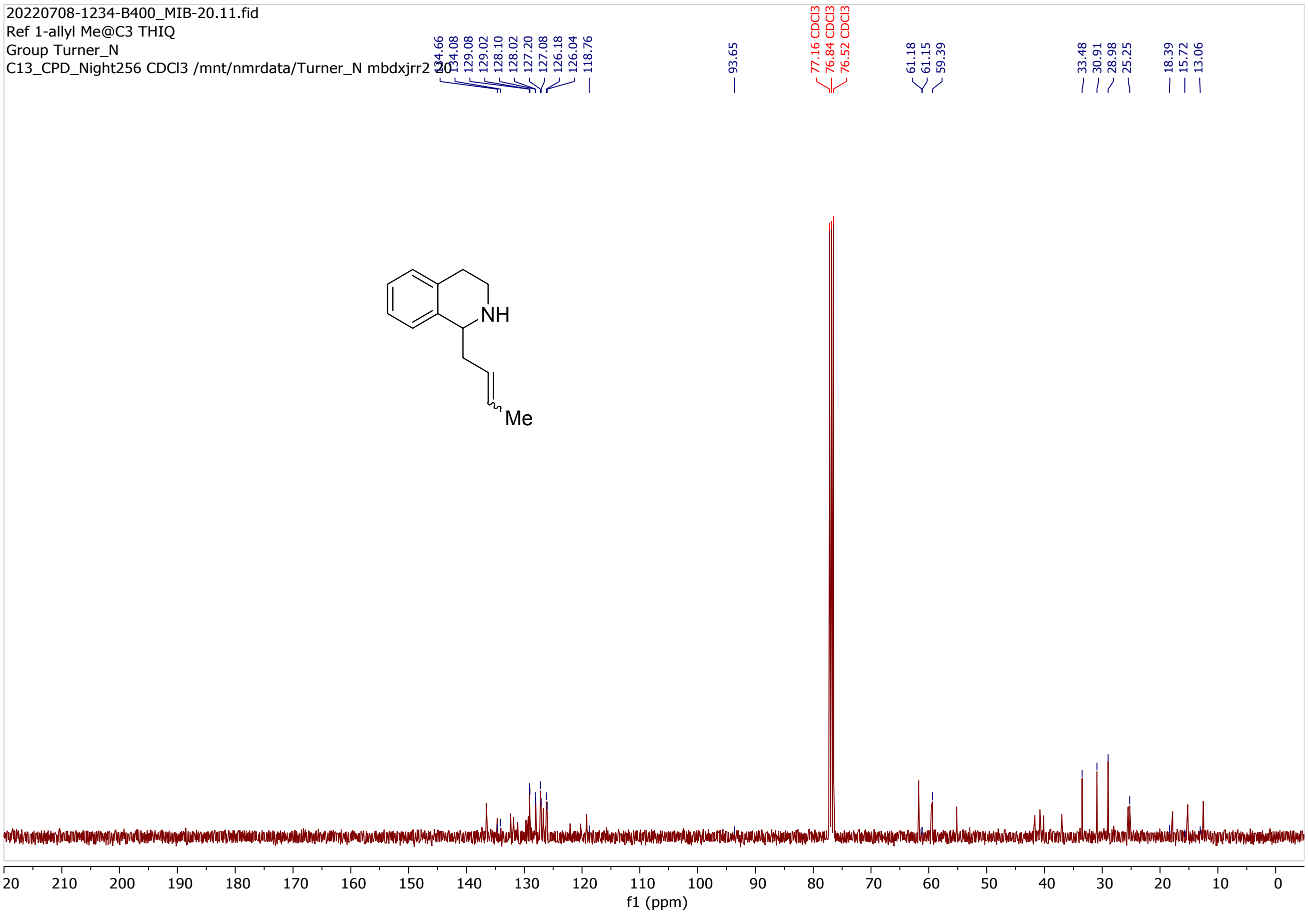

1-(But-3-en-2-yl)-1,2,3,4-tetrahydroisoquinoline, (*rac*)-3p

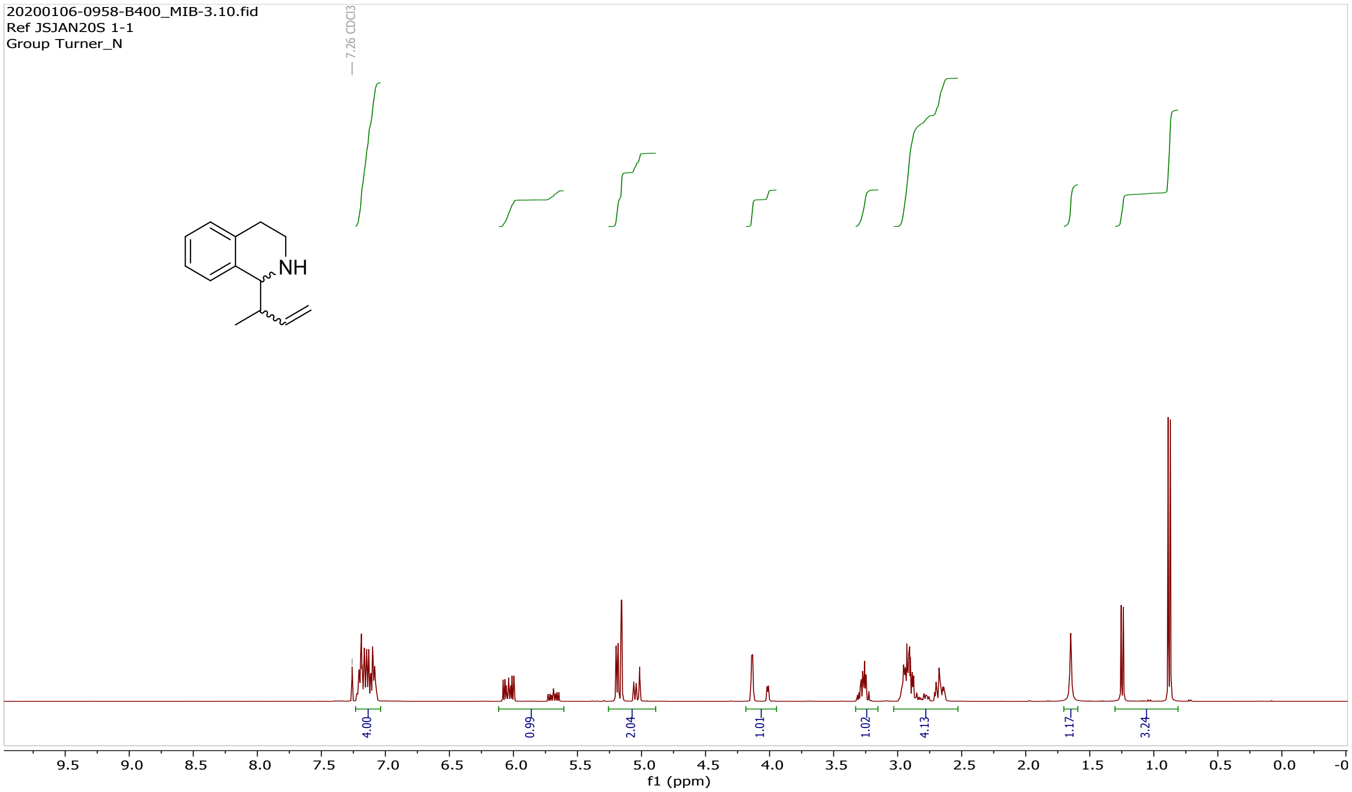

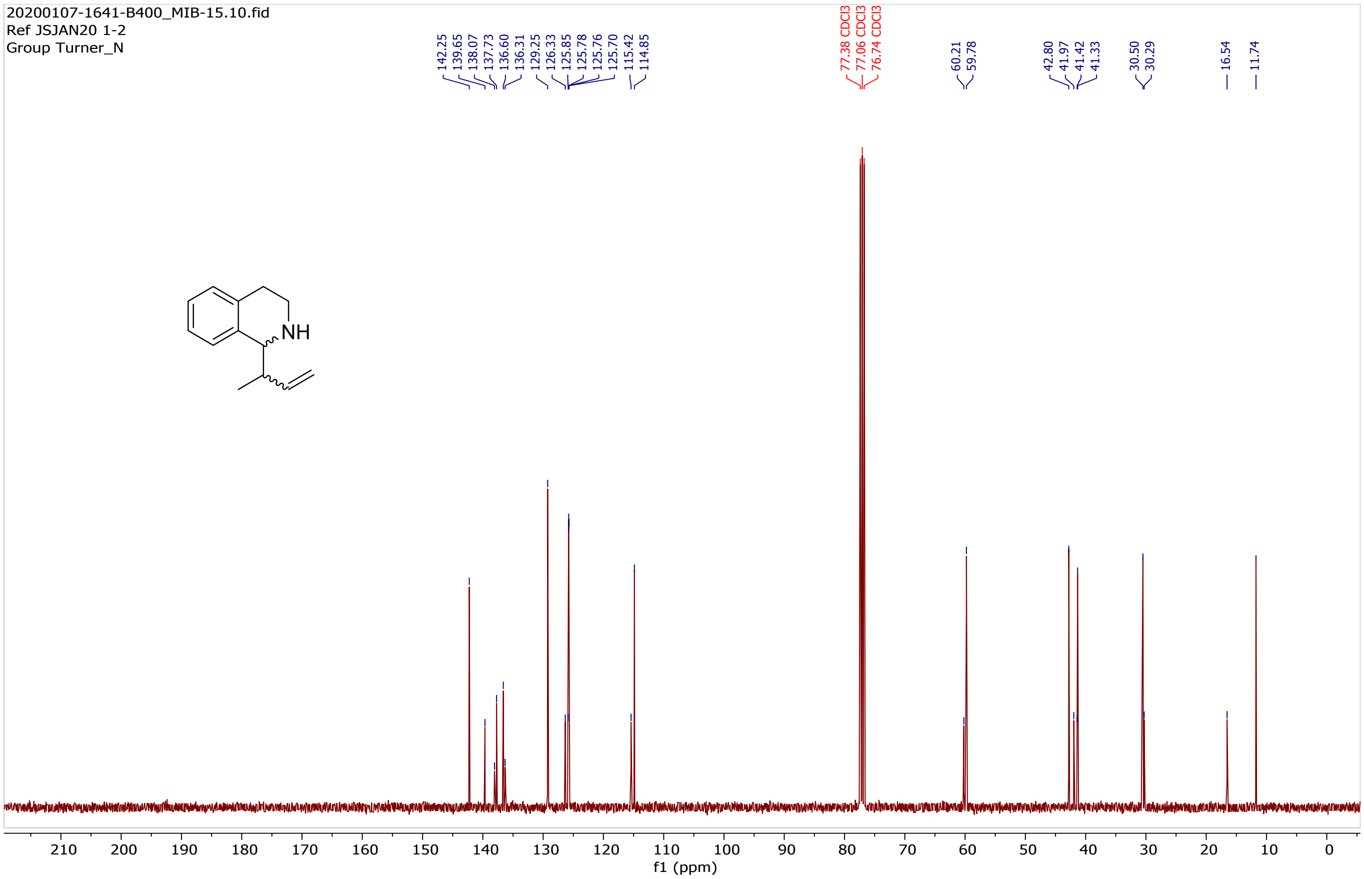

10. References

(1) Batista, V. F.; Galman, J. L.; Pinto, D. C.; Silva, A. M. S.; Turner, N. J. Monoamine Oxidase: Tunable Activity for Amine Resolution and Functionalization. *ACS Catalysis.* ***2018**, pp 11889–11907.* https://doi.org/10.1021/acscatal.8b03525.

(2) Herter, S.; Medina, F.; Wagschal, S.; Benhaïm, C.; Leipold, F.; Turner, N. J. Mapping the Substrate Scope of Monoamine Oxidase (MAO-N) as a Synthetic Tool for the Enantioselective Synthesis of Chiral Amines. *Bioorganic Med. Chem.* ***2018**, 26 (7), 1338–1346.* https://doi.org/10.1016/j.bmc.2017.07.023.

(3) Ghislieri, D.; Green, A. P.; Pontini, M.; Willies, S. C.; Rowles, I.; Frank, A.; Grogan, G.; Turner, N. J. Engineering an Enantioselective Amine Oxidase for the Synthesis of Pharmaceutical Building Blocks and Alkaloid Natural Products. *J. Am. Chem. Soc.* ***2013**, 135 (29), 10863–10869.* https://doi.org/10.1021/ja4051235.

(4) Aleku, G. A.; France, S. P.; Man, H.; Mangas-Sanchez, J.; Montgomery, S. L.; Sharma, M.; Leipold, F.; Hussain, S.; Grogan, G.; Turner, N. J. A Reductive Aminase from Aspergillus Oryzae. *Nat. Chem.* ***2017**, 9 (10), 961–969.* https://doi.org/10.1038/nchem.2782.

(5) Heath, R. S.; Pontini, M.; Bechi, B.; Turner, N. J. Development of an R-Selective Amine Oxidase with Broad Substrate Specificity and High Enantioselectivity. *ChemCatChem* ***2014**, 6 (4), 996–1002.* https://doi.org/10.1002/cctc.201301008.

(6) Zhao, C.; Glazier, D. A.; Yang, D.; Yin, D.; Guzei, Ii. A.; Aristov, M. M.; Liu, P.; Tang, W. Intermolecular Regio- and Stereoselective Hetero-[5+2] Cycloaddition of Oxidopyrylium Ylides and Cyclic Imines. *Angew. Chemie* ***2019**, 131 (3), 897–901.* https://doi.org/10.1002/ange.201811896.

(7) Barna, B.; Gáti, T.; Kotschy, A.; Tasnádi, G. Chemo-Enzymatic One-Pot Two-Step Functionalization of 1,2,3,4-Tetrahydroisoquinolines by Monoamine Oxidase-Ugi-Joullié Reaction Sequence. *European J. Org. Chem.* ***2022**, 2022 (8).* https://doi.org/10.1002/EJOC.202101545.

(8) Soriano, M. D. P. C.; Shankaraiah, N.; Santos, L. S. Short Synthesis of Noscapine, Bicuculline, Egenine, Capnoidine, and Corytensine Alkaloids through the Addition of 1-Siloxy-Isobenzofurans to Imines. *Tetrahedron Lett.* ***2010**, 51 (13), 1770–1773.* https://doi.org/10.1016/J.TETLET.2010.01.104.

(9) Manipulation of Nitrogen- and Sulfur-Containing Substrates: Scope, Generality, and Mechanism of IBX-Mediated Amine Oxidations and Dithiane Deprotections. *J. Am. Chem. Soc.* ***2004**, 126 (16), 5192–5201.* https://doi.org/10.1021/ja0400382.

(10) Wu, T. R.; Chong, J. M. Asymmetric Allylboration of Cyclic Imines and Applications to Alkaloid Synthesis. *J. Am. Chem. Soc.* ***2006**, 128 (30), 9646–9647.* https://doi.org/10.1021/ja0636791.

(11) Fang, Y.; Hu, X.; Shi, Z.; Zhao, X.; Gopireddy, R. R.; Luo, Y. Iron-Catalyzed Stereoselective Allylboration of 3,4-Dihydroisoquinolines with Potassium Allyltrifluoroborates. *Synth.* ***2021**, 53 (19), 3564–3572. https://doi.org/10.1055/a-1493-6420.*

(12) Patel, A.; Barcan, G. A.; Kwon, O.; Houk, K. N. Origins of 1,6-Stereoinduction in Torquoselective 6π Electrocyclizations. *J. Am. Chem. Soc.* ***2013**, 135 (12), 4878–4883.* https://doi.org/10.1021/ja400882y.

(13) Liu, Y.; Li, H.; Chiba, S. Photoinduced Cross-Coupling of Aryl Iodides with Alkenes. *Org. Lett.* ***2021**, 23 (2), 427–432.* https://doi.org/10.1021/acs.orglett.0c03935.

(14) Ma, W.; Fang, J.; Ren, J.; Wang, Z. Lewis Acid Catalyzed Formal Intramolecular [3 + 3] Cross-Cycloaddition of Cyclopropane 1,1-Diesters for Construction of Benzobicyclo[2.2.2]Octane Skeletons. *Org. Lett.* ***2015**, 17 (17), 4180–4183.* https://doi.org/10.1021/acs.orglett.5b01927.

(15) Batista, V. F.; Galman, J. L.; Pinto, D. C.; Silva, A. M. S.; Turner, N. J. Monoamine Oxidase: Tunable Activity for Amine Resolution and Functionalization. *ACS Catalysis.* ***2018**, pp 11889–11907.* https://doi.org/10.1021/acscatal.8b03525.

(16) Herter, S.; Medina, F.; Wagschal, S.; Benhaïm, C.; Leipold, F.; Turner, N. J. Mapping the Substrate Scope of Monoamine Oxidase (MAO-N) as a Synthetic Tool for the Enantioselective Synthesis of Chiral Amines. *Bioorganic Med. Chem.* ***2018**, 26 (7), 1338–1346.* https://doi.org/10.1016/J.BMC.2017.07.023.

(17) Ghislieri, D.; Green, A. P.; Pontini, M.; Willies, S. C.; Rowles, I.; Frank, A.; Grogan, G.; Turner, N. J. Engineering an Enantioselective Amine Oxidase for the Synthesis of Pharmaceutical Building Blocks and Alkaloid Natural Products. *J. Am. Chem. Soc.* ***2013**, 135 (29), 10863–10869.* https://doi.org/10.1021/ja4051235.

(18) Yus, M.; González-Gómez, J. C.; Foubelo, F. Diastereoselective Allylation of Carbonyl Compounds and Imines: Application to the Synthesis of Natural Products. *Chemical Reviews.* ***2013****, pp 5595–5698.* https://doi.org/10.1021/cr400008h.

(19) Harawa, V.; Thorpe, T. W.; Marshall, J. R.; Gilio, A.; Heath, R. S.; Angelastro, A.; Finnigan, J. D.; Charnock, S. J.; Grogan, G.; Whitehead, R. C.; Turner, N. J.; Affiliations, *. Synthesis of Stereoenriched Piperidines via Chemo-Enzymatic Dearomatization of Activated Pyridines; **2021**. In submission.
